# Diversity and biogeography of land snails (Mollusca, Gastropoda) in the limestone hills of Perak, Peninsular Malaysia

**DOI:** 10.3897/zookeys.682.12999

**Published:** 2017-07-04

**Authors:** Junn Kitt Foon, Gopalasamy Reuben Clements, Thor-Seng Liew

**Affiliations:** 1 Institute for Tropical Biology and Conservation, Universiti Malaysia Sabah, Jalan UMS, 88400 Kota Kinabalu, Sabah, Malaysia; 2 Rimba, 22-3A, Casa Kiara 2, Jalan Kiara 5, 50480 Kuala Lumpur, Malaysia; 3 Department of Biological Sciences, Sunway University, No. 5 Jalan Universiti, 47500 Bandar Sunway, Selangor, Malaysia

**Keywords:** Biogeography, checklist, conservation, endemism, karst, Kinta River, mollusc, Perak River

## Abstract

Limestone hills are now gaining global conservation attention as hotspots for short-range endemic species. Levels of land snail endemism can be high at limestone hills, especially at hill clusters that are geographically isolated. In the State of Perak, Peninsular Malaysia, limestone hills have been opportunistically surveyed for land snails in the past, but the majority have yet to be surveyed. To address this knowledge gap, we systematically surveyed the terrestrial malacofauna of 12 limestone hills that, based on our opinion, are a representation of the limestone land snail assemblages within the State. Our inventory yielded high sampling completeness (>85%). We found 122 species of land snails, of which 34 species were unique to one of the surveyed hills. We identified 30 species that are potentially new to science. The number of land snail species recorded at each hill ranged between 39 and 63 species. Four of the sampled limestone hills namely, Prk 01 G. Tempurung, Prk 55 G. Pondok, Prk 47 Kanthan, and Prk 64 Bt Kepala Gajah, have high levels of species richness and unique species, representing 91% of the total species recorded in this study. We identified two clusters of limestone hills in central Perak with distinct differences in land snail species composition – a northern hill cluster on elevated granite bedrock and southern hill cluster in a low-lying valley surrounded by alluvial soils. As limestone hills continue to be quarried to meet the cement demand, the four identified limestone hills, along with other hills from the two clusters, warrant urgent conservation attention in order to maintain high species diversity within Perak’s terrestrial malacofauna.

## Introduction

Limestone hills are popularly known as “arks of biodiversity” because they contain high levels of species endemism, especially in Peninsular Malaysia where these hills only cover 0.2 % of the total land area (Schilthuizen 2004; Clements et al. 2006; Chua et al. 2009; Liew et al. 2014).

**Figure 1.**
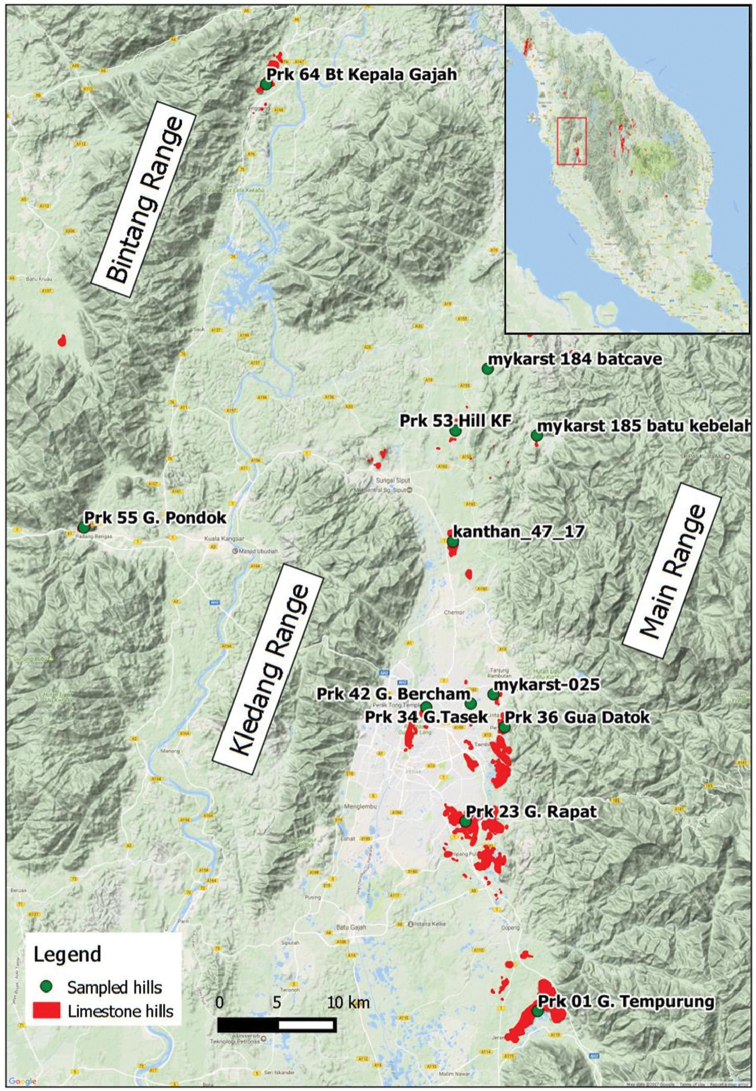
The 12 limestone hills sampled in and around the Kinta Valley of Perak, Peninsular Malaysia.

To date, at least 445 limestone hills have been documented in Peninsular Malaysia, with the majority located in the States of Kelantan (149 hills), Pahang (124 hills) and Perak (93 hills) (Liew et al. 2016). The State of Perak has the third largest number of limestone hills, but it has the largest number of operating quarries (Liew et al. 2016). The majority of these hills can be found within the Kinta Valley, with some other hills scattered around the northern part of the Kinta Valley and Bintang Range (Figure 1). Over the past decade, the endemism of different plant and animal at the limestone hills of Perak has drawn attention from evolutionary biologists (Clements et al. 2008a), conservationists (Clements et al. 2008b) and concerned taxonomists (Kiew et al. 2014; Vermeulen and Marzuki 2014). Given the large number of limestone hills in Perak, it is not practical to spare every hill from quarrying. Thus, conservation prioritisation needs to be conducted, ideally based on the biogeographical patterns of endemic taxa such as land snails (Clements et al. 2008b).

The limestone hills in Perak form an important region because that was where the foundation for land snail knowledge in Peninsular Malaysia was laid. More than 50 land snail species have been described from this State alone; these species descriptions were the outputs of three main faunistic studies by separate groups of naturalists and malacologists. The first was an expedition to central Perak by de Morgan (1885a), who described 33 new land snail species from the area. Second was the work by von Möllendorff, who published a species checklist of around 60 species and described 15 new species based on the collection by R. Hungerford at Gunung Larut (von Möllendorff 1886, 1891). Third was the survey done by E. Townsend at Gunung Pondok, where around 13 new species were described (Godwin-Austen and Nevill 1879; Crosse 1879a, 1879b). Besides these faunistic surveys in Perak, at least 20 new species were described based on taxonomic studies of particular land snail taxa or random sampling (van Benthem Jutting 1952, 1954, 1961a, 1961b; Tomlin 1938, 1939, 1941; Fulton 1901, 1902; Sykes 1903; Godwin-Austen 1909). More recent studies suggest that about one-third on the known species from Peninsular Malaysia can be found in Perak, particularly around the Kinta Valley (Davison 1991; Chan 1998a, 1998b; Clements et al. 2008b; Maassen 2001). In Perak, land snail species richness on a hill can reach as high as 53 species (Clements et al. 2008b).

Despite intensive land snail research conducted in the State of Perak, the land snail inventory is far from complete. Most of the comprehensive surveys only report the name of the species, without illustrations of the species, or with provisional working species names, such as sp. 1, sp. 2. (e.g. Davison 1991; Clements et al. 2008b). This has made it difficult to update current checklists because species identities in previous reports cannot be used to compare with recently collected specimens. Furthermore, many of these unnamed species in previous reports are probably new species yet to be described or are doubtful species that require taxonomic revision. Hence, good annotated checklists of land snails with traceable specimens and high quality illustrations are key to improve the current knowledge of land snails in Perak and Peninsular Malaysia in general.

The biogeography of organisms on limestone hills in Perak is also poorly known. In Peninsular Malaysia, land snail communities on limestone hills can generally be divided into two groups, one on the hills along the east coast and the other on the hills along the west coast, both of which are separated by Main Range in the centre of the peninsula (van Benthem Jutting 1960; Clements et al. 2008b). Within the State of Perak, Davison (1991) noticed land snails on limestone hills can be further divided into two groups, one on the hills within Kinta Valley and the other one on the hills scattered in the area north of Kinta Valley, though based on an incomplete land snail inventory (Davison 1991: 6 – 35 species from 12 hills, averaging 17 species per hill, compared to Clements et al. 2008b: 32 – 53 species from 6 hills, averaging 42 species per hill). As such, further quantitative analysis on the biogeographical patterns of land snails with more comprehensive species inventories from limestone hills that are representative of Perak’s terrestrial malacofauna can contribute to improved limestone conservation planning within the State.

Limestone hills have been recently highlighted as one of Malaysia’s vulnerable ecosystems due to surrounding forest degradation and quarrying activities (Ministry of Natural Resources and Environment Malaysia 2016). Many land snail species that are endemic to limestone hills in Peninsular Malaysia are already extinct or on the brink of extinction (http://www.iucnredlist.org/search/link/57e5b1fd-2bc5b54c). In order to prevent further species extinctions, limestone hills that are currently being quarried or intact must be urgently assessed for land snail diversity.

Here, we conduct a land snail inventory in and around the Kinta Valley of Perak to: (1) provide an annotated checklist of land snail species for the State, along with photographic images for each species; and (2) elucidate land snail diversity and biogeographical patterns across 12 limestone hills within the State. For the second objective, we specifically examined relationships between limestone hill parameters (size and isolation) and species richness and biogeographical patterns of land snails. Finally, we discuss the conservation implications of our study for limestone hills in Perak.

## Methodology

### Sampling design and sites

In addition to six limestone hills previously surveyed by Clements et al. (2008b), six other limestone hills were included in this study. Among the six previously sampled hills, only one (i.e. Gunung Bercham) was resampled because of the low sampling completeness reported by Clements et al. 2008b. Sampling was conducted between 16/8/2016 and 24/8/2016. Our sampling approach consisting of 12 limestone hills will (1) result in the sampling of more hills in clusters that have a larger number of limestone hills; and (2) provide wider geographical coverage of limestone hills across the Kinta Valley (Table 1; Figure 1).

**Table 1.** The geographical coordinates, size, isolation of 12 limestone hills sampled in this study in and around the Kinta Valley of Perak. The names of the limestone hills follow a standardized national code developed by Liew et al. (2016).

**No.**	**Limestone hills**	**Longitude / Latitude**	**Size (km^2^)**	**Degree of Isolation^@^**	**Species richness**	**Unique species**
1	Gunung Kanthan*	4.761388, 101.1210	0.827	5	63	6
2	mykarst-025*	4.653244, 101.1539	0.122	13	40	1
3	Prk 1 G. Tempurung*	4.415082, 101.1877	8.722	6	54	9
4	Prk 23 G. Rapat^	4.552856, 101.1311	5.379	17	45	3
5	Prk 34 G. Tasek^	4.643467, 101.0998	0.114	13	45	1
6	Prk 36 Gua Datok^	4.627492, 101.1581	0.644	16	49	1
7	Prk 53 Hill KF^	4.854229, 101.1225	0.038	8	44	1
8	Prk 55 G. Pondok^	4.786687, 100.8402	0.662	1	50	5
9	Prk 64 Bt Kepala Gajah*	5.118655, 100.9727	1.075	7	45	7
10	mykarst-184 Bat Cave*	4.906175, 101.1467	0.014	6	50	0
11	mykarst-185 Batu Kebelah*	4.853657, 101.1864	0.022	4	39	0
12	Prk 42 G. Bercham #	4.644832, 101.1338	0.059	16	45	0

* This study, ^ Clements et al. (2008), # combined data of this study and Clements et al. (2008)

^@^ Degree of isolation of each limestone hill – number of other hills within its 10 km radius (see Liew et al. 2016).

### Land snails sampling and processing

In each of the seven limestone hills, four 2 m × 4 m plots were established (Clements et al. 2008b; Liew et al. 2008; Suppl. material 1). In each plot, a total of five litres of top soil and leaf litter were collected for extraction of micro-snails (< 5 mm) and the plot was searched for macro-snails. Upon returning to the laboratory, all macro-snails were cleaned with running water and then dried in an oven.

After that, shells were extracted from soil samples by manually picking up the shells under a stereomicroscope (Liew et al. 2008). For species identification, a complete literature of Malay Peninsular terrestrial mollusca was consulted, with emphasis on the most recent species compilation and overview by Maassen (2001). All specimens from recent sampling and previous studies (Clements et al. 2008b) were identified to morphospecies based on a combination of photographs, illustrations and description of types. Morphospecies that could not be assigned to an available name were given working morphospecies names (for example, *Acmella* ‘Kanthan 1’). Species reported to be present in our study sites in the literature, but not found during our sampling, were not included in our results. All specimens were catalogued in the *BORNEENSIS* Mollusca collection database and were deposited in the *BORNEENSIS* collection of Institute for Tropical Biology and Conservation, Universiti Malaysia Sabah.

### Data analysis

A species checklist was compiled for the 12 limestone hills and was arranged according to the classification of Maassen (2001). Under species or subspecies, four subsections were provided: (1) Reference to figures; (2) Materials examined, which includes only species sampled from this study (with the exception of some species where poor shell preservation warranted a substitute specimen from other studies for photographic purposes); (3) Distribution, which is separated into distribution within Peninsular Malaysia and distribution elsewhere; (4) Remarks, which includes brief diagnosis, comparison with conspecifics and note on new records. All species are considered native unless noted as synantropic in Remarks.

For our analysis of land snail diversity patterns, we first assessed species diversity in terms of (1) species richness – total number of species for each hill; and (2) unique species – species found only in one of the twelve limestone hills in this study. Next, we assessed whether there was a correlation between the species richness and the number of unique species for the twelve hills. We also examined relationships between species diversity and limestone hill parameters (i.e. size and degree of isolation). Depending on the normality test of data for each parameter, Spearman or Pearson correlation coefficients were used to test for correlations. Analysis was conducted using built-in function in the R statistical environment v. 3.3.1 (R Core Team 2016).

For our analysis of land snail biogeographical patterns, land snail data were tabulated into a data matrix of 122 species × 12 limestone hills that consisted of absence (0)/presence (1) data for each species on each of the twelve hills. We evaluated sampling completeness for all 12 hills and compared their species composition to determine their degree of similarity. A cluster analysis was performed to objectively assign limestone hills into clusters so that hills within each cluster are similar to one another with respect to overall land snail composition. In our analysis, a dissimilarity distance matrix was calculated using the Jaccard similarity coefficient (i.e. Jaccard index) based on the data matrix. Next, hierarchical clustering on the 12 sites based on the dissimilarity matrix was performed using the method of complete linkage. Analysis was conducted using package ‘vegan’ (Dixon 2003) and ‘iNEXT’ (Hsieh et al. 2016) in the R statistical environment v. 3.3.1 (R Core Team 2016).

## Results

### Diversity and biogeography of land snails

We achieved high sampling completeness (> 85 %) for all seven limestone hills sampled in our study (Suppl. material 3). The species richness and number of unique species that can be found only in a single hill is listed in Table 1. The number of land snail species recorded in each hill ranges between 39 species and 63 species. Gunung Kanthan has the highest number of species – 63 species with 6 unique species, and followed by Gunung Tempurung – 54 species with 9 unique species. The list of 34 unique species found in each limestone hills is listed in Table 2. The species richness and number of unique species of each hill were also mapped to understand land snail diversity patterns among the limestone hills in and around the Kinta Valley of Perak (Table 1)

**Table 2.** Unique species for each of 12 limestone hills (and species richness [SR]) in and around the Kinta Valley of Perak, Peninsular Malaysia.

**Prk 01 G. Tempurung (SR = 54)**	**Prk 55 G. Pondok (SR = 50)**
*Glyptaulax* ‘tempurung 1’	*Chamalycaeus microconus* (von Möllendorff, 1886)
*Macrochlamys* ‘tempurung 1’	*Chamalycaeus oligopleuris* (von Möllendorff, 1886)
*Macrochlamys* ‘tempurung 2’	*Lagochilus* ‘pondok 1’
*Microcystina* ‘tempurung 2’	*Microcystina* ‘pondok 1’
*Microcystina* ‘tempurung 3’	*Sinoennea perakensis* (Godwin-Austen & Nevill, 1879)
Opisthostoma cf. vermiculum	
*Opisthostoma* ‘tempurung 1 detached’	
*Paraboysidia* ‘tempurung 1’	**Prk 23 G. Rapat (SR = 45)**
*Rahula* ‘tempurung 1’	Arinia (Notharinia) micro Marzuki & Foon, 219
	*Opisthostoma megalomphalum* van Benthem Jutting, 1955
**Prk 47 Kanthan (SR = 63)**	*Opisthostoma vermiculum* Clements & Vermeulen, 2008
*Chamalycaeus* ‘Kanthan 1’	
*Chamalycaeus* ‘kanthan 2’	
Diplommatina cf. diminuta	**Prk 36 Gua Datok (SR = 49)**
*Lagochilus townsendi* Crosse, 1879a	*Pupina arula perakensis* von Möllendorff, 1896
Opisthostoma cf. gittenbergeri	
Opisthostoma cf. subconicum	
	**Prk 34 G.Tasek (SR = 45)**
	Arinia (Notharinia) ‘tasek 1’
**Prk 64 Bt Kepala Gajah (SP = 45)**	**mykarst-025 (SR = 40)**
*Diplommatina lenggongensis* Tomlin, 1941	*Opisthostoma* ‘mykarst-25 1’
*Microcystina* ‘guatokgiring 1’	
*Microcystina* ‘guatokgiring 2’	**Prk 53 Hill KF (SR = 44)**
*Opisthostoma castor* van Benthem Jutting, 1961	*Sinoennea* ‘prk53 1’
*Sinoennea* ‘guatokgiring 1’	
*Sinoennea lenggongensis* Tomlin, 1948	**mykarst-184 Bat Cave (SP = 50)**
*Sinoennea tweediei* Tomlin, 1941	None.
**Prk 42 G. Bercham (SP = 45)**	**mykarst-185 Batu Kebelah (SP = 39)**
None.	None.

**Figure 2.**
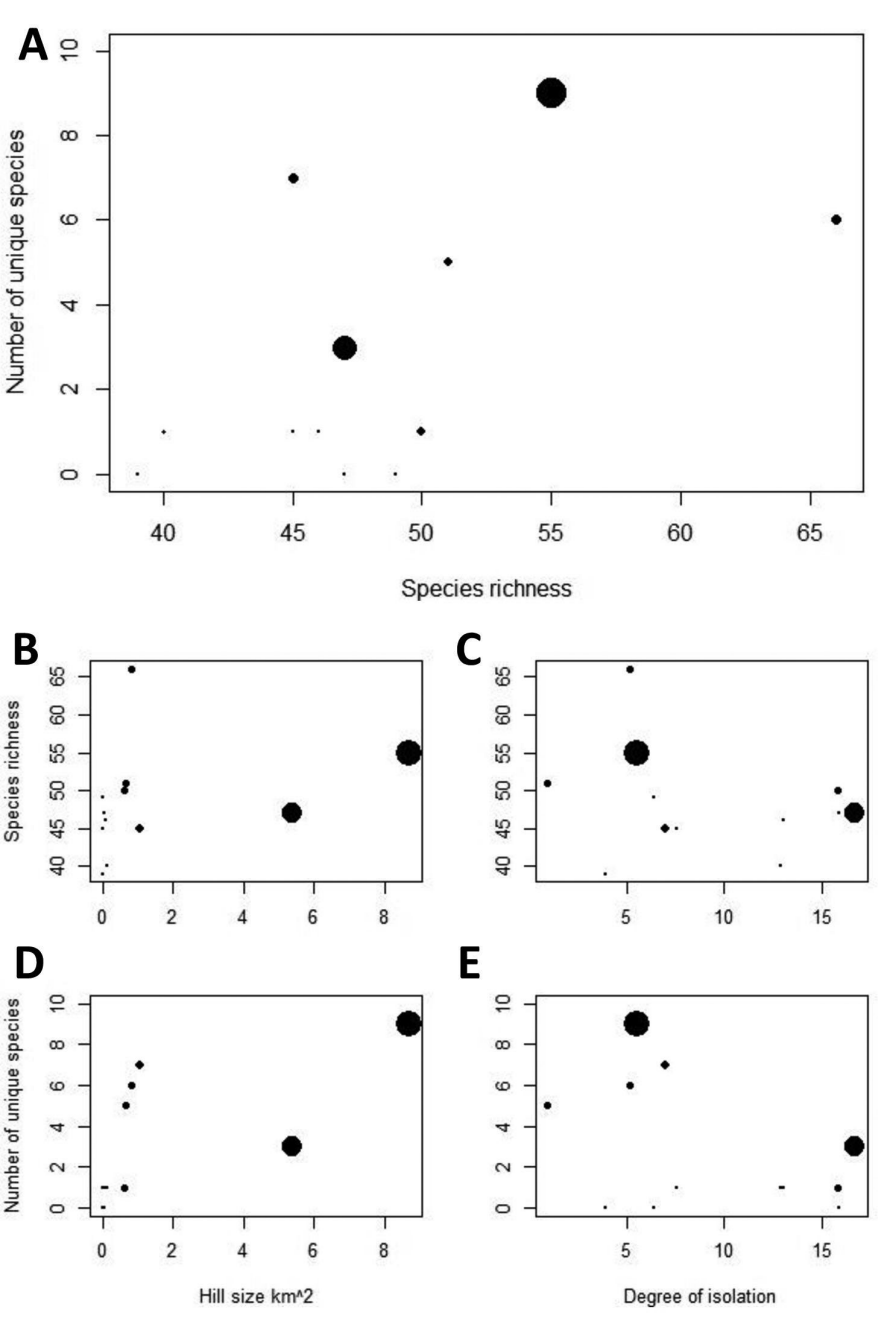
Relationships between limestone physical parameters and land snail species diversity of the 12 limestone hills in Perak, Malaysia. Limestone hills size indicated by relative point size in the plot. **A** Relationship between species richness and number of unique species **B** Relationship between species richness and limestone hills size (km^2^) **C** Relationship between species richness and degree of isolation of the limestone hills **D** Relationship between number of unique species and limestone hills size (km^2^) **E** Relationship between number of unique species and degree of isolation of the limestone hills.

Species richness and the number of unique species were not correlated among the 12 hills (p = 0.052, Figure 2A). There appears to be no relationship between the species richness and limestone hill size (p = 0.12) and isolation (p = 0.5) (Figure 2B, C). On the other hand, the number of unique species was strongly correlated with limestone hill size (r_s_ = 0.91, p = 0.0005), but not isolation (p=0.43) (Figure 2D, E).

**Figure 3.**
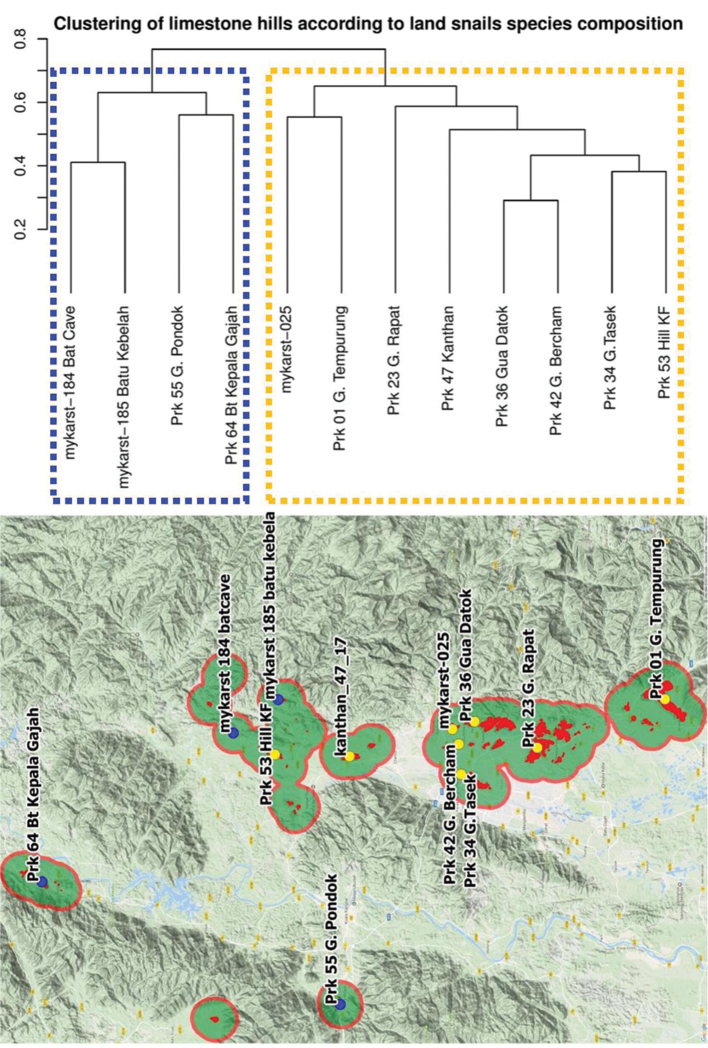
Cluster analysis of land snail species composition of 12 limestone hills in and around the Kinta Valley of Perak, Peninsular Malaysia. Two clusters were identified: a northern (blue dots) and southern cluster (yellow dots). Green polygons with red boundary refer to major clusters of limestone hills in Kinta Valley identified based on the 2.5 km buffer analysis (i.e. at least 2.5 km from the nearest cluster/hills). Red dots indicate limestone hills not sampled in this study.

Cluster analysis identified two clusters of limestone hills based on land snail species composition: a northern cluster and southern cluster (Figure 3). The northern cluster comprises four limestone hills: Prk 64 Bt. Kepala Gajah, Prk 55 G. Pondok, mykarst-184 Bat Cave, and mykarst-185 Batu Kebelah. The Southern cluster consists of the remaining limestone hills in the Kinta Valley (Figure 3).

### Checklist

In total, we recorded 122 species from the 12 limestone hills sampled in and around the Kinta Valley of Perak (Suppl. material 2). This checklist consists of 23 families and 47 genera. There are 30 out of 122 species identified from this project which could not be assigned to scientific names that are currently published. Some of these unnamed species are potentially new to science but systematic taxonomic revisions need to be done as many of these groups have not been critically revised. The most diverse genera were *Opisthostoma* (13 species), *Microcystina* (10 species), and *Diplommatina* (9 species).

### Class Gastropoda Cuvier, 1795

#### Clade Caenogastropoda Cox, 1960

##### Family Assimineidae Adams & Adams, 1856

###### Genus *Acmella* Blanford, 1869

####### Acmella

Taxon classificationAnimaliaLittorinimorphaAssimineidae

‘Kanthan 1’

Figure 4A

######## Materials examined.

Prk 47 Kanthan: BOR/MOL 9079, BOR/MOL 9157.mykarst-184 Bat Cave: BOR/MOL 9781, BOR/MOL 9839, BOR/MOL 12502. Prk 64 Bt Kepala Gajah: BOR/MOL 10192. Prk 23 G. Rapat: BOR/MOL 10237. Prk 36 Gua Datok: BOR/MOL 10453. Prk 42 G. Bercham: BOR/MOL 10583, BOR/MOL 12497, BOR/MOL 12498, BOR/MOL 12503. Prk 53 Hill KF: BOR/MOL 10784. Prk 01 G. Tempurung: BOR/MOL 11397, BOR/MOL 12501.

######## Distribution.

Known from Kinta Valley and Lenggong, Perak only.

######## Remarks.

Very small shell. Tall spire, conical shaped shell. Radial ribs prominent, chevron-shaped with the pointed end in the opposite direction of shell growth. *Acmella roepstorffiana* Nevill, 1878 from Pahang differs in radial rib shape.

**Figure 4.**
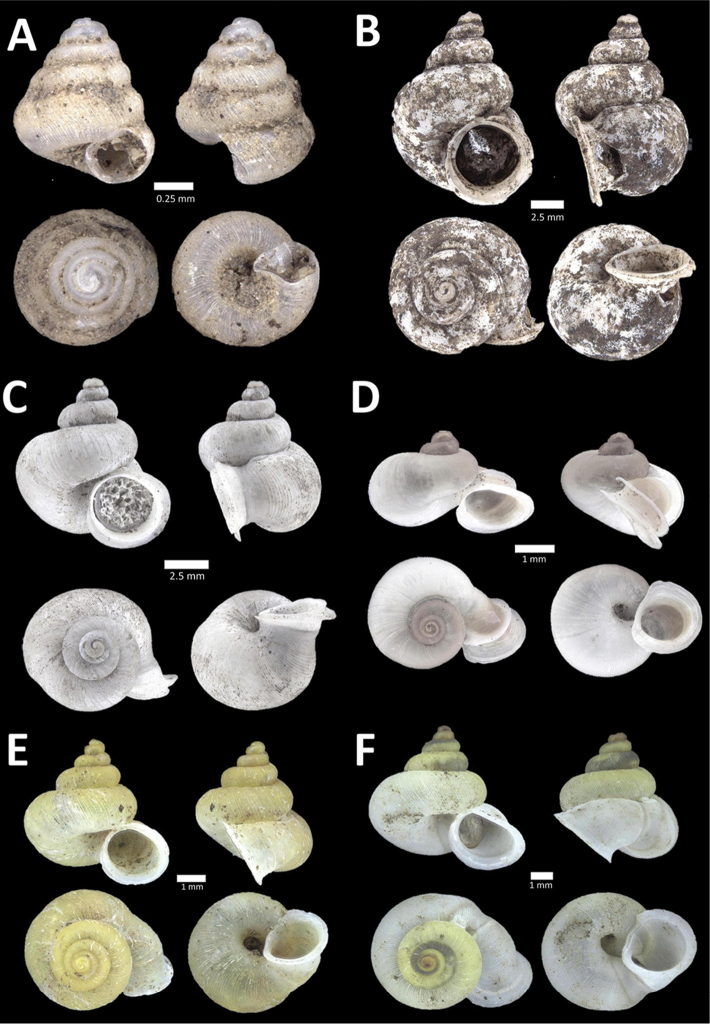
A. *Acmella* ‘Kanthan 1’ BOR/MOL 9781. Perak, Ipoh, Bat Cave Hill Plot 1 **B** *Alycaeus conformis* Fulton, 1902 BOR/MOL 10057. Perak, Ipoh, Gunung Rapat Plot C3 **C** *Alycaeus gibbosulus* (Stoliczka, 1872) BOR/MOL 9436. Perak, Ipoh, Mykarst-025 Plot 3 **D** *Alycaeus jousseaumei* (de Morgan, 1885a) BOR/MOL 11211. Perak, Ipoh, Gunung Tempurung Plot 3 **E** *Alycaeus kapayanesis* (de Morgan, 1885a) BOR/MOL 11385. Perak, Ipoh, Gunung Tempurung Plot 2 **F** *Alycaeus perakensis* (Crosse, 1879) BOR/MOL 11506. Perak, Ipoh, Gunung Pondok, plot 3.

##### Family Cyclophoridae Gray, 1847

###### Genus *Alycaeus* Baird, 1850

####### Alycaeus conformis

Taxon classificationAnimaliaArchitaenioglossaCyclophoridae

Fulton, 1902

Figure 4B

######## Materials examined.

Prk 23 G. Rapat: BOR/MOL 10057.

######## Distribution.

In Peninsular Malaysia, known from Perak, Selangor and Kelantan (Maassen 2001). Elsewhere, in Tenasserim (=Tanintharyi), Myanmar and Salang (=Phuket), Thailand (Maassen 2001).

######## Remarks.

Small shell. Globular, convex whorls. Very similar to *Alycaeus gibbosulus*, distinguished only by the less expanded penultimate whorl and clean operculum exterior.

####### Alycaeus gibbosulus

Taxon classificationAnimaliaArchitaenioglossaCyclophoridae

(Stoliczka, 1872)

Figure 4C

######## Materials examined.

mykarst-025: BOR/MOL 9382, BOR/MOL 9416, BOR/MOL 9436, BOR/MOL 9500. mykarst-027: BOR/MOL 9106. Prk 23 G. Rapat: BOR/MOL 10286. Prk 55 G. Pondok: BOR/MOL 11523, BOR/MOL 11538.

######## Distribution.

Found throughout Peninsular Malaysia. Elsewhere, in southern Thailand (Maassen 2001).

######## Remarks.

Small shell. Globular, convex whorls. Very similar to *Alycaeus conformis*, differs only by the more expanded penultimate whorl and calcrete-encrusted operculum exterior.

####### Alycaeus jousseaumei

Taxon classificationAnimaliaArchitaenioglossaCyclophoridae

(de Morgan, 1885a)

Figure 4D

######## Materials examined.

Prk 53 Hill KF: BOR/MOL 10717, BOR/MOL 10658, BOR/MOL 10689. Prk 47 Kanthan: BOR/MOL 9374, BOR/MOL 9053. mykarst-025: BOR/MOL 9383. mykarst-027: BOR/MOL 9101. Prk 23 G. Rapat: BOR/MOL 10228, BOR/MOL 10046, BOR/MOL 10202, BOR/MOL 10253. Prk 36 Gua Datok: BOR/MOL 10412, BOR/MOL 10441, BOR/MOL 10474, BOR/MOL 10494. Prk 42 G. Bercham: BOR/MOL 10593. Prk 34 G. Tasek: BOR/MOL 10788, BOR/MOL 11054. Prk 01 G. Tempurung: BOR/MOL 11136, BOR/MOL 11211, BOR/MOL 11408.

######## Distribution.

Restricted to Kinta Valley.

######## Remarks.

Small shell. Convex whorls. Easily distinguished from congeners by its flat and highly expanded penultimate whorl with double peristomal expansion.

####### Alycaeus kapayanesis

Taxon classificationAnimaliaArchitaenioglossaCyclophoridae

(de Morgan, 1885b)

Figure 4E

######## Materials examined.

Prk 47 Kanthan: BOR/MOL 9081, BOR/MOL 9161. mykarst-027: BOR/MOL 9041. mykarst-025: BOR/MOL 9425. Prk 23 G. Rapat: BOR/MOL 10230, BOR/MOL 10031, BOR/MOL 10259. Prk 53 Hill KF: BOR/MOL 10671, BOR/MOL 10696. Prk 36 Gua Datok: BOR/MOL 10482, BOR/MOL 10502. Prk 42 G. Bercham: BOR/MOL 10629. Prk 34 G. Tasek: BOR/MOL 10793, BOR/MOL 11028, BOR/MOL 11065. Prk 01 G. Tempurung: BOR/MOL 11142, BOR/MOL 11229, BOR/MOL 11385, BOR/MOL 11414.

######## Distribution.

Restricted to Kinta Valley.

######## Remarks.

Small shell. Tall spire, conical shell. Peristome expanded and reflected. Shell lemon yellow. Shell smaller and penultimate whorl less expanded than *Alycaeus perakensis*.

####### Alycaeus perakensis

Taxon classificationAnimaliaArchitaenioglossaCyclophoridae

(Crosse, 1879a)

Figure 4F

######## Materials examined.

mykarst-184 Bat Cave: BOR/MOL 9877, BOR/MOL 9799, BOR/MOL 9833, BOR/MOL 9770. mykarst-185 Batu Kebelah: BOR/MOL 9546, BOR/MOL 9565, BOR/MOL 9573, BOR/MOL 9585. Prk 64 Bt Kepala Gajah: BOR/MOL 10088, BOR/MOL 10124, BOR/MOL 10136, BOR/MOL 10166. Prk 55 G. Pondok: BOR/MOL 11506, BOR/MOL 11479, BOR/MOL 11539, BOR/MOL 11563.

######## Distribution.

Known from Upper Kinta Valley and Perak River Valley, Perak only.

######## Remarks.

Shell larger than *Alycaeus kapayanensis*. Tall spire, conical shell. Penultimate whorl more expanded than *A. kapayanensis*. Peristome expanded and reflected. Shell lemon yellow.

####### Alycaeus thieroti

Taxon classificationAnimaliaArchitaenioglossaCyclophoridae

de Morgan, 1885a

Figure 5A

######## Materials examined.

mykarst-025: BOR/MOL 9415, BOR/MOL 12422. Prk 23 G. Rapat: BOR/MOL 10056, BOR/MOL 10258.

######## Distribution.

Known from Perak, Selangor and Kelantan only (Maassen 2001).

######## Remarks.

Small shell. Radial ribs prominent, at equidistant. Spiral sculpture fine, indistinct. Peristome expanded and reflected. Differs from other Perak congeners by its more globose whorls.

**Figure 5.**
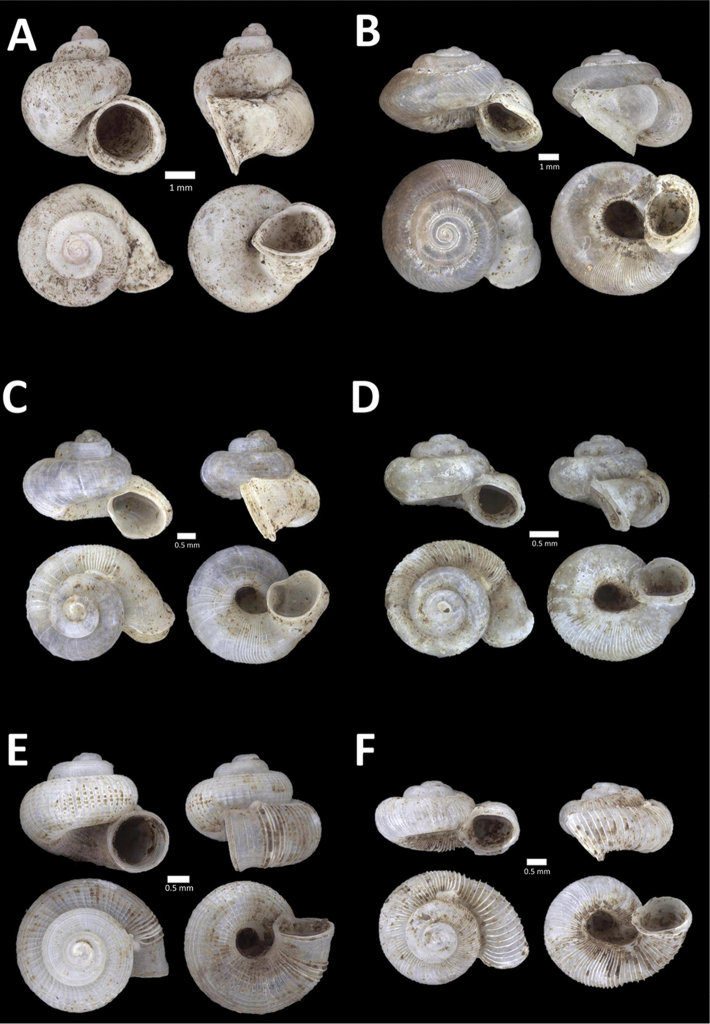
**A** *Alycaeus thieroti* de Morgan, 1885a BOR/MOL 10258. Perak, Ipoh, Gunung Rapat Plot C7 **B** *Chamalycaeus diplochilus* (von Möllendorff, 1886) BOR/MOL 10098. Perak, Ipoh, Gua Tok Giring **C** *Chamalycaeus* ‘Kanthan 1’ BOR/MOL 9127. Perak, Ipoh, Gunung Kanthan Plot 2 **D** *Chamalycaeus* ‘kanthan 2’ BOR/MOL 9160. Perak, Ipoh, Gunung Kanthan Plot 4 **E** *Chamalycaeus microconus* (von Möllendorff, 1886) BOR/MOL 11485. Perak, Ipoh, Gunung Pondok **F** *Chamalycaeus microdiscus* (von Möllendorff, 1886) BOR/MOL 10206. Perak, Ipoh, Gunung Rapat.

###### Genus *Chamalycaeus* Kobelt & von Möllendorff, 1897

####### Chamalycaeus diplochilus

Taxon classificationAnimaliaArchitaenioglossaCyclophoridae

(von Möllendorff, 1886)

Figure 5B

######## Materials examined.

mykarst-184 Bat Cave: BOR/MOL 9885. Prk 47 Kanthan: BOR/MOL 9080, BOR/MOL 9150. mykarst-027: BOR/MOL 9038, BOR/MOL 9129. Prk 64 Bt Kepala Gajah: BOR/MOL 10098, BOR/MOL 10137, BOR/MOL 10175. Prk 42 G. Bercham: BOR/MOL 10586. Prk 55 G. Pondok: BOR/MOL 11522, BOR/MOL 11484.

######## Distribution.

In Peninsular Malaysia, known from Perak and Kelantan (Maassen 2001). Elsewhere, in Jalor (=Yala), Thailand (Maassen 2001).

######## Remarks.

Distinguished from congeners by its larger, low spired shell. Glossy surface. Periphery prominently keeled. Radial ribs prominent along the section of the whorl parallel to the pneumatophore, indistinct elsewhere. Shape similar but shell larger than *Chamalycaeus oligopleuris*.

####### Chamalycaeus

Taxon classificationAnimaliaArchitaenioglossaCyclophoridae

‘Kanthan 1’

Figure 5C

######## Materials examined.

mykarst-027: BOR/MOL 9039, BOR/MOL 9127.

######## Distribution.

Known from mykarst-027 only, but surrounding hills have yet to be adequately surveyed.

######## Remarks.

Shell shares similar prominent, widely spaced-out radial and spiral ridges with *Chamalycaeus oligopleuris* but differs in having taller spire and rounder periphery.

####### Chamalycaeus

Taxon classificationAnimaliaArchitaenioglossaCyclophoridae

‘kanthan 2’

Figure 5D

######## Materials examined.

Prk 47 Kanthan: BOR/MOL 9082, BOR/MOL 9160.

######## Distribution.

Known from Gunung Kanthan only, but surrounding hills have yet to be adequately surveyed.

######## Remarks.

Distinguished from congeners by its arrangement of radial ribs, taller spire and the position of the pneumatophore.

####### Chamalycaeus microconus

Taxon classificationAnimaliaArchitaenioglossaCyclophoridae

(von Möllendorff, 1886)

Figure 5E

######## Materials examined.

Prk 55 G. Pondok: BOR/MOL 11617, BOR/MOL 11485.

######## Distribution.

Known only from Bukit Pondok and Temengor, Perak only (Maassen 2001).

######## Remarks.

Distinguished from all congeners by its tall spire, conical shell, penultimate whorl of constant width and the cross-hatching of radial and spiral ridges in post-apical whorls.

####### Chamalycaeus microdiscus

Taxon classificationAnimaliaArchitaenioglossaCyclophoridae

(von Möllendorff, 1886)

Figure 5F

######## Materials examined.

Prk 23 G. Rapat: BOR/MOL 10206.Prk 01 G. Tempurung: BOR/MOL 11236, BOR/MOL 11420.

######## Distribution.

Known from Bukit Pondok (Maassen 2001) and Kinta Valley, Perak only.

######## Remarks.

Shell small. Distinguished from congeners by its elongated shell at apical view, position of the pneumatophore and prominent spiral ribs at all whorls. New record for Kinta Valley.

####### Chamalycaeus oligopleuris

Taxon classificationAnimaliaArchitaenioglossaCyclophoridae

(von Möllendorff, 1886)

Figure 6A

######## Materials examined.

Prk 55 G. Pondok: BOR/MOL 11616.

######## Distribution.

Known from Gunung Pondok only.

######## Remarks.

Shell shape similar to *Chamalycaeus diplochilus* but differ by being smaller and have more prominent, wavy radial ribs.

**Figure 6.**
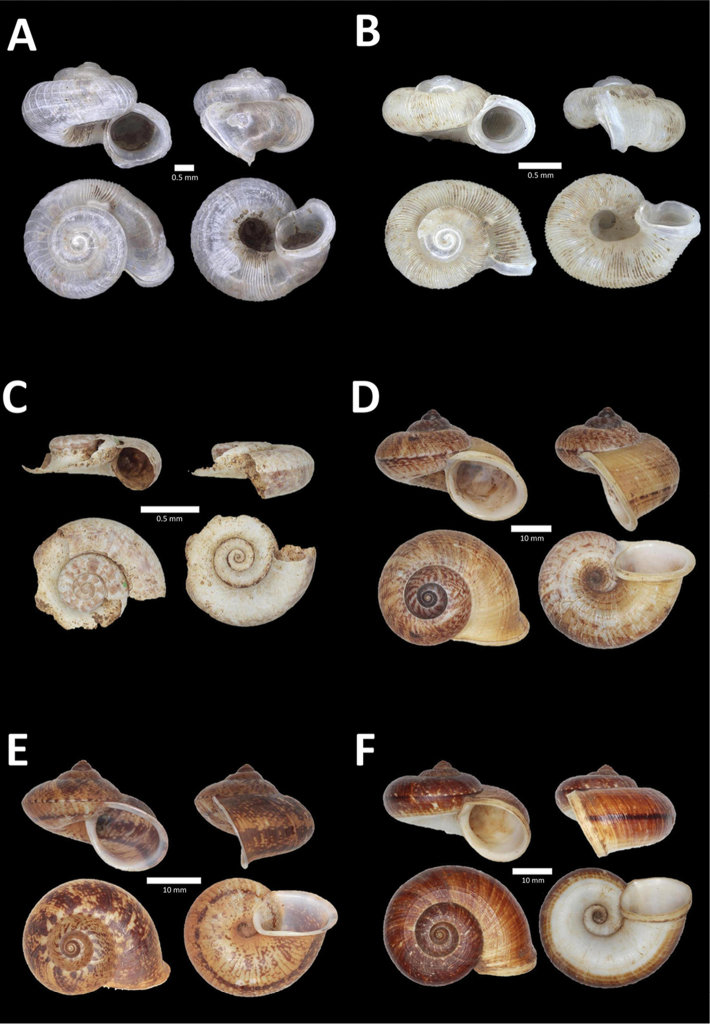
**A** *Chamalycaeus oligopleuris* (von Möllendorff, 1886) BOR/MOL 11616. Perak, Ipoh, Gunung Pondok **B** *Chamalycaeus parvulus* (von Möllendorff, 1886) BOR/MOL 11615. Perak, Ipoh, Gunung Pondok **C** *Crossopoma albersi* (Pfeiffer, 1847) BOR/MOL 9134. Perak, Ipoh, Gunung Kanthan Plot 2 **D** *Cyclophorus malayanus* (Benson, 1852a) BOR/MOL 9578. Perak, Ipoh, Batu Kebelah Plot 3 **E** *Cyclophorus perdix perdix* (Broderip & Sowerby, 1829) BOR/MOL 8274. Perak, Ipoh, Iron hill summit **F** *Cyclophorus semisulcatus* (Sowerby, 1843) BOR/MOL 9577. Perak, Ipoh, Batu Kebelah Plot 3.

####### Chamalycaeus parvulus

Taxon classificationAnimaliaArchitaenioglossaCyclophoridae

(von Möllendorff, 1886)

Figure 6B

######## Materials examined.

mykarst-025: BOR/MOL 9431, BOR/MOL 9514. Prk 42 G. Bercham: BOR/MOL 9467. Prk 36 Gua Datok: BOR/MOL 10506. Prk 53 Hill KF: BOR/MOL 10781. Prk 55 G. Pondok: BOR/MOL 11615, BOR/MOL 11587.

######## Distribution.

Known from Bukit Pondok (Maassen 2001) and Kinta Valley, Perak only.

######## Remarks.

Shell similar size to *Chamalycaeus microdiscus* but whorls differ in being rounder and radial ribs denser and less pronounced. New record for Kinta Valley.

**Genus *Crossopoma* von Martens, 1891**

####### Crossopoma albersi

Taxon classificationAnimaliaArchitaenioglossaCyclophoridae

(Pfeiffer, 1847)

Figure 6C

######## Materials examined.

mykarst-027: BOR/MOL 9134.

######## Distribution.

In Peninsular Malaysia, known from Dindings (=Manjung) and Kinta Valley, Perak (Maassen 2001). Elsewhere, in Sumatra, Indonesia (van Benthem Jutting 1959).

######## Remarks.

Shell distinguished from all other discoid cyclophorids by the presence of a deep, tunnel-like sutural canal which begins as a tube-like peristomal folding at the suture part of the aperture.

###### Genus *Cyclophorus* de Montfort, 1810

####### Cyclophorus malayanus

Taxon classificationAnimaliaArchitaenioglossaCyclophoridae

(Benson, 1852a)

Figure 6D

######## Materials examined.

Prk 64 Bt Kepala Gajah: BOR/MOL 10120, BOR/MOL 10086. mykarst-184 Bat Cave: BOR/MOL 9763, BOR/MOL 9874, BOR/MOL 9794, BOR/MOL 9829. Prk 53 Hill KF: BOR/MOL 10713, BOR/MOL 10738, BOR/MOL 10655, BOR/MOL 10685. mykarst-025: BOR/MOL 9380, BOR/MOL 9404, BOR/MOL 9437, BOR/MOL 9499, BOR/MOL 12433. mykarst-027: BOR/MOL 9047, BOR/MOL 9105. Prk 47 Kanthan: BOR/MOL 9066, BOR/MOL 9140. Prk 42 G. Bercham: BOR/MOL 9210, BOR/MOL 10568, BOR/MOL 10612, BOR/MOL 10628. mykarst-185 Batu Kebelah: BOR/MOL 9523, BOR/MOL 9560, BOR/MOL 9569, BOR/MOL 9578. Prk 36 Gua Datok: BOR/MOL 10414, BOR/MOL 10440. Prk 01 G. Tempurung: BOR/MOL 11130, BOR/MOL 11209, BOR/MOL 11240, BOR/MOL 11407. Prk 55 G. Pondok: BOR/MOL 11500, BOR/MOL 11476. Prk 34 G. Tasek: BOR/MOL 11059, BOR/MOL 11170.

######## Distribution.

Known from Langkawi (Kedah), Penang, Kelantan and Perak only (Maassen 2001).

######## Remarks.

Distinguished from congeners by its large shell, indistinct sculpture, rounded periphery and more thickened, expanded peristome. Colour pattern highly variable.

####### Cyclophorus perdix perdix

Taxon classificationAnimaliaArchitaenioglossaCyclophoridae

(Broderip & Sowerby, 1829)

Figure 6E

######## Materials examined.

Prk 23 G. Rapat: BOR/MOL 10226, BOR/MOL 10201. Prk 36 Gua Datok: BOR/MOL 10496. Prk 01 G. Tempurung: BOR/MOL 11131, BOR/MOL 11241.

######## Distribution.

In Peninsular Malaysia, known from Pahang (Maassen 2001). Elsewhere, in Sumatra and Java, Indonesia (van Benthem Jutting 1941, 1959).

######## Remarks.

Distinguished from congeners by its smaller shell, indistinct sculpture, somewhat keeled periphery and fine, complex brown mottled pattern.

####### Cyclophorus semisulcatus

Taxon classificationAnimaliaArchitaenioglossaCyclophoridae

(Sowerby, 1843)

Figure 6F

######## Materials examined.

Prk 64 Bt Kepala Gajah: BOR/MOL 10119, BOR/MOL 10085, BOR/MOL 10132, BOR/MOL 10163. mykarst-027: BOR/MOL 9048. Prk 23 G. Rapat: BOR/MOL 10224, BOR/MOL 10200, BOR/MOL 10248. Prk 47 Kanthan: BOR/MOL 9139. mykarst-185 Batu Kebelah: BOR/MOL 9524, BOR/MOL 9559, BOR/MOL 9577. Prk 36 Gua Datok: BOR/MOL 10448. Prk 42 G. Bercham: BOR/MOL 10588. Prk 55 G. Pondok: BOR/MOL 11501, BOR/MOL 11533, BOR/MOL 11475.

######## Distribution.

In Peninsular Malaysia, known from Pahang, Kelantan, Selangor and Perak only (Maassen 2001).

######## Remarks.

Distinguished from congeners by its small to large shell, pronounced spiral ridges and somewhat keeled periphery. Colour pattern highly variable.

###### Genus *Lagocheilus* Blanford, 1864

####### Lagocheilus

Taxon classificationAnimaliaArchitaenioglossaCyclophoridae

‘kinta 1’

Figure 7A

######## Materials examined.

Prk 53 Hill KF: BOR/MOL 10721, BOR/MOL 10677. mykarst-184 Bat Cave: BOR/MOL 9771, BOR/MOL 9813. Prk 36 Gua Datok: BOR/MOL 10421, BOR/MOL 10449, BOR/MOL 10481, BOR/MOL 10500. Prk 42 G. Bercham: BOR/MOL 10598. Prk 01 G. Tempurung: BOR/MOL 11238, BOR/MOL 11394, BOR/MOL 11427.

######## Distribution.

Known from Kinta Valley only.

######## Remarks.

Shell small, conical. Spire tall. Rounded dorsal whorls with numerous spiral ridges. Distinguished from *Lagocheilus kobelti* by its circular and flat peristome.

**Figure 7.**
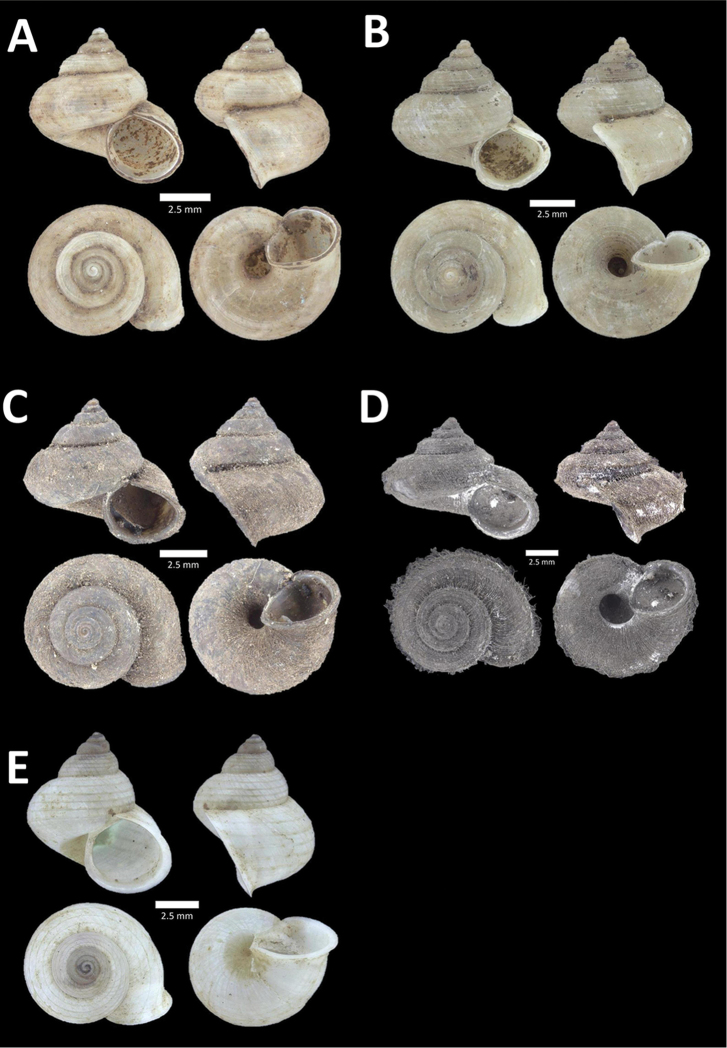
**A** *Lagochilus* ‘kinta 1’ BOR/MOL 10421. Perak, Ipoh, Gunung Datok Plot 2 **B** *Lagochilus kobelti* Sykes, 1903 BOR/MOL 10167. Perak, Ipoh, Gua Tok Giring Plot 4 **C** *Lagochilus* ‘pondok 1’ BOR/MOL 11576. Perak, Ipoh, Gunung Pondok, plot 6 **D** *Lagochilus townsendi* Crosse, 1879a BOR/MOL 9052. Perak, Ipoh, Gunung Kanthan Plot 3 **E** *Leptopoma aspirans* (Benson, 1856) BOR/MOL 8087. Perak, Ipoh, forested slope behind village at Gunung Pondok.

####### Lagocheilus kobelti

Taxon classificationAnimaliaArchitaenioglossaCyclophoridae

Sykes, 1903

Figure 7B

######## Materials examined.

Prk 64 Bt Kepala Gajah: BOR/MOL 10095, BOR/MOL 10138, BOR/MOL 10167. Prk 55 G. Pondok: BOR/MOL 11527, BOR/MOL 11482, BOR/MOL 11541, BOR/MOL 11575.

######## Distribution.

Known from Gunung Pondok only. Elsewhere, in Jalor (=Yala), Thailand (Maassen 2001).

######## Remarks.

Shell small, conical. Spire tall. Dorsal whorl angled at mid-distance between periphery and suture, in a region termed major spiral rib by Solem (1966). Differ from *Lagocheilus townsendi* in having a narrower penultimate whorl. New record for Perak.

####### Lagocheilus

Taxon classificationAnimaliaArchitaenioglossaCyclophoridae

‘pondok 1’

Figure 7C

######## Materials examined.

Prk 55 G. Pondok: BOR/MOL 11576.

######## Distribution.

Known from Gunung Pondok only, but surrounding hills have yet to be adequately surveyed.

######## Remarks.

Shell small, conical. Spire tall. Whorls convex, smooth with fine radial growth lines. Periphery keeled. Differ from congeners in the absence of spiral ridges.

####### Lagocheilus townsendi

Taxon classificationAnimaliaArchitaenioglossaCyclophoridae

Crosse, 1879a

Figure 7D

######## Materials examined.

Prk 47 Kanthan: BOR/MOL 9178, BOR/MOL 9052. mykarst-027: BOR/MOL 9095, BOR/MOL 9024.

######## Distribution.

Known from Perlis, Perak and Selangor only (Maassen 2001).

######## Remarks.

Shell small, conical. Spire tall. Similar to *Lagocheilus kobelti* in the presence of angled dorsal whorls at major spiral rib but penultimate whorl is more expanded and shell larger (Sykes 1903).

###### Genus *Leptopoma* Pfeiffer, 1847

####### Leptopoma aspirans

Taxon classificationAnimaliaArchitaenioglossaCyclophoridae

(Benson, 1856)

Figure 7E

######## Materials examined.

Prk 55 G. Pondok: BOR/MOL 11537.

######## Distribution.

In Peninsular Malaysia, known from Perlis and Perak (Maassen 2001). Elsewhere, in Tenasserim (=Tanintharyi), Myanmar and Jalor (=Yala), Thailand (Maassen 2001).

######## Remarks.

Shell small, conical. Dorsal whorls with widely spaced, indistinct brown spiral ridges. Distinguished from *Leptopoma perlucidum* (de Grateloup, 1840) by its taller spire relative to shell width. This is an arboreal species.

###### Genus *Opisthoporus* Pfeiffer, 1851

####### Opisthoporus penangensis

Taxon classificationAnimaliaArchitaenioglossaCyclophoridae

(Stoliczka, 1872)

Figure 8A

######## Materials examined.

Prk 64 Bt Kepala Gajah: BOR/MOL 10101, BOR/MOL 10127, BOR/MOL 10134, BOR/MOL 10173. Prk 55 G. Pondok: BOR/MOL 11505, BOR/MOL 11478, BOR/MOL 11535.

######## Distribution.

In Peninsular Malaysia, known from Penang, Perak and Kelantan (Maassen 2001). Elsewhere, in Pattani, Thailand (Maassen 2001).

######## Remarks.

Distinguished from *Opisthoporus rostellatus* (Pfeiffer, 1851) by the pneumatophore being nearer to the aperture and the absence of a wing-like sutural extension at the peristome.

**Figure 8.**
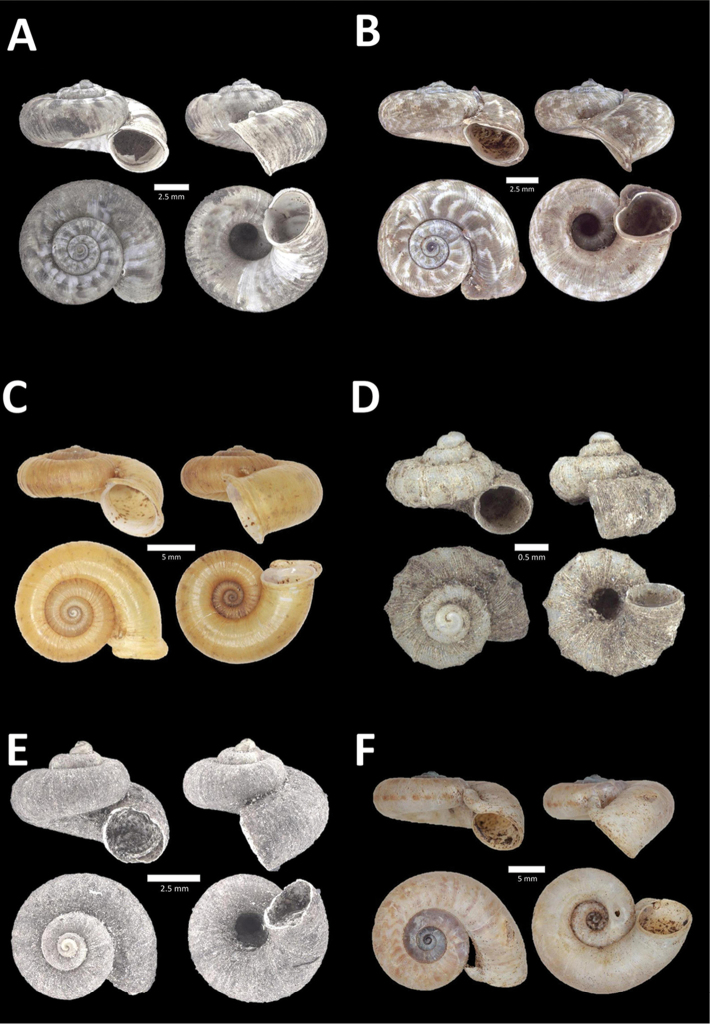
**A** *Opisthoporus penangensis* (Stoliczka, 1872) BOR/MOL 10101. Perak, Ipoh, Gua Tok Giring Plot 1 **B** *Opisthoporus rostellatus* (Pfeiffer, 1851) BOR/MOL 11410. Perak, Ipoh, Gunung Tempurung Plot 4 **C** *Opisthoporus solutus* (Stoliczka, 1872) BOR/MOL 11534. Perak, Ipoh, Gunung Pondok, plot 4 **D** *Platyraphe* ‘BatuKebelah 1’ BOR/MOL 10187. Perak, Ipoh, Gua Tok Giring Plot 4 **E** *Platyraphe lowi* (de Morgan, 1885a) BOR/MOL 9407. Perak, Ipoh, Mykarst-025 Plot 2 **F** *Rhiostoma jousseaumei* de Morgan, 1885b BOR/MOL 10225. Perak, Ipoh, Gunung Rapat Plot C6.

####### Opisthoporus rostellatus

Taxon classificationAnimaliaArchitaenioglossaCyclophoridae

(Pfeiffer, 1851)

Figure 8B

######## Materials examined.

mykarst-184 Bat Cave: BOR/MOL 9834. Prk 36 Gua Datok: BOR/MOL 10416, BOR/MOL 10444, BOR/MOL 10475, BOR/MOL 10488. Prk 42 G. Bercham: BOR/MOL 10595. Prk 01 G. Tempurung: BOR/MOL 11132, BOR/MOL 11212, BOR/MOL 11410.

######## Distribution.

In Peninsular Malaysia, known from Perak (Maassen 2001). Elsewhere, in Singapore (Maassen 2001).

######## Remarks.

Distinguished from *Opisthoporus penangensis* (Stoliczka, 1872) by the pneumatophore being longer and further from the aperture and the presence of a wing-like sutural extension at the peristome.

####### Opisthoporus solutus

Taxon classificationAnimaliaArchitaenioglossaCyclophoridae

(Stoliczka, 1872)

Figure 8C

######## Materials examined.

mykarst-184 Bat Cave: BOR/MOL 9892, BOR/MOL 9810. Prk 36 Gua Datok: BOR/MOL 10065, BOR/MOL 10473, BOR/MOL 10489. Prk 64 Bt Kepala Gajah: BOR/MOL 10107, BOR/MOL 10133, BOR/MOL 10164. Prk 55 G. Pondok: BOR/MOL 11504, BOR/MOL 11534, BOR/MOL 11477, BOR/MOL 11552, BOR/MOL 11566, BOR/MOL 11577.

######## Distribution.

Known from Penang and Perak only (Maassen 2001).

######## Remarks.

Differ from sympatric congeners by its patternless white shell and the yellow periostracum with equidistant spiral periostracal ridges.

###### Genus *Platyraphe* von Möllendorff, 1890

####### Platyraphe

Taxon classificationAnimaliaArchitaenioglossaCyclophoridae

‘batukebelah 1’

Figure 8D

######## Materials examined.

mykarst-185 Batu Kebelah: BOR/MOL 9752. Prk 64 Bt Kepala Gajah: BOR/MOL 10097, BOR/MOL 10146, BOR/MOL 10187.

######## Distribution.

Known from Batu Kebelah and Bukit Kepala Gajah, Perak only, but surrounding hills have yet to be adequately surveyed.

######## Remarks.

Very small shell at maturity compared to all congeners. Shell discoid. Medium spire. Fine spiral ridges and radial ridges interspersed with major radial ridges at regular intervals. Often encrusted with mud and plant debris.

####### Platyraphe lowi

Taxon classificationAnimaliaArchitaenioglossaCyclophoridae

(de Morgan, 1885a)

Figure 8E

######## Materials examined.

mykarst-184 Bat Cave: BOR/MOL 9891. Prk 53 Hill KF: BOR/MOL 10711, BOR/MOL 10742, BOR/MOL 10661, BOR/MOL 10691. mykarst-025: BOR/MOL 9385, BOR/MOL 9407, BOR/MOL 9484, BOR/MOL 9506. Prk 47 Kanthan: BOR/MOL 9058, BOR/MOL 9136. mykarst-027: BOR/MOL 9094, BOR/MOL 9012. Prk 42 G. Bercham: BOR/MOL 9459, BOR/MOL 9462, BOR/MOL 9222, BOR/MOL 9233, BOR/MOL 10574, BOR/MOL 10592, BOR/MOL 10613, BOR/MOL 10624. Prk 23 G. Rapat: BOR/MOL 10207, BOR/MOL 10052, BOR/MOL 10238, BOR/MOL 10249. mykarst-185 Batu Kebelah: BOR/MOL 9530, BOR/MOL 9563, BOR/MOL 9751. Prk 64 Bt Kepala Gajah: BOR/MOL 10106, BOR/MOL 10126, BOR/MOL 10135, BOR/MOL 10172. Prk 36 Gua Datok: BOR/MOL 10420, BOR/MOL 10445, BOR/MOL 10476, BOR/MOL 10490. Prk 34 G. Tasek: BOR/MOL 10787, BOR/MOL 11030, BOR/MOL 11064. Prk 01 G. Tempurung: BOR/MOL 11140, BOR/MOL 11215, BOR/MOL 11244, BOR/MOL 11411. Prk 55 G. Pondok: BOR/MOL 11502, BOR/MOL 11459, BOR/MOL 11536, BOR/MOL 11578.

######## Distribution.

In Peninsular Malaysia, found in Perlis, Perak and Selangor (Maassen 2001). Elsewhere, in Pattani, Thailand (Maassen 2001).

######## Remarks.

Distinct from *Platyraphe* ‘batukebelah 1’ in being much larger and has fine radial growth lines. Shell discoid. Low to medium spire. Often encrusted with solidified mud in a consistent, angular fashion when alive.

###### Genus *Rhiostoma* Benson, 1860

####### Rhiostoma jousseaumei

Taxon classificationAnimaliaArchitaenioglossaCyclophoridae

de Morgan, 1885b

Figure 8F

######## Materials examined.

Prk 47 Kanthan: BOR/MOL 9179, BOR/MOL 9069, BOR/MOL 9059, BOR/MOL 9135.mykarst-027: BOR/MOL 9088, BOR/MOL 9109, BOR/MOL 9029, BOR/MOL 9031.Prk 53 Hill KF: BOR/MOL 10720, BOR/MOL 10662.mykarst-025: BOR/MOL 9386, BOR/MOL 9418, BOR/MOL 9490, BOR/MOL 9507, BOR/MOL 12423.Prk 42 G. Bercham: BOR/MOL 9470, BOR/MOL 9482, BOR/MOL 9208, BOR/MOL 9227, BOR/MOL 9229, BOR/MOL 9230, BOR/MOL 9232, BOR/MOL 10572, BOR/MOL 10594, BOR/MOL 10614, BOR/MOL 10625, BOR/MOL 10639.Prk 23 G. Rapat: BOR/MOL 10225, BOR/MOL 10055, BOR/MOL 10203, BOR/MOL 10250, BOR/MOL 10270. Prk 36 Gua Datok: BOR/MOL 10417, BOR/MOL 10442, BOR/MOL 10472, BOR/MOL 10491.Prk 34 G. Tasek: BOR/MOL 10786, BOR/MOL 11026, BOR/MOL 11010, BOR/MOL 11040, BOR/MOL 11053.

######## Distribution.

Restricted to Kinta Valley.

######## Remarks.

Shell small to medium-sized. Final whorl occasionally detached, with large upturned pneumatophore formed through peristomal folding. Shell patterns vary from dense zigzags and peripheral band over brown whorls to fugitive brown zigzags over white or purple whorls. *Rhiostoma macalpinewoodsi* (Laidlaw 1939) is synonymous with *Rhiostoma jousseaumei*, as intermediates were found in our study sites.

##### Family Diplommatinidae Pfeiffer, 1856

###### Genus *Arinia* Adams & Adams, 1856

####### Subgenus Notharinia Vermeulen, Phung & Truong, 2007

######## Arinia (Notharinia) micro

Taxon classificationAnimaliaMesogastropodaDiplommatinidae

Marzuki & Foon, 2016

Figure 9A

######### Materials examined.

Prk 23 G. Rapat: BOR/MOL 10288.

######### Distribution.

Known from Gunung Rapat only, but surrounding hills have yet to be adequately surveyed.

######### Remarks.

Shell dextral, very small. Cylindrical, tall spire. Radial ribs dense, spiral ridges very fine. Differ from Arinia (Notharinia) ‘tasek 1’ in the final whorl being less detached.

**Figure 9.**
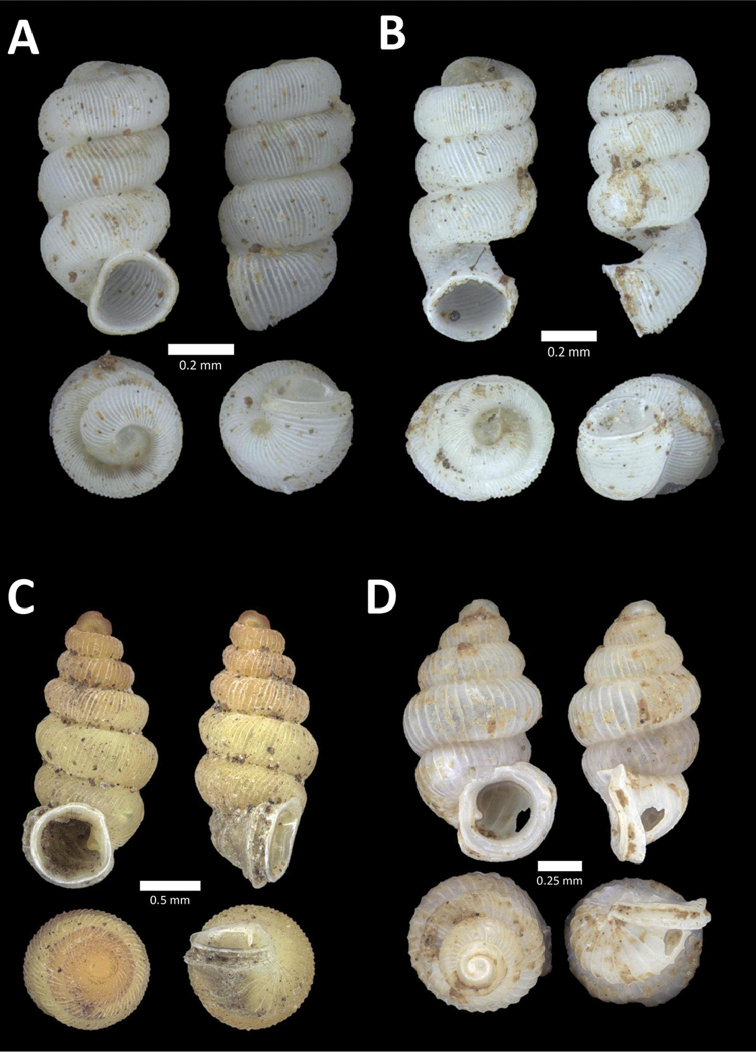
**A** Arinia (Notharinia) micro Marzuki & Foon, 2016 BOR/MOL 10288. Perak, Ipoh, Gunung Rapat **B** Arinia (Notharinia) ‘tasek 1’ BOR/MOL 11193. Perak, Ipoh, Gunung Tasek Plot 2 **C** Diplommatina cf. diminuta BOR/MOL 9023. Perak, Ipoh, Gunung Kanthan Plot 1.D. *Diplommatina crosseana* Godwin-Austen & Nevill, 1879 BOR/MOL 9021. Perak, Ipoh, Gunung Kanthan.

######## Arinia (Notharinia)

Taxon classificationAnimaliaMesogastropodaDiplommatinidae

‘tasek 1’

Figure 9B

######### Materials examined.

Prk 34 G. Tasek: BOR/MOL 11166, BOR/MOL 11193.

######### Distribution.

Known from Gunung Tasek only, but surrounding hills have yet to be adequately surveyed.

######### Remarks.

Shell dextral, very small. Cylindrical, tall spire. Radial ribs dense, spiral ridges very fine. Differ from Arinia (Notharinia) micro in that the final whorl is completely detached and coiled downwards.

###### Genus Diplommatina Benson, 1849

####### Diplommatina cf. diminuta

Taxon classificationAnimaliaMesogastropodaDiplommatinidae

Figure 9C

######## Materials examined.

mykarst-027: BOR/MOL 9113, BOR/MOL 9023.

######## Distribution.

Known from mykarst-027 only, but surrounding hills have yet to be adequately surveyed.

######## Remarks.

Shell sinistral. Shell very similar to *Diplommatina diminuta* von Möllendorff, 1891 but differ in having denser radial ribs.

####### Diplommatina crosseana

Taxon classificationAnimaliaMesogastropodaDiplommatinidae

Godwin-Austen & Nevill, 1879

Figure 9D

######## Materials examined.

Prk 47 Kanthan: BOR/MOL 9087. mykarst-184 Bat Cave: BOR/MOL 9850. mykarst-027: BOR/MOL 9021.

######## Distribution.

Known from upper Kinta Valley and Perak River valley, Perak only (Maassen 2001).

######## Remarks.

Shell dextral. Smallest shell of all congeners. Also differ from congeners by the peristome that is reflected and thickened multiple times, sharply angular at the base of the columella and slightly angular at the upper outer lip (Laidlaw 1949). Davison (1991) considers *D. crosseana* as possibly related to *Diplommatina maduana* Laidlaw, 1949.

####### Diplommatina lenggongensis

Taxon classificationAnimaliaMesogastropodaDiplommatinidae

Tomlin, 1941

Figure 10A

######## Materials examined.

Prk 64 Bt Kepala Gajah: BOR/MOL 10111, BOR/MOL 10153, BOR/MOL 10191.

######## Distribution.

Restricted to karsts in Lenggong, Perak only (Maassen 2001).

######## Remarks.

Shell sinistral. Shell large among congeners. The characters distinguishing *Diplommatina lenggongensis* from congeners: Spire very tall, slender. Suture deep. Radial ribs pronounced, extending outwards as a triangular protrusion along the periphery.

**Figure 10.**
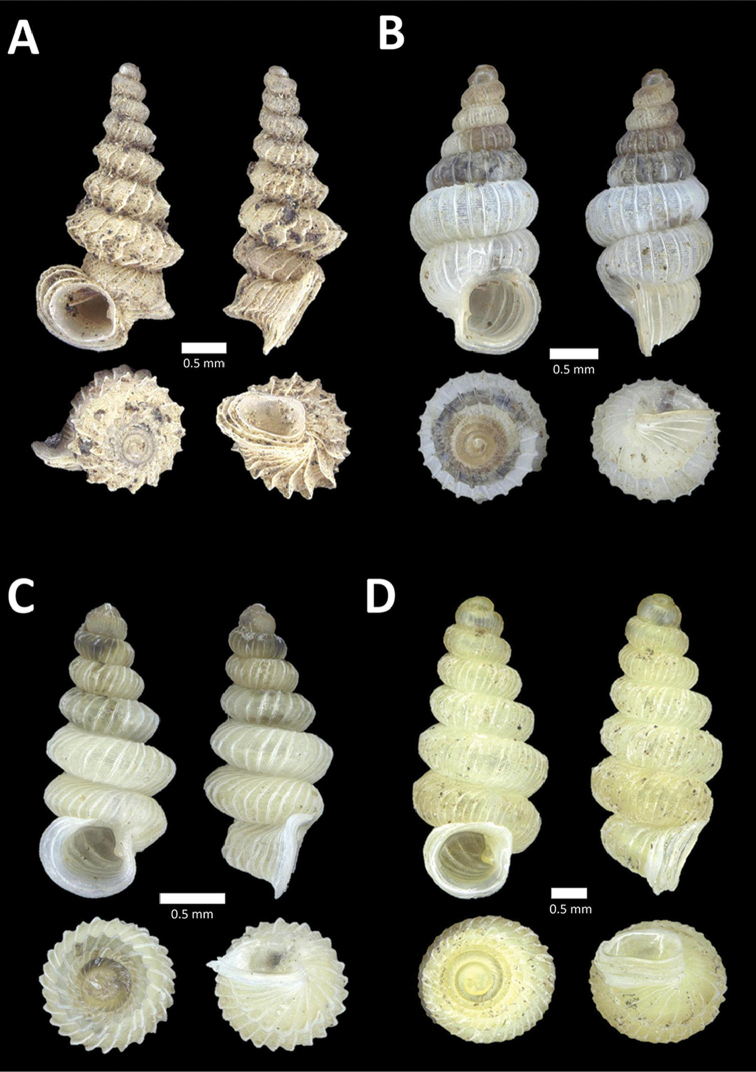
**A** *Diplommatina lenggongensis* Laidlaw, 1941 BOR/MOL 10111. Perak, Ipoh, Gua Tok Giring Plot 1 **B** *Diplommatina nevilli* Crosse, 1879a BOR/MOL 9532. Perak, Ipoh, Batu Kebelah Plot 1 **C** *Diplommatina parabates* Laidlaw, 1949 BOR/MOL 11556. Perak, Ipoh, Gunung Pondok, plot 4 **D** *Diplommatina sinistra* Tomlin, 1938 BOR/MOL 9074. Perak, Ipoh, Gunung Kanthan Plot 3.

####### Diplommatina nevilli

Taxon classificationAnimaliaMesogastropodaDiplommatinidae

Crosse, 1879a

Figure 10B

######## Materials examined.

Prk 53 Hill KF: BOR/MOL 10698, BOR/MOL 10749, BOR/MOL 10732, BOR/MOL 10680, BOR/MOL 10783. mykarst-184 Bat Cave: BOR/MOL 9884, BOR/MOL 9783, BOR/MOL 9820, BOR/MOL 9848. Prk 64 Bt Kepala Gajah: BOR/MOL 10112, BOR/MOL 10113, BOR/MOL 10123, BOR/MOL 10155, BOR/MOL 10156, BOR/MOL 10190. mykarst-025: BOR/MOL 9389, BOR/MOL 9422, BOR/MOL 9423, BOR/MOL 9441, BOR/MOL 9442, BOR/MOL 9511. Prk 47 Kanthan: BOR/MOL 9062, BOR/MOL 9170. mykarst-027: BOR/MOL 9114, BOR/MOL 9020. Prk 42 G. Bercham: BOR/MOL 9481, BOR/MOL 9218, BOR/MOL 9235, BOR/MOL 10576, BOR/MOL 10609, BOR/MOL 10617, BOR/MOL 10640. mykarst-185 Batu Kebelah: BOR/MOL 9532, BOR/MOL 9744. Prk 23 G. Rapat: BOR/MOL 10033. Prk 36 Gua Datok: BOR/MOL 10068, BOR/MOL 10432, BOR/MOL 10463, BOR/MOL 10470. Prk 01 G. Tempurung: BOR/MOL 11154, BOR/MOL 11235, BOR/MOL 11402, BOR/MOL 11403, BOR/MOL 11429. Prk 34 G. Tasek: BOR/MOL 11159, BOR/MOL 11014, BOR/MOL 11015, BOR/MOL 11048, BOR/MOL 11049, BOR/MOL 11189. Prk 55 G. Pondok: BOR/MOL 11494, BOR/MOL 11495, BOR/MOL 11514, BOR/MOL 11584, BOR/MOL 11588, BOR/MOL 11555.

######## Distribution.

Found across Peninsular Malaysia (Maassen 2001). Elsewhere, in Singapore, Thailand and Indonesia (Ho 1995, Panha and Burch 2005, van Benthem Jutting 1941, 1959).

######## Remarks.

Shell dextral, rarely sinistral. Shell vary greatly in height, rib density, number of whorls (7 to 8) and aperture thickness within and between populations. After examining large samples from our study sites, we concur with Tweedie in Laidlaw (1949) that it is not possible to separate *Diplommatina canaliculata* von Möllendorff, 1886 from *Diplommatina nevilli*. The specimens labelled as *Diplommatina mirabilis* in Davison (1991) are actually *Diplommatina nevilli*.

####### Diplommatina parabates

Taxon classificationAnimaliaMesogastropodaDiplommatinidae

Laidlaw, 1949

Figure 10C

######## Materials examined.

Prk 64 Bt Kepala Gajah: BOR/MOL 10152. Prk 55 G. Pondok: BOR/MOL 11492, BOR/MOL 11515, BOR/MOL 11517, BOR/MOL 11585, BOR/MOL 11556.

######## Distribution.

Known from Lenggong and Gunung Pondok, Perak only.

######## Remarks.

Shell sinistral. Shell distinctly different from sympatric congeners by the rounder periphery, less pronounced spiral ribs and shallower suture. New record for Gunung Pondok.

####### Diplommatina sinistra

Taxon classificationAnimaliaMesogastropodaDiplommatinidae

Tomlin, 1938

Figure 10D

######## Materials examined.

Prk 47 Kanthan: BOR/MOL 9074, BOR/MOL 9149. mykarst-184 Bat Cave: BOR/MOL 9893, BOR/MOL 9785, BOR/MOL 9847. Prk 42 G. Bercham: BOR/MOL 9456, BOR/MOL 10653. mykarst-185 Batu Kebelah: BOR/MOL 9754. Prk 34 G. Tasek: BOR/MOL 11018.

######## Distribution.

Restricted to Upper Kinta Valley, Perak only.

######## Remarks.

Shell sinistral. Differ from sympatric *Diplommatina diminuta* in having shorter spire, more widely spaced radial ribs and more slender shell. Previously known solely from Sungai Siput North (Maassen 2001).

####### Diplommatina streptophora

Taxon classificationAnimaliaMesogastropodaDiplommatinidae

Laidlaw, 1949

Figure 11A

######## Materials examined.

mykarst-184 Bat Cave: BOR/MOL 9882, BOR/MOL 9784. Prk 47 Kanthan: BOR/MOL 9061, BOR/MOL 9166. Prk 23 G. Rapat: BOR/MOL 10216, BOR/MOL 10037, BOR/MOL 10246, BOR/MOL 10263. mykarst-027: BOR/MOL 9120, BOR/MOL 9025. mykarst-185 Batu Kebelah: BOR/MOL 9533, BOR/MOL 9746. Prk 36 Gua Datok: BOR/MOL 10069, BOR/MOL 10468. Prk 64 Bt Kepala Gajah: BOR/MOL 10158.

######## Distribution.

Found throughout Perak only (Maassen 2001).

######## Remarks.

Shell dextral, rarely sinistral. Distinct from all sympatric congeners by its rather distended ultimate whorl and reflected aperture which extends to the right of the body whorl. Sinistral specimens of this species were labelled as *Diplommatina* sp. in Davison (1991).

**Figure 11.**
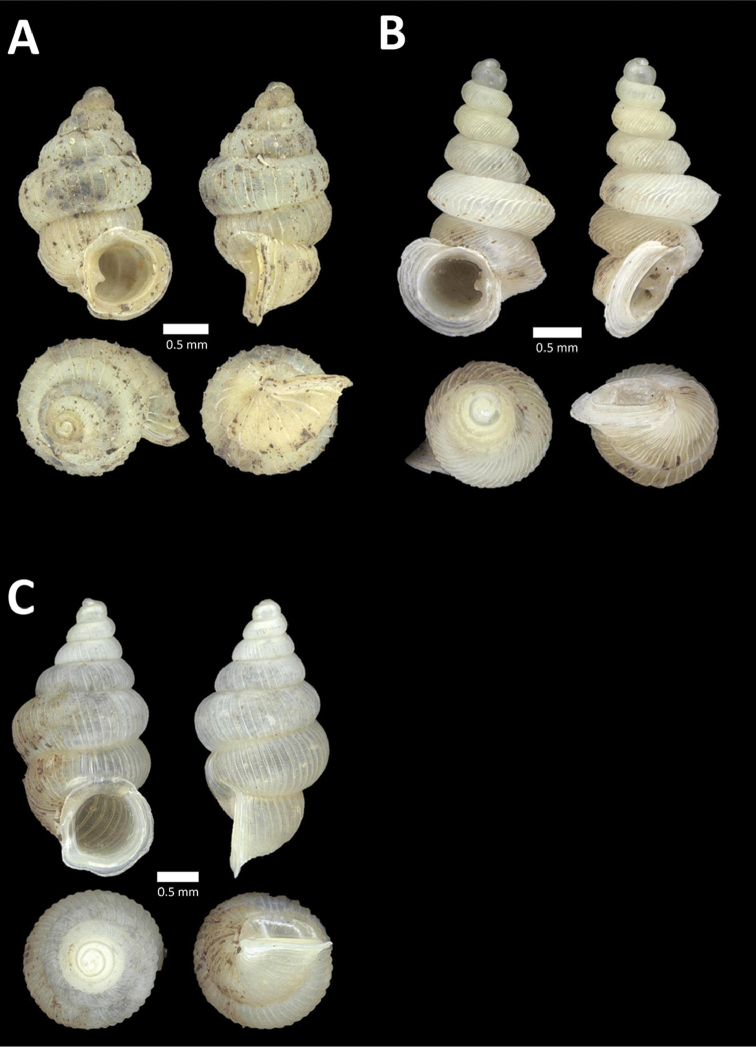
**A** *Diplommatina streptophora* Laidlaw, 1949 BOR/MOL 9061. Perak, Ipoh, Gunung Kanthan Plot 3 **B** *Diplommatina superba superba* Godwin-Austen & Nevill, 1879 BOR/MOL 11557. Perak, Ipoh, Gunung Pondok **C** *Diplommatina ventriculus* (von Möllendorff, 1891) BOR/MOL 9027. Perak, Ipoh, Gunung Kanthan.

####### Diplommatina superba superba

Taxon classificationAnimaliaMesogastropodaDiplommatinidae

Godwin-Austen & Nevill, 1879

Figure 11B

######## Materials examined.

Prk 47 Kanthan: BOR/MOL 9181, BOR/MOL 9063. Prk 53 Hill KF: BOR/MOL 10709, BOR/MOL 10733, BOR/MOL 10747, BOR/MOL 10682. Prk 23 G. Rapat: BOR/MOL 10217, BOR/MOL 10039, BOR/MOL 10247, BOR/MOL 10272. mykarst-027: BOR/MOL 9123, BOR/MOL 9017. mykarst-185 Batu Kebelah: BOR/MOL 9745. Prk 64 Bt Kepala Gajah: BOR/MOL 10154. Prk 36 Gua Datok: BOR/MOL 10435, BOR/MOL 10464. Prk 42 G. Bercham: BOR/MOL 10611. Prk 01 G. Tempurung: BOR/MOL 11153, BOR/MOL 11233, BOR/MOL 11406, BOR/MOL 11428. Prk 34 G. Tasek: BOR/MOL 11165. Prk 55 G. Pondok: BOR/MOL 11516, BOR/MOL 11586, BOR/MOL 11557.

######## Distribution.

Known from Gunung Pondok and Kinta Valley only (Maassen 2001).

######## Remarks.

Shell sinistral. Shell vary greatly in height and width, rib density, rib height and aperture thickness within and between populations. Consistent features are keeled periphery and whorl shape. After examining large samples from our study sites, we conclude that it is not possible to separate *Diplommatina superba superba* from *Diplommatina superba brevior* Laidlaw, 1949.

####### Diplommatina ventriculus

Taxon classificationAnimaliaMesogastropodaDiplommatinidae

(von Möllendorff, 1891)

Figure 11C

######## Materials examined.

Prk 53 Hill KF: BOR/MOL 10703, BOR/MOL 10745, BOR/MOL 10725, BOR/MOL 10681. mykarst-184 Bat Cave: BOR/MOL 9883, BOR/MOL 9821. Prk 47 Kanthan: BOR/MOL 9180, BOR/MOL 9086. Prk 23 G. Rapat: BOR/MOL 10219, BOR/MOL 10265. mykarst-027: BOR/MOL 9117, BOR/MOL 9018, BOR/MOL 9027. Prk 64 Bt Kepala Gajah: BOR/MOL 10157. Prk 55 G. Pondok: BOR/MOL 11493, BOR/MOL 11518, BOR/MOL 11554, BOR/MOL 11582. Prk 34 G. Tasek: BOR/MOL 11191. Prk 01 G. Tempurung: BOR/MOL 11234, BOR/MOL 11404.

######## Distribution.

Found throughout Peninsular Malaysia (Maassen 2001). Elsewhere, in Sumatra, Indonesia (van Benthem Jutting 1959).

######## Remarks.

Shell dextral, rarely sinistral. Distinguished from other sympatric dextral congeners by its large shell, more globular whorls and weak columellar tooth.

###### Genus *Opisthostoma* Blanford & Blanford, 1860

####### Opisthostoma castor

Taxon classificationAnimaliaMesogastropodaDiplommatinidae

van Benthem Jutting, 1952

Figure 12A

######## Materials examined.

Prk 64 Bt Kepala Gajah: BOR/MOL 10115, BOR/MOL 10147, BOR/MOL 10193.

######## Distribution.

Restricted to Lenggong, Perak only (Maassen 2001).

######## Remarks.

See the Remarks section of *Opisthostoma fallax*.

**Figure 12.**
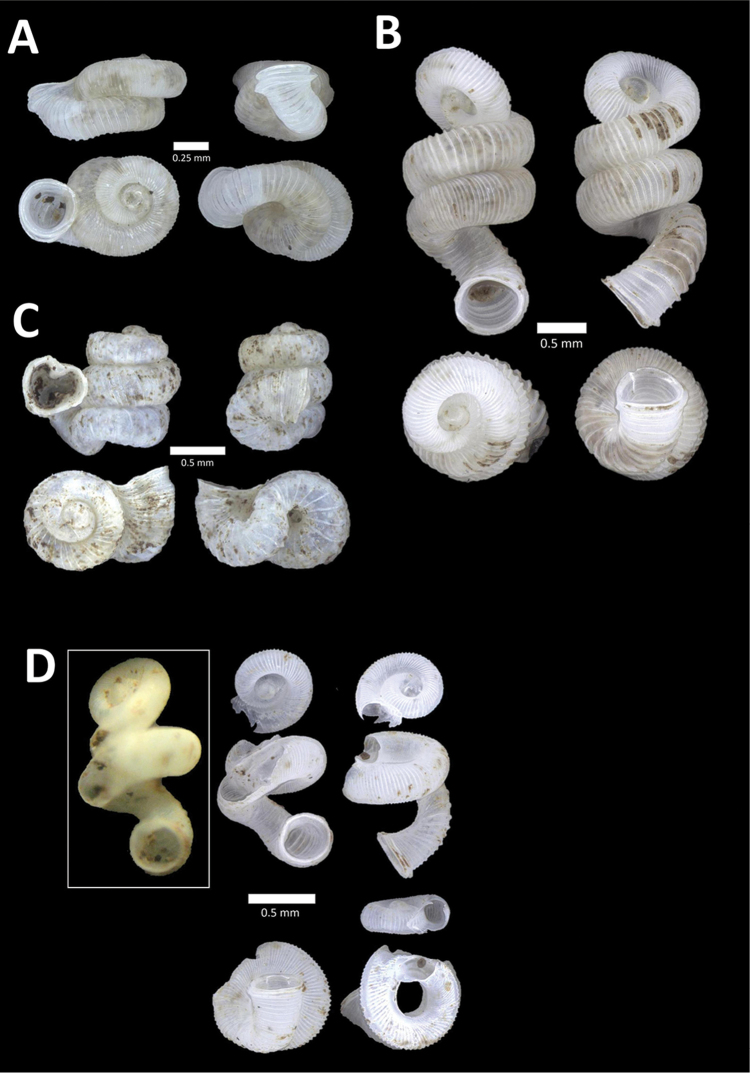
**A** *Opisthostoma castor* van Benthem Jutting, 1952 BOR/MOL 10115. Perak, Ipoh, Gua Tok Giring Plot 1 **B** Opisthostoma cf. gittenbergeri BOR/MOL 9151. Perak, Ipoh, Gunung Kanthan Plot 4 **C** Opisthostoma cf. subconicum BOR/MOL 9183. Perak, Ipoh, Gunung Kanthan Plot 4 **D** Opisthostoma cf. vermiculum BOR/MOL 11400. Perak, Ipoh, Gunung Tempurung Plot 2.

####### Opisthostoma cf. gittenbergeri

Taxon classificationAnimaliaMesogastropodaDiplommatinidae

Figure 12B

######## Materials examined.

Prk 47 Kanthan: BOR/MOL 9151, BOR/MOL 12496.

######## Distribution.

Known from Gunung Kanthan only, but surrounding hills have yet to be adequately surveyed.

######## Remarks.

Similar to *Opisthostoma gittenbergeri* in the manner of coiling for the penultimate and ultimate whorls. Differ from *O. gittenbergeri* in wider whorl coiling and position of apical and antepenultimate whorls. Radial rib density is variable.

####### Opisthostoma cf. subconicum

Taxon classificationAnimaliaMesogastropodaDiplommatinidae

Figure 12C

######## Materials examined.

Prk 47 Kanthan: BOR/MOL 9183.

######## Distribution.

Known from Gunung Kanthan only, but surrounding hills have yet to be adequately surveyed.

######## Remarks.

Similar to *Opisthostoma subconicum* Vermeulen, 1994, in shell shape, differ in having a more depressed apical whorl, less expanded peristome, more pronounced parietalis at the lower aperture and tooth at the upper aperture.

####### Opisthostoma cf. vermiculum

Taxon classificationAnimaliaMesogastropodaDiplommatinidae

Figure 12D

######## Materials examined.

Prk 01 G. Tempurung: BOR/MOL 11400.

######## Distribution.

Known from Gunung Tempurung only, but surrounding hills have yet to be adequately surveyed.

######## Remarks.

Similar to *Opisthostoma vermiculum* in the manner of coiling for the penultimate and ultimate whorls. Differ from *O. vermiculum* in the coiling axis of apical and antepenultimate whorls.

####### Opisthostoma fallax

Taxon classificationAnimaliaMesogastropodaDiplommatinidae

van Benthem Jutting, 1961a

Figure 13A

######## Materials examined.

mykarst-025: BOR/MOL 9420. mykarst-185 Batu Kebelah: BOR/MOL 9755, BOR/MOL 12519. Prk 01 G. Tempurung: BOR/MOL 11206, BOR/MOL 11231, BOR/MOL 12505. mykarst-184 Bat Cave: BOR/MOL 12499.

######## Distribution.

Restricted to Kinta Valley.

######## Remarks.

Similar to *Opisthostoma castor* in shell shape, differ in convexity of the whorl, larger umbilicus and the position of the ultimate whorl relative to the penultimate whorl (van Benthem Jutting 1961a).

**Figure 13.**
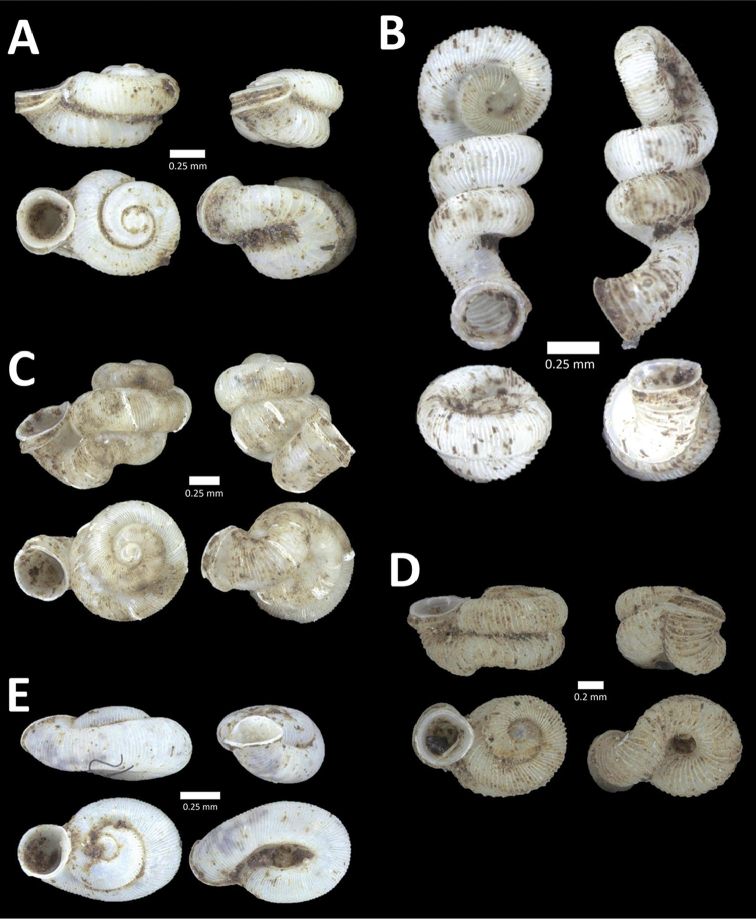
**A** *Opisthostoma fallax* van Benthem Jutting, 1961 BOR/MOL 9755. Perak, Ipoh, Batu Kebelah Plot 4 **B** *Opisthostoma gittenbergeri* Vermeulen & Clements, 2008 BOR/MOL 10452. Perak, Ipoh, Gunung Datok **C** *Opisthostoma* ‘Kanthan 1’ BOR/MOL 9182. Perak, Ipoh, Gunung Kanthan Plot 3 **D** *Opisthostoma megalomphalum* van Benthem Jutting, 1952 BOR/MOL 10271. Perak, Ipoh, Gunung Rapat Plot C7 **E** *Opisthostoma* ‘mykarst-025 1’ BOR/MOL 12506. Perak, Ipoh, Mykarst-025 Plot 4.

####### Opisthostoma gittenbergeri

Taxon classificationAnimaliaMesogastropodaDiplommatinidae

Vermeulen & Clements, 2008

Figure 13B

######## Materials examined.

Prk 36 Gua Datok: BOR/MOL 10452.Prk 42 G. Bercham: BOR/MOL 10604.

######## Distribution.

Known from Gua Datok and Gunung Bercham only, but surrounding hills have yet to be adequately surveyed.

######## Remarks.

Shell shape distinctive among congeners. Differ from Opisthostoma cf. gittenbergeri in whorl coiling being tighter and the position of apical and antepenultimate whorls.

####### Opisthostoma

Taxon classificationAnimaliaMesogastropodaDiplommatinidae

‘Kanthan 1’

Figure 13C

######## Materials examined.

Prk 47 Kanthan: BOR/MOL 9182, BOR/MOL 9070, BOR/MOL 9060, BOR/MOL 9158, BOR/MOL 9168.mykarst-027: BOR/MOL 9115, BOR/MOL 9026. Prk 34 G. Tasek: BOR/MOL 11162, BOR/MOL 11007, BOR/MOL 11050, BOR/MOL 11187, BOR/MOL 11192.

######## Distribution.

Known from Gunung Kanthan and Gunung Tasek only, but surrounding hills have yet to be adequately surveyed.

######## Remarks.

Spire height, radial rib density and whorl shape variable. Differ from Opisthostoma cf. subconicum in having rounder whorls, finer but distinct radial ribs and the direction the aperture is facing.

####### Opisthostoma megalomphalum

Taxon classificationAnimaliaMesogastropodaDiplommatinidae

van Benthem Jutting, 1952

Figure 13D

######## Materials examined.

Prk 23 G. Rapat: BOR/MOL 10215, BOR/MOL 10035, BOR/MOL 10245, BOR/MOL 10271.

######## Distribution.

Known from Kramat Pulai (van Benthem Jutting 1952) and Gunung Rapat only, but surrounding hills have yet to be adequately surveyed.

######## Remarks.

Differ from other flat-shelled congeners by the convexity of the whorl, larger umbilicus, the position of apical and antepenultimate whorls and the direction the aperture is facing (van Benthem Jutting 1961a).

####### Opisthostoma

Taxon classificationAnimaliaMesogastropodaDiplommatinidae

‘mykarst-025 1’

Figure 13E

######## Materials examined.

mykarst-025: BOR/MOL 9396, BOR/MOL 12506, BOR/MOL 12508, BOR/MOL 12520.

######## Distribution.

Known from mykarst-025 only, but surrounding hills have yet to be adequately surveyed.

######## Remarks.

Differ from other flat-shelled congeners by its much smaller shell and most distinctly, the coiling of the antepenultimate whorl.

####### Opisthostoma paulucciae

Taxon classificationAnimaliaMesogastropodaDiplommatinidae

(Crosse & Nevill, in Crosse 1879b)

Figure 14A

######## Materials examined.

Prk 53 Hill KF: BOR/MOL 10697, BOR/MOL 10724, BOR/MOL 10683. mykarst-027: BOR/MOL 9130, BOR/MOL 9019. mykarst-184 Bat Cave: BOR/MOL 9849. Prk 36 Gua Datok: BOR/MOL 10082, BOR/MOL 10462. Prk 23 G. Rapat: BOR/MOL 10244. Prk 42 G. Bercham: BOR/MOL 10610. Prk 55 G. Pondok: BOR/MOL 11519, BOR/MOL 11558, BOR/MOL 11581. Prk 34 G. Tasek: BOR/MOL 11051. Prk 01 G. Tempurung: BOR/MOL 11205, BOR/MOL 11232, BOR/MOL 11401, BOR/MOL 11405.

######## Distribution.

Known from Gunung Pondok and Kinta Valley, Perak only.

######## Remarks.

Variable in spire height and the position of the apical whorls. Most similar to *Opisthostoma* ‘Kanthan 1’ but differ in the direction the aperture is facing as well as the less dense and more pronounced radial ribs.

**Figure 14.**
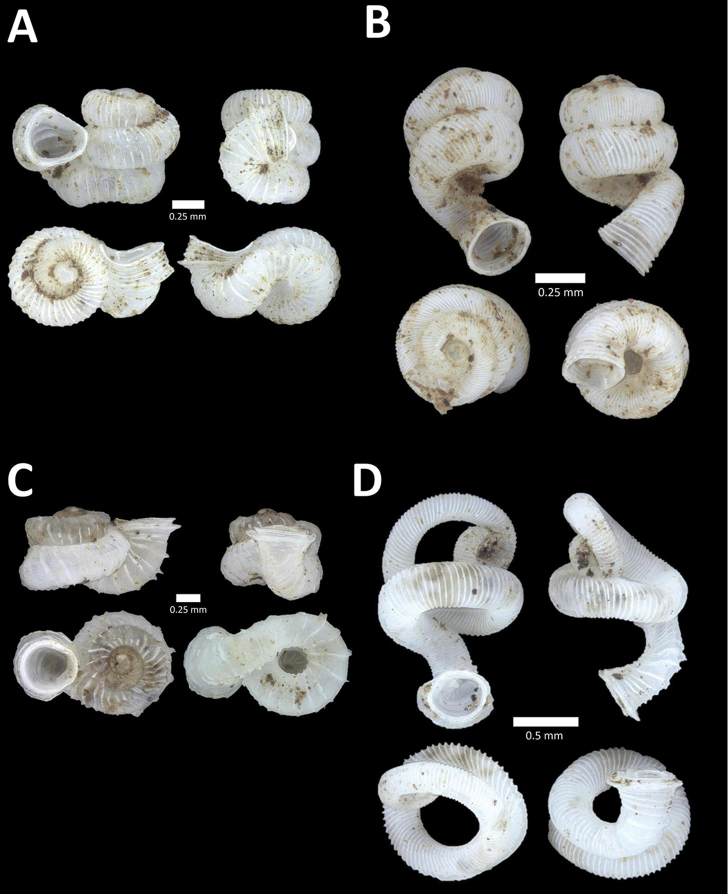
**A** *Opisthostoma paulucciae* ((Crosse and Nevill, in Crosse, 1879b)) BOR/MOL 9019. Perak, Ipoh, Gunung Kanthan **B** *Opisthostoma* ‘tempurung 1 detached’ BOR/MOL 12504. Perak, Ipoh, Gunung Tempurung Plot 4 **C** *Opisthostoma trapezium* van Benthem Jutting, 1952 BOR/MOL 9035. Perak, Ipoh, Gunung Kanthan **D** *Opisthostoma vermiculum* Clements and Vermeulen, in Clements et al., 2008 BOR/MOL 10242. Perak, Ipoh, Gunung Rapat.

####### Opisthostoma

Taxon classificationAnimaliaMesogastropodaDiplommatinidae

‘tempurung 1 detached’

Figure 14B

######## Materials examined.

Prk 01 G. Tempurung: BOR/MOL 12500, BOR/MOL 12504.

######## Distribution.

Known from Gunung Tempurung, but surrounding hills have yet to be adequately surveyed.

######## Remarks.

Distinct from congeners in shell shape. Radial ribbing dense. *Opisthostoma* ‘tempurung 1 detached’ has a detached ultimate whorl similar to *Notharinia* ‘tasek 1’ but differ in having larger and less number of whorls.

####### Opisthostoma trapezium

Taxon classificationAnimaliaMesogastropodaDiplommatinidae

van Benthem Jutting, 1952

Figure 14C

######## Materials examined.

Prk 47 Kanthan: BOR/MOL 9071, BOR/MOL 9164. mykarst-027: BOR/MOL 9035, BOR/MOL 9126. Prk 53 Hill KF: BOR/MOL 10780.mykarst-025: BOR/MOL 9397, BOR/MOL 9421, BOR/MOL 9489, BOR/MOL 9515.

######## Distribution.

Restricted to central and upper Kinta Valley only.

######## Remarks.

Distinguished from congeners by its box-like shell shape, pronounced but widely spaced radial ribs and the direction the aperture is facing is parallel to the coiling axis of the apical whorl. Previously known from Gunung Kanthan only (Maassen 2001).

####### Opisthostoma vermiculum

Taxon classificationAnimaliaMesogastropodaDiplommatinidae

Clements & Vermeulen, in Clements et al. 2008

Figure 14D

######## Materials examined.

Prk 23 G. Rapat: BOR/MOL 10214, BOR/MOL 10242, BOR/MOL 10267.

######## Distribution.

Known from Gunung Rapat only (Clements et al. 2008), but surrounding hills have yet to be adequately surveyed.

######## Remarks.

Distinguished from congeners by its shell shape and the presence of four coiling axes. Differ from Opisthostoma cf. vermiculum only in the coiling axis of the apical and antepenultimate whorls.

##### Family Pupinidae Pfeiffer, 1853a

###### Genus *Pollicaria* Gould, 1856

####### Pollicaria elephas

Taxon classificationAnimaliaMesogastropodaPupinidae

(de Morgan, 1885)

Figure 15A

######## Materials examined.

mykarst-027: BOR/MOL 9089, BOR/MOL 9030. Prk 53 Hill KF: BOR/MOL 10714, BOR/MOL 10741, BOR/MOL 10657, BOR/MOL 10684. mykarst-025: BOR/MOL 9402, BOR/MOL 9432, BOR/MOL 9443, BOR/MOL 9498. mykarst-185 Batu Kebelah: BOR/MOL 9567, BOR/MOL 9579. Prk 01 G. Tempurung: BOR/MOL 11138.

######## Distribution.

Known from Pluss (=Pelus) River Valley and Kinta Valley, Perak only (Maassen 2001).

######## Remarks.

Shell large. Operculum hard, calcified, multilamella. Distinct from any sympatric land snail species in the structure and coiling of the whorls.

**Figure 15.**
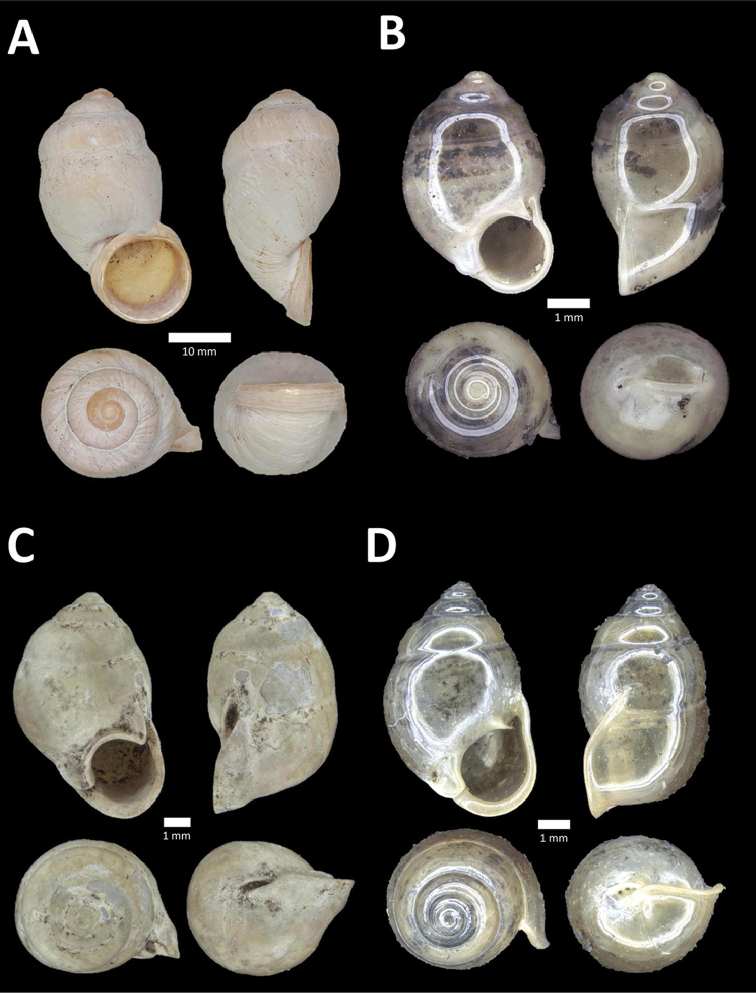
**A** *Pollicaria elephas* (de Morgan, 1885a) BOR/MOL 9443. Perak, Ipoh, Mykarst-025 Plot 1 **B** *Pupina artata* Benson, 1856 BOR/MOL 9803. Perak, Ipoh, Bat Cave Hill Plot 2 **C** *Pupina arula perakensis* von Möllendorff, 1891 BOR/MOL 10566. Perak, Ipoh, Gunung Datok Plot **D** *Pupina lowi* de Morgan, 1885a BOR/MOL 9873. Perak, Ipoh, Bat Cave Hill Plot 4.

###### Genus *Pupina* Vignard, 1829

####### Pupina artata

Taxon classificationAnimaliaMesogastropodaPupinidae

Benson, 1856

Figure 15B

######## Materials examined.

mykarst-184 Bat Cave: BOR/MOL 9872, BOR/MOL 9803, BOR/MOL 9835, BOR/MOL 9777. mykarst-025: BOR/MOL 9413, BOR/MOL 9486, BOR/MOL 9505, BOR/MOL 12425. Prk 42 G. Bercham: BOR/MOL 9460, BOR/MOL 9474, BOR/MOL 9211, BOR/MOL 9234, BOR/MOL 10573, BOR/MOL 10622, BOR/MOL 10627. mykarst-185 Batu Kebelah: BOR/MOL 9534, BOR/MOL 9575, BOR/MOL 9580. Prk 64 Bt Kepala Gajah: BOR/MOL 10092, BOR/MOL 10139, BOR/MOL 10171. Prk 55 G. Pondok: BOR/MOL 11490, BOR/MOL 11525. Prk 01 G. Tempurung: BOR/MOL 11221.

######## Distribution.

In Peninsular Malaysia, found in Perak and Selangor (Maassen 2001). Elsewhere, in Moulmein (=Mawlamyine), Myanmar and Ko Samui, Thailand (Maassen 2001).

######## Remarks.

Shell colour varies from white to brown, glossy. Whorl convexity varies slightly but is always less expanded than congeners. Differ from all sympatric *Pupina* species in being small, whorls less expanded as well as the formation and curvature of the aperture and its narrow canals.

####### Pupina arula perakensis

Taxon classificationAnimaliaMesogastropodaPupinidae

von Möllendorff, 1891

Figure 15C

######## Materials examined.

Prk 36 Gua Datok: BOR/MOL 10566. Kampung Pahit limestone outcrop, Klian Intan, Perak: BOR/MOL 7091.

######## Distribution.

Known from Perak only (Maassen 2001).

######## Remarks.

Shell colour brown, glossy when alive. Whorl more convex and expanded than most congeners. Shell larger than most sympatric congeners. Distinguished from congeners by the formation and curvature of the aperture and its canals, as well as the peristome extension right of the penultimate whorl.

####### Pupina lowi

Taxon classificationAnimaliaMesogastropodaPupinidae

de Morgan, 1885a

Figure 15D

######## Materials examined.

Prk 53 Hill KF: BOR/MOL 10706, BOR/MOL 10718, BOR/MOL 10748, BOR/MOL 10666. mykarst-184 Bat Cave: BOR/MOL 9873, BOR/MOL 9780, BOR/MOL 9800. mykarst-185 Batu Kebelah: BOR/MOL 9535, BOR/MOL 9566, BOR/MOL 9576, BOR/MOL 9581. Prk 64 Bt Kepala Gajah: BOR/MOL 10125.

######## Distribution.

Known from Perlis, Perak and Kelantan only (Maassen 2001).

######## Remarks.

Shell colour brown, glossy when alive. Radial striations of equal width occasionally present. Most similar to *Pupina arula perakensis* in its rounded whorls but differ by its taller spire, the open upper canal and the peristome extension right of the penultimate whorl.

####### Pupina tchehelensis

Taxon classificationAnimaliaMesogastropodaPupinidae

de Morgan, 1885b

Figure 16A

######## Materials examined.

Prk 47 Kanthan: BOR/MOL 9177, BOR/MOL 9072. mykarst-025: BOR/MOL 9387, BOR/MOL 9417, BOR/MOL 9491, BOR/MOL 9509. mykarst-027: BOR/MOL 9096, BOR/MOL 9028. Prk 23 G. Rapat: BOR/MOL 10208, BOR/MOL 10034, BOR/MOL 10051, BOR/MOL 10231, BOR/MOL 10261. Prk 42 G. Bercham: BOR/MOL 10654, BOR/MOL 10605. Prk 36 Gua Datok: BOR/MOL 10450, BOR/MOL 10480, BOR/MOL 10499. Prk 34 G. Tasek: BOR/MOL 11024, BOR/MOL 11063. Prk 01 G. Tempurung: BOR/MOL 11141, BOR/MOL 11216, BOR/MOL 11393, BOR/MOL 11419. Prk 55 G. Pondok: BOR/MOL 11510.

######## Distribution.

Known from Perak only (Maassen 2001).

######## Remarks.

Shell colour brown, glossy when alive. Variable in shell size. Whorls slender. Spire tall. Distinguished from congeners by the peristomal thickening and curvature of the aperture and its canals in mature shells.

**Figure 16.**
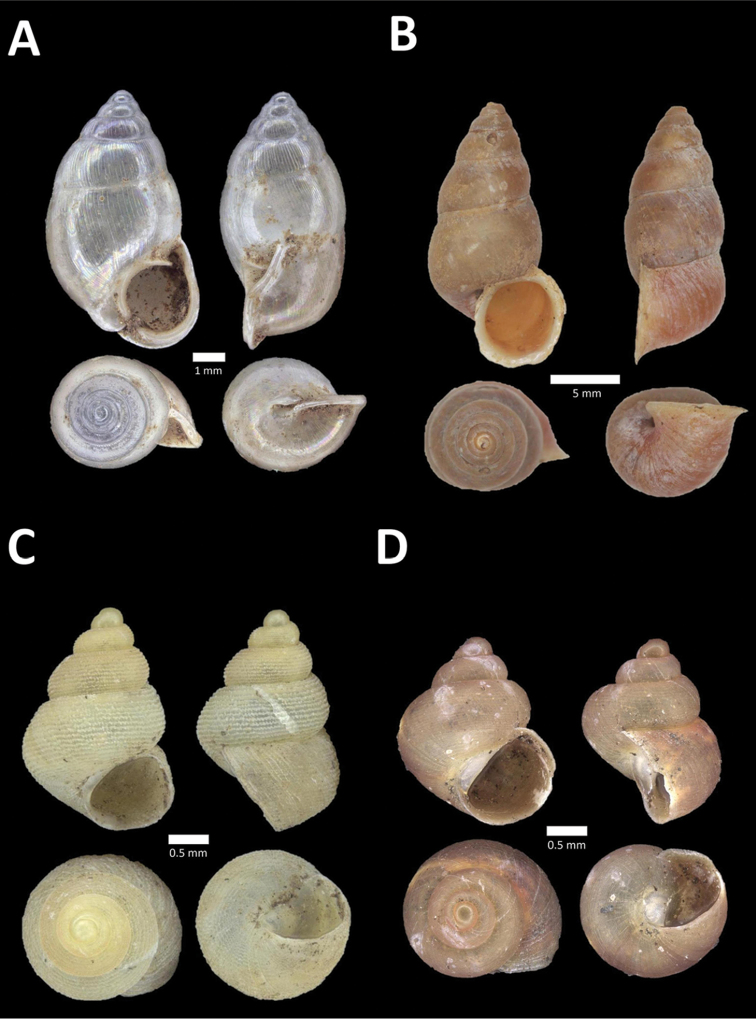
**A** *Pupina tchehelensis* de Morgan, 1885b BOR/MOL 11393. Perak, Ipoh, Gunung Tempurung Plot 2 **B** *Schistoloma sectilabrum* (Gould, 1844) BOR/MOL 8103. Perak, Ipoh, forested slope behind village at Gunung Pondok **C** *Georissa monterosatiana* Godwin-Austen & Nevill, 1879 BOR/MOL 11524. Perak, Ipoh, Gunung Pondok **D** *Georissa semisculpta* Godwin-Austen & Nevill, 1879 BOR/MOL 11047. Perak, Ipoh, Gunung Tasek.

###### Genus *Schistoloma* Kobelt, 1902

####### Schistoloma sectilabrum

Taxon classificationAnimaliaMesogastropodaPupinidae

(Gould, 1844)

Figure 16B

######## Materials examined.

Prk 47 Kanthan: BOR/MOL 9184. mykarst-184 Bat Cave: BOR/MOL 9765, BOR/MOL 9798.

######## Distribution.

In Peninsular Malaysia, known from Penang, Perak and Pahang (Maassen 2001, Tumpeesuwan and Panha 2008). Elsewhere, in Petchaburi, Thailand as well as Tavoy (=Dawei) and Tenasserim (=Tanintharyi), Myanmar (Tumpeesuwan and Panha 2008).

######## Remarks.

Shell colour brown. Spire tall. Peristome thin, reflected and expanded. Differ from *Pupina* species in the lack of apertural canals. Differ from *Pollicaria* species in its smaller size and straighter coiling axis.

#### Unranked clade: Neritimorpha

##### Family Hydrocenidae Troschel, 1857

###### Genus *Georissa* Blanford, 1864

####### Georissa monterosatiana

Taxon classificationAnimaliaCycloneritimorphaHydrocenidae

Godwin-Austen & Nevill, 1879

Figure 16C

######## Materials examined.

Prk 53 Hill KF: BOR/MOL 10704, BOR/MOL 10754, BOR/MOL 10730, BOR/MOL 10669. Prk 47 Kanthan: BOR/MOL 9084, BOR/MOL 9156. mykarst-184 Bat Cave: BOR/MOL 9868, BOR/MOL 9816, BOR/MOL 9838, BOR/MOL 9776. mykarst-027: BOR/MOL 9044, BOR/MOL 9132. mykarst-185 Batu Kebelah: BOR/MOL 9547, BOR/MOL 9591. Prk 23 G. Rapat: BOR/MOL 10043, BOR/MOL 10240, BOR/MOL 10264. Prk 36 Gua Datok: BOR/MOL 10066, BOR/MOL 10430, BOR/MOL 10459. Prk 64 Bt Kepala Gajah: BOR/MOL 10189. Prk 42 G. Bercham: BOR/MOL 10606. Prk 01 G. Tempurung: BOR/MOL 11152, BOR/MOL 11228, BOR/MOL 11396, BOR/MOL 11423. Prk 55 G. Pondok: BOR/MOL 11499, BOR/MOL 11524, BOR/MOL 11544, BOR/MOL 11569. Prk 34 G. Tasek: BOR/MOL 11178.

######## Distribution.

In Peninsular Malaysia, known from Perak, Kelantan, Pahang, Perlis and Selangor (Maassen 2001). Elsewhere, in Jalor (=Yala), Thailand (Maassen 2001).

######## Remarks.

Shell small. Differ from the sympatric *Georissa semisculpta* by its taller spire, pronounced and crenulated spiral ridges as well as less obtuse whorls. Shell colour yellow to red.

####### Georissa semisculpta

Taxon classificationAnimaliaCycloneritimorphaHydrocenidae

Godwin-Austen & Nevill, 1879

Figure 16D

######## Materials examined.

Prk 36 Gua Datok: BOR/MOL 10067. Prk 64 Bt Kepala Gajah: BOR/MOL 10105, BOR/MOL 10149, BOR/MOL 10188. Prk 01 G. Tempurung: BOR/MOL 10670, BOR/MOL 11151, BOR/MOL 12083. Prk 23 G. Rapat: BOR/MOL 10241, BOR/MOL 12092. Prk 42 G. Bercham: BOR/MOL 10580. Prk 34 G. Tasek: BOR/MOL 11001, BOR/MOL 11047, BOR/MOL 11058, BOR/MOL 11177. mykarst-184 Bat Cave: BOR/MOL 12082, BOR/MOL 12084, BOR/MOL 12085, BOR/MOL 12091. Prk 47 Kanthan: BOR/MOL 12086. mykarst-185 Batu Kebelah: BOR/MOL 12087. mykarst-027: BOR/MOL 12088, BOR/MOL 12090. Prk 53 Hill KF: BOR/MOL 12089.

######## Distribution.

Known from Perak and Pahang only (Maassen 2001).

######## Remarks.

Shell small. Differ from the sympatric *Georissa monterosatiana* by its lower spire, indistinct spiral sculpture over radial growth lines and very obtuse whorls. Shell colour pink to red. New record for Kinta Valley. Prior to this study, *G. semisculpta* was thought to be extinct because it was presumed endemic to Gunung Pondok, where it has not been detected ever since its original description (Davison 1991).

#### Clade Heterobranchia

##### Informal group Pulmonata Cuvier, in de Blainville 1814

###### Family Achatinellidae Gulick, 1873

####### Genus *Elasmias* Pilsbry, 1910

######## Elasmias terrestris

Taxon classificationAnimaliaStylommatophoraAchatinellidae

(Brazier, 1876)

Figure 17A

######### Materials examined.

mykarst-184 Bat Cave: BOR/MOL 9790, BOR/MOL 9817, BOR/MOL 9840. Prk 64 Bt Kepala Gajah: BOR/MOL 10186. Prk 55 G. Pondok: BOR/MOL 11528.

######### Distribution.

In Peninsular Malaysia, found in Kedah and Perak (Maassen 2001). Elsewhere, New Guinea and Australia (Maassen 2001, Solem 1988).

######### Remarks.

Small shell. Distinguished from others by its oviform shell and truncated columella with two lamella teeth (one more pronounced than the other). This is a new record for Perak.

**Figure 17.**
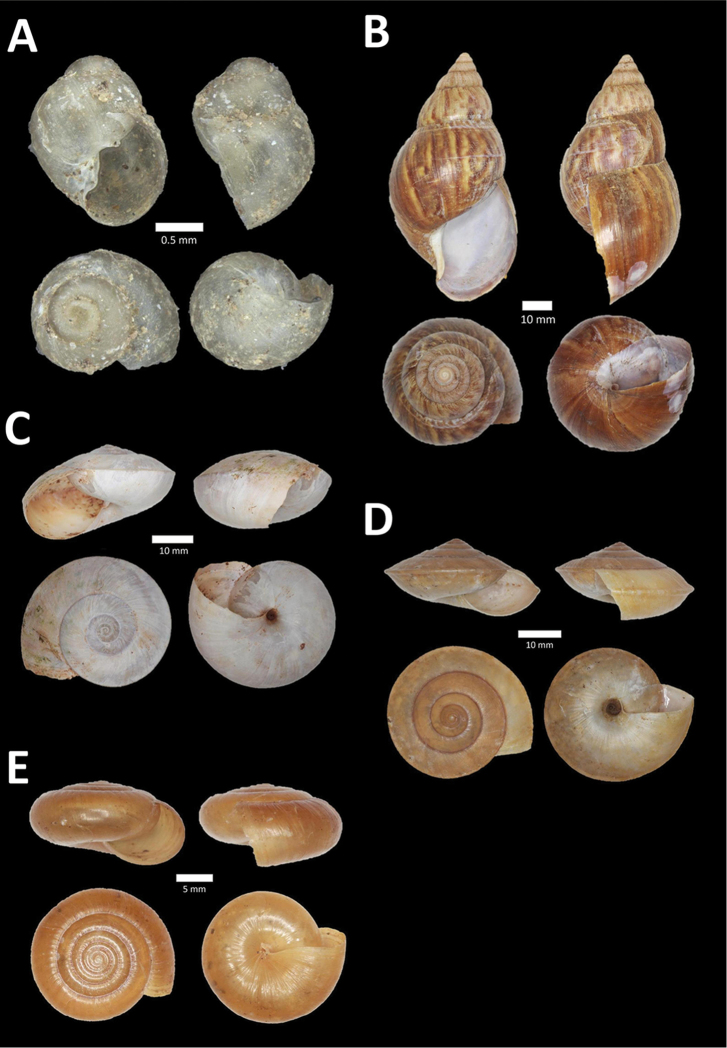
**A** *Elasmias terrestre* (Brazier, 1876) BOR/MOL 10186. Perak, Ipoh, Gua Tok Giring Plot 4 **B** *Achatina fulica* (Bowdich, 1822) BOR/MOL 11208. Perak, Ipoh, Gunung Tempurung Plot 3 **C** *Ariophanta lahatensis* de Morgan, 1885a BOR/MOL 8275. Perak, Ipoh, Iron hill summit trail **D** *Hemiplecta cymatium* (Pfeiffer, 1856) BOR/MOL 11611. Perak, Ipoh, Gunung Pondok, plot 5 **E** *Macrochlamys* ‘Batu Kebelah 1’ BOR/MOL 9528. Perak, Ipoh, Batu Kebelah Plot 1.

###### Family Achatinidae Swainson, 1840

####### Genus *Achatina* Lamarck, 1799

######## Achatina fulica

Taxon classificationAnimaliaStylommatophoraAchatinidae

(Bowdich, 1822)

Figure 17B

######### Materials examined.

mykarst-184 Bat Cave: BOR/MOL 9860. mykarst-025: BOR/MOL 9378, BOR/MOL 9438, BOR/MOL 9497. mykarst-027: BOR/MOL 9046, BOR/MOL 9102. Prk 47 Kanthan: BOR/MOL 9049, BOR/MOL 9141. Prk 42 G. Bercham: BOR/MOL 9207, BOR/MOL 10570. mykarst-185 Batu Kebelah: BOR/MOL 9525, BOR/MOL 9558, BOR/MOL 9568, BOR/MOL 9582. Prk 64 Bt Kepala Gajah: BOR/MOL 10176. Prk 34 G. Tasek: BOR/MOL 10785, BOR/MOL 11025, BOR/MOL 11061, BOR/MOL 11171. Prk 01 G. Tempurung: BOR/MOL 11208, BOR/MOL 11239.

######### Distribution.

Widespread in Peninsular Malaysia (Maassen 2001). Elsewhere, native to tropical Africa but now pantropical (Fontanilla et al. 2014).

######### Remarks.

Large shell. Tall-spired with distinct stripes or flammulations. This is a synanthropic species.

###### Family Ariophantidae Godwin-Austen, 1888

####### Genus *Ariophanta* Desmoulins, 1829

######## Ariophanta lahatensis

Taxon classificationAnimaliaStylommatophoraAriophantidae

(de Morgan, 1885a)

Figure 17C

######### Materials examined.

Prk 47 Kanthan: BOR/MOL 9067, BOR/MOL 9173. mykarst-027: BOR/MOL 9099. mykarst-025: BOR/MOL 9434, BOR/MOL 9504. Prk 23 G. Rapat: BOR/MOL 10054, BOR/MOL 10204, BOR/MOL 10274. Prk 36 Gua Datok: BOR/MOL 10454. Prk 01 G. Tempurung: BOR/MOL 11137, BOR/MOL 11214, BOR/MOL 11247, BOR/MOL 11382, BOR/MOL 11409.

######### Distribution.

Restricted to Kinta Valley (Maassen 2001).

######### Remarks.

Medium-sized shell. Sinistral. Keeled periphery. Colour off-white to translucent brown. Shell surface has gentle radial growth lines overlaid with short spiral crenulations. The species group comprising of *Nanina salangana* von Martens, 1883, *Helix retrosa* Gould, 1844 and *Helix lahatensis* de Morgan, 1885a, was reassigned from Dyakiidae to Ariophantidae based on shell and anatomy (Sutcharit et al. 2012).

####### Genus *Hemiplecta* Albers, 1850

######## Hemiplecta cymatium

Taxon classificationAnimaliaStylommatophoraAriophantidae

(Pfeiffer, 1856)

Figure 17D

######### Materials examined.

mykarst-184 Bat Cave: BOR/MOL 9855, BOR/MOL 9795, BOR/MOL 9825. Prk 53 Hill KF: BOR/MOL 10740, BOR/MOL 10656, BOR/MOL 10686. mykarst-027: BOR/MOL 9104. Prk 55 G. Pondok: BOR/MOL 11611.

######### Distribution.

In the states of Kedah, Penang, Perak, Kelantan and Pahang in Peninsular Malaysia (Maassen 2001). Elsewhere, in Jalor (=Yala), southern Thailand (Maassen 2001).

######### Remarks.

Large shell. Perak shells differ from Penang shells only by their flatter spire. Whorls brown with darker periphery. Peripheral keel pronounced. Shell surface has numerous short spiral crenulations.

####### Genus *Macrochlamys* Godwin-Austen, 1883

######## Macrochlamys

Taxon classificationAnimaliaStylommatophoraAriophantidae

‘Batu Kebelah 1’

Figure 17E

######### Materials examined.

Prk 64 Bt Kepala Gajah: BOR/MOL 10114, BOR/MOL 10118, BOR/MOL 10128, BOR/MOL 10129, BOR/MOL 10130, BOR/MOL 10168. mykarst-184 Bat Cave: BOR/MOL 9859. mykarst-185 Batu Kebelah: BOR/MOL 9528, BOR/MOL 9561, BOR/MOL 9571. Prk 55 G. Pondok: BOR/MOL 11612, BOR/MOL 11508, BOR/MOL 11564.

######### Distribution.

Known from upper Kinta Valley and Perak River Valley, Perak only.

######### Remarks.

Medium-sized shell. Whorls more tightly coiled than congeners, spire flat. Reddish brown shell with pronounced undulations of radial growth lines along the suture.

######## Macrochlamys

Taxon classificationAnimaliaStylommatophoraAriophantidae

‘Bercham 1’

Figure 18A

######### Materials examined.

mykarst-184 Bat Cave: BOR/MOL 9861, BOR/MOL 9828, BOR/MOL 9775, BOR/MOL 9806. Prk 53 Hill KF: BOR/MOL 10737, BOR/MOL 10771, BOR/MOL 10777, BOR/MOL 10676. Prk 42 G. Bercham: BOR/MOL 9468, BOR/MOL 9477, BOR/MOL 9212, BOR/MOL 9239, BOR/MOL 10645. Prk 23 G. Rapat: BOR/MOL 10223, BOR/MOL 10281, BOR/MOL 10283. mykarst-027: BOR/MOL 9199. Prk 47 Kanthan: BOR/MOL 9203. mykarst-185 Batu Kebelah: BOR/MOL 9556, BOR/MOL 9572, BOR/MOL 9588. Prk 36 Gua Datok: BOR/MOL 10081, BOR/MOL 10466, BOR/MOL 10483. Prk 34 G. Tasek: BOR/MOL 11011.

######### Distribution.

Known from Kinta Valley, Perak only.

######### Remarks.

Shell small relative to congeners. Rounded periphery, spire relatively tall, whorls wide. Overall, shell looks glossy. Radial sculpture indistinct, spiral grooves distinct but very fine. Suture indistinct. Most similar to *Macrochlamys malayana* (von Möllendorff, 1891), which differs in being more globose and has less whorls.

**Figure 18.**
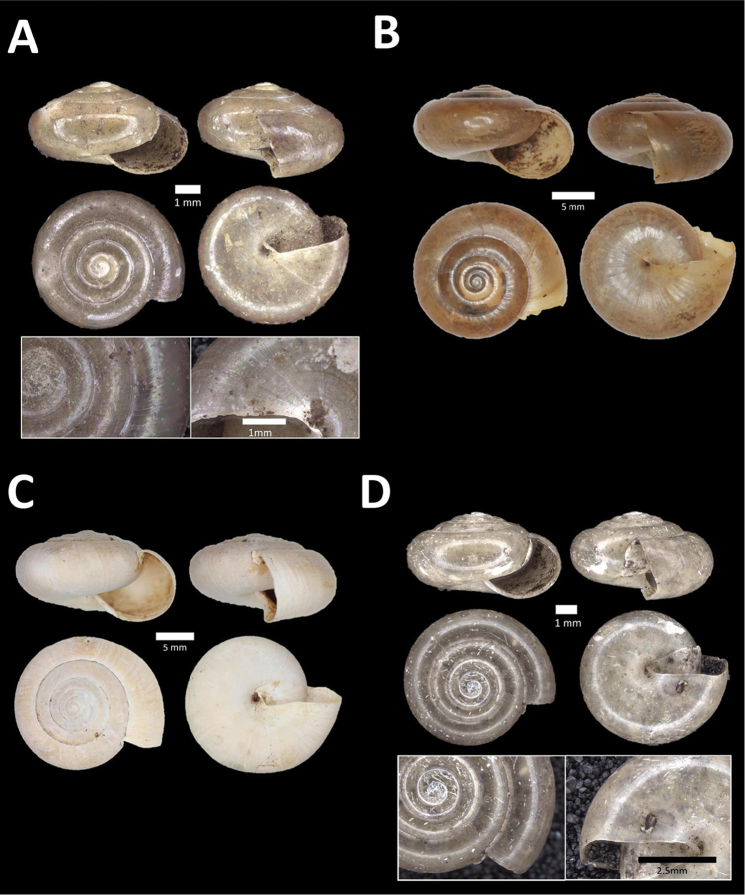
**A** *Macrochlamys* ‘Bercham 1’ BOR/MOL 9477. Perak, Ipoh, Gunung Bercham Plot 3 **B** *Macrochlamys malaccana* (Pfeiffer, 1854) BOR/MOL 9570. Perak, Ipoh, Batu Kebelah Plot 4 **C** *Macrochlamys* ‘tempurung 1’ BOR/MOL 11134. Perak, Ipoh, Gunung Tempurung Plot 1 **D** *Macrochlamys* ‘tempurung 2’ BOR/MOL 11449. Perak, Ipoh, Gunung Tempurung Plot 2.

######## Macrochlamys malaccana

Taxon classificationAnimaliaStylommatophoraAriophantidae

(Pfeiffer, 1854)

Figure 18B

######### Materials examined.

mykarst-184 Bat Cave: BOR/MOL 9858. mykarst-185 Batu Kebelah: BOR/MOL 9762, BOR/MOL 9527, BOR/MOL 9562, BOR/MOL 9570. Prk 53 Hill KF: BOR/MOL 10712, BOR/MOL 10736, BOR/MOL 10765, BOR/MOL 10690. Prk 42 G. Bercham: BOR/MOL 9228. Prk 36 Gua Datok: BOR/MOL 10415, BOR/MOL 10465, BOR/MOL 10485. Prk 55 G. Pondok: BOR/MOL 12093.

######### Distribution.

Known from Kedah and Perak only.

######### Remarks.

Medium-sized shell. Whorls more expanded than *Macrochlamys* ‘batu kebelah 1’. Suture has distinct dark brown line. Spiral striations are very fine. This species was previously known from Kedah only (Maassen 2001).

######## Macrochlamys

Taxon classificationAnimaliaStylommatophoraAriophantidae

‘tempurung 1’

Figure 18C

######### Materials examined.

Prk 01 G. Tempurung: BOR/MOL 11134, BOR/MOL 11436.

######### Distribution.

Known from Gunung Tempurung only, but surrounding hills have yet to be adequately surveyed.

######### Remarks.

Small shell. Spire flat and whorl coiling tight like *Macrochlamys* ‘batukebelah 1’ but shell more bulbous than *Macrochlamys* ‘batukebelah 1’.

######## Macrochlamys

Taxon classificationAnimaliaStylommatophoraAriophantidae

‘tempurung 2’

Figure 18D

######### Materials examined.

Prk 01 G. Tempurung: BOR/MOL 11440, BOR/MOL 11443, BOR/MOL 11449, BOR/MOL 11456.

######### Distribution.

Known from Gunung Tempurung, but surrounding hills have yet to be adequately surveyed.

######### Remarks.

Shell small relative to congeners. Rounded periphery, spire relatively tall, whorls wide. Overall, shell looks glossy. Radial sculpture more indistinct than *Macrochlamys* ‘Bercham 1’, spiral grooves indistinct and very fine. Suture distinct, each whorl distinguished by its convexity. Most similar to *Macrochlamys* ‘Bercham 1’.

####### Genus *Microcystina* Mörch, 1872

######## Microcystina clarkae

Taxon classificationAnimaliaStylommatophoraAriophantidae

Maassen, 2000

Figure 19A

######### Materials examined.

Prk 47 Kanthan: BOR/MOL 9186, BOR/MOL 9193. mykarst-184 Bat Cave: BOR/MOL 9852, BOR/MOL 9887, BOR/MOL 9792, BOR/MOL 9812. mykarst-185 Batu Kebelah: BOR/MOL 9760, BOR/MOL 9555. Prk 53 Hill KF: BOR/MOL 10758, BOR/MOL 10764, BOR/MOL 10768, BOR/MOL 10774. mykarst-025: BOR/MOL 9399, BOR/MOL 9428, BOR/MOL 9495. mykarst-027: BOR/MOL 9453, BOR/MOL 9201. Prk 42 G. Bercham: BOR/MOL 10650, BOR/MOL 9237. Prk 36 Gua Datok: BOR/MOL 10072, BOR/MOL 10080, BOR/MOL 10438. Prk 64 Bt Kepala Gajah: BOR/MOL 10108, BOR/MOL 10159, BOR/MOL 10195. Prk 55 G. Pondok: BOR/MOL 11602, BOR/MOL 11606, BOR/MOL 11593, BOR/MOL 11594. Prk 34 G. Tasek: BOR/MOL 11122, BOR/MOL 11127, BOR/MOL 11198, BOR/MOL 11199. Prk 01 G. Tempurung: BOR/MOL 11442, BOR/MOL 11445.

######### Distribution.

In Peninsular Malaysia, only known from Perak. Elsewhere, in Sumatra, Indonesia (Maassen 2000).

######### Remarks.

Very small shell. Easily distinguished from congeners by its white shell, tight whorls, fine radial ribs and finer spiral pits. This is a new record for Peninsular Malaysia.

**Figure 19.**
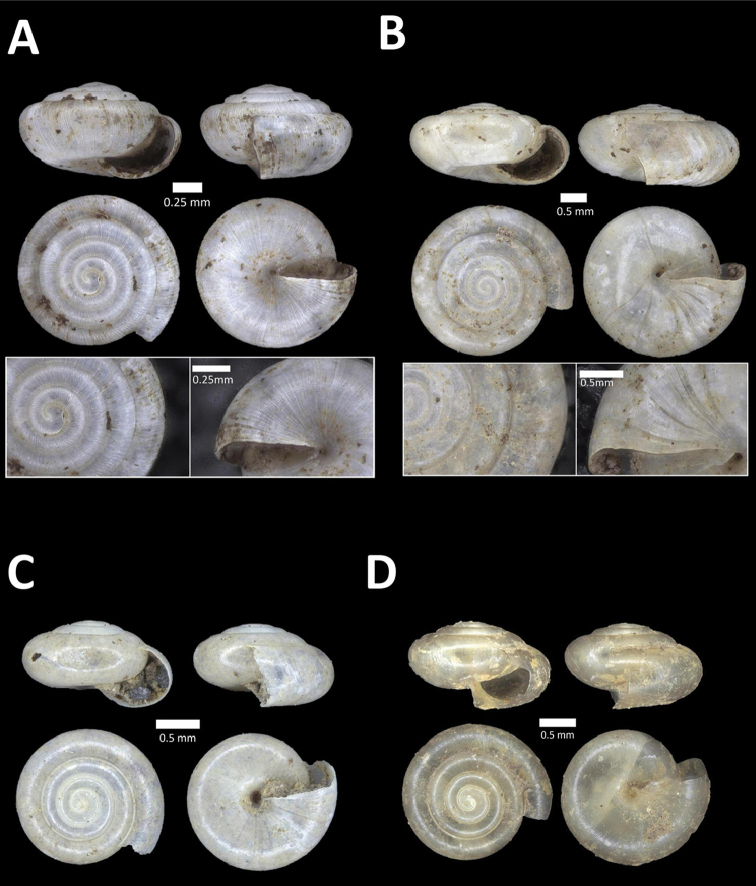
**A** *Microcystina clarkae* Maassen, 2000 BOR/MOL 9186. Perak, Ipoh, Gunung Kanthan Plot 4 **B** *Microcystina* ‘guatokgiring 1’ BOR/MOL 10109. Perak, Ipoh, Gua Tok Giring Plot 1 **C** *Microcystina* ‘guatokgiring 2’ BOR/MOL 10116. Perak, Ipoh, Gua Tok Giring Plot 1 **D** *Microcystina* ‘Kanthan 1’ BOR/MOL 9761. Perak, Ipoh, Batu Kebelah Plot 4.

######## Microcystina

Taxon classificationAnimaliaStylommatophoraAriophantidae

‘guatokgiring 1’

Figure 19B

######### Materials examined.

Prk 64 Bt Kepala Gajah: BOR/MOL 10117, BOR/MOL 10109, BOR/MOL 10110, BOR/MOL 10160, BOR/MOL 10197.

######### Distribution.

Known from Bukit Kepala Gajah, Perak only, but surrounding hills have yet to be adequately surveyed.

######### Remarks.

Small shell. Whorls convex but tight, spire relatively low, dome shaped. Radial sculpture fine, spiral sculpture absent. Shell white.

######## Microcystina

Taxon classificationAnimaliaStylommatophoraAriophantidae

‘guatokgiring 2’

Figure 19C

######### Materials examined.

Prk 64 Bt Kepala Gajah: BOR/MOL 10116, BOR/MOL 10161, BOR/MOL 10196.

######### Distribution.

Known from Bukit Kepala Gajah, Perak only, but surrounding hills have yet to be adequately surveyed.

######### Remarks.

Small shell. Whorls flat and denser than *Microcystina* ‘guatokgiring 1’.

######## Microcystina

Taxon classificationAnimaliaStylommatophoraAriophantidae

‘Kanthan 1’

Figure 19D

######### Materials examined.

mykarst-184 Bat Cave: BOR/MOL 9888, BOR/MOL 9793. mykarst-185 Batu Kebelah: BOR/MOL 9758, BOR/MOL 9761, BOR/MOL 9553, BOR/MOL 9554. Prk 53 Hill KF: BOR/MOL 10757, BOR/MOL 10770, BOR/MOL 10775, BOR/MOL 10778. mykarst-025: BOR/MOL 9401, BOR/MOL 9429, BOR/MOL 9494, BOR/MOL 9519. Prk 42 G. Bercham: BOR/MOL 10652, BOR/MOL 10587, BOR/MOL 10641, BOR/MOL 10643. Prk 47 Kanthan: BOR/MOL 9189, BOR/MOL 9191, BOR/MOL 9206. mykarst-027: BOR/MOL 9197, BOR/MOL 9202. Prk 36 Gua Datok: BOR/MOL 10079, BOR/MOL 10084. Prk 64 Bt Kepala Gajah: BOR/MOL 10198, BOR/MOL 10199. Prk 55 G. Pondok: BOR/MOL 11596, BOR/MOL 11603, BOR/MOL 11605, BOR/MOL 11589. Prk 34 G. Tasek: BOR/MOL 11123, BOR/MOL 11022, BOR/MOL 11128, BOR/MOL 11032, BOR/MOL 11195, BOR/MOL 11200. Prk 01 G. Tempurung: BOR/MOL 11434, BOR/MOL 11438, BOR/MOL 11446, BOR/MOL 11457, BOR/MOL 11458.

######### Distribution.

Known from Perak only.

######### Remarks.

Small shell. Apical whorls with dense, spiral pits. Subsequent whorls spiral lined. Suture line brown. Radial sculpture indistinct. Shell brown.

######## Microcystina

Taxon classificationAnimaliaStylommatophoraAriophantidae

‘pondok 1’

Figure 20A

######### Materials examined.

Prk 55 G. Pondok: BOR/MOL 11597.

######### Distribution.

Known from Gunung Pondok only.

######### Remarks.

Dome shaped spire, netted sculpture.

**Figure 20.**
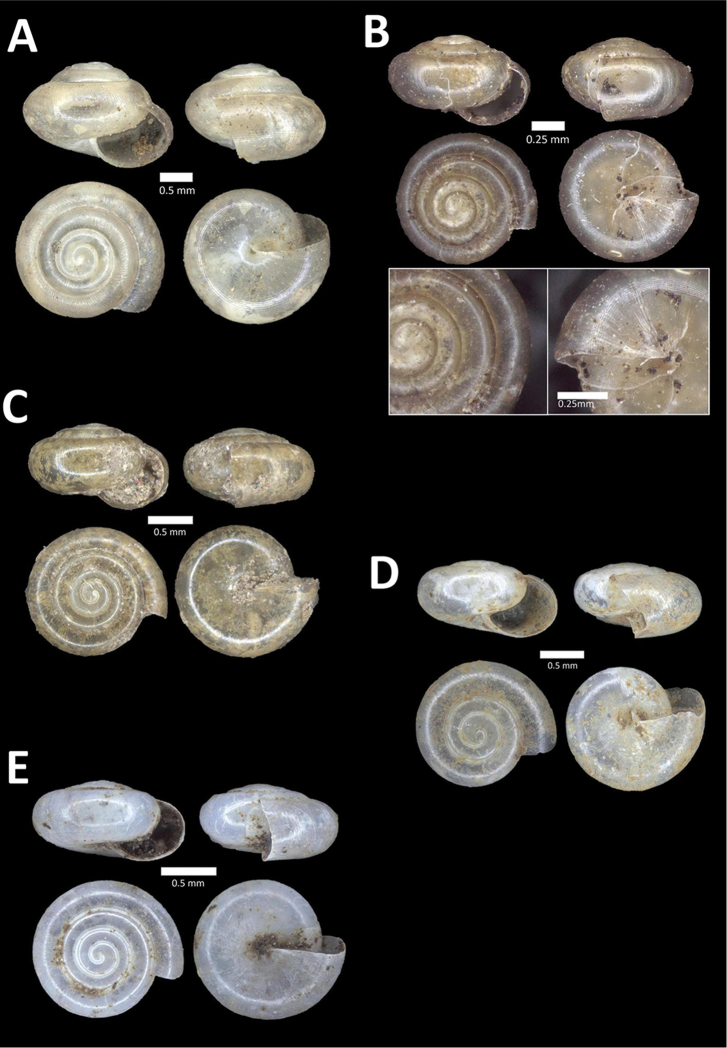
**A** *Microcystina* ‘pondok 1’ BOR/MOL 11597. Perak, Ipoh, Gunung Pondok, plot 1 **B** *Microcystina sinica* von Möllendorff, 1885 BOR/MOL 9192. Perak, Ipoh, Gunung Kanthan Plot 4 **C** *Microcystina* ‘tempurung 2’ BOR/MOL 11450. Perak, Ipoh, Gunung Tempurung Plot 2 **D** *Microcystina* ‘tempurung 3’ BOR/MOL 11451. Perak, Ipoh, Gunung Tempurung Plot 2 **E** *Microcystina townsendiana* Godwin-Austen & Nevill, 1879 BOR/MOL 9187. Perak, Ipoh, Gunung Kanthan.

######## Microcystina sinica

Taxon classificationAnimaliaStylommatophoraAriophantidae

(von Möllendorff, 1885)

Figure 20B

######### Materials examined.

mykarst-184 Bat Cave: BOR/MOL 9886, BOR/MOL 9851, BOR/MOL 9787, BOR/MOL 9823. mykarst-185 Batu Kebelah: BOR/MOL 9757, BOR/MOL 9549. Prk 53 Hill KF: BOR/MOL 10756, BOR/MOL 10772. mykarst-025: BOR/MOL 9398, BOR/MOL 9427, BOR/MOL 9493, BOR/MOL 9518. mykarst-027: BOR/MOL 9455, BOR/MOL 9204. Prk 42 G. Bercham: BOR/MOL 9472, BOR/MOL 9223, BOR/MOL 10646, BOR/MOL 10648. Prk 23 G. Rapat: BOR/MOL 10222, BOR/MOL 10279. Prk 47 Kanthan: BOR/MOL 9192, BOR/MOL 9205. Prk 36 Gua Datok: BOR/MOL 10073, BOR/MOL 10077, BOR/MOL 10439. Prk 64 Bt Kepala Gajah: BOR/MOL 10194. Prk 55 G. Pondok: BOR/MOL 11595, BOR/MOL 11610, BOR/MOL 11592. Prk 34 G. Tasek: BOR/MOL 11126, BOR/MOL 11201. Prk 01 G. Tempurung: BOR/MOL 11437, BOR/MOL 11453, BOR/MOL 11452, BOR/MOL 11454.

######### Distribution.

In Peninsular Malaysia, known from Perak only (Maassen 2001). Elsewhere, south China and island Southeast Asia to western New Guinea (Vermeulen et al. 2015).

######### Remarks.

Very small shell. Easily distinguished from congeners by its brown shell, domed spire, tight whorls, fine spiral lines and dark brown suture line.

######## Microcystina

Taxon classificationAnimaliaStylommatophoraAriophantidae

‘tempurung 2’

Figure 20C

######### Materials examined.

Prk 01 G. Tempurung: BOR/MOL 11450.

######### Distribution.

Known from Gunung Tempurung, Perak only, but surrounding hills have yet to be adequately surveyed.

######### Remarks.

Shell shape similar to *Microcystina sinica*, differ in the absence of spiral sculpture.

######## Microcystina

Taxon classificationAnimaliaStylommatophoraAriophantidae

‘tempurung 3’

Figure 20D

######### Materials examined.

Prk 01 G. Tempurung: BOR/MOL 11451.

######### Distribution.

Known from Gunung Tempurung, Perak only, but surrounding hills have yet to be adequately surveyed.

######### Remarks.

Shell somewhat similar to *Microcystina townsendiana* but differ in having wider whorls and the different manner of spiral and radial sculpture.

######## Microcystina townsendiana

Taxon classificationAnimaliaStylommatophoraAriophantidae

Godwin-Austen & Nevill, 1879

Figure 20E

######### Materials examined.

Prk 47 Kanthan: BOR/MOL 9187, BOR/MOL 9174, BOR/MOL 9190, BOR/MOL 9194. mykarst-184 Bat Cave: BOR/MOL 9853, BOR/MOL 9890, BOR/MOL 9788, BOR/MOL 9822, BOR/MOL 9844. mykarst-185 Batu Kebelah: BOR/MOL 9756, BOR/MOL 9550. Prk 53 Hill KF: BOR/MOL 10759, BOR/MOL 10761, BOR/MOL 10766, BOR/MOL 10776. mykarst-025: BOR/MOL 9400, BOR/MOL 9430, BOR/MOL 9496, BOR/MOL 9521. mykarst-027: BOR/MOL 9454, BOR/MOL 9200. Prk 23 G. Rapat: BOR/MOL 10220, BOR/MOL 10041, BOR/MOL 10273, BOR/MOL 10277, BOR/MOL 10278. Prk 42 G. Bercham: BOR/MOL 10651, BOR/MOL 9224, BOR/MOL 9226, BOR/MOL 9238, BOR/MOL 10585, BOR/MOL 10642, BOR/MOL 10644, BOR/MOL 10647. Prk 36 Gua Datok: BOR/MOL 10070, BOR/MOL 10074, BOR/MOL 10078, BOR/MOL 10437. Prk 55 G. Pondok: BOR/MOL 11598, BOR/MOL 11600, BOR/MOL 11601, BOR/MOL 11608, BOR/MOL 11609, BOR/MOL 11591. Prk 34 G. Tasek: BOR/MOL 11121, BOR/MOL 11023, BOR/MOL 11129, BOR/MOL 11197, BOR/MOL 11202. Prk 01 G. Tempurung: BOR/MOL 11435, BOR/MOL 11439, BOR/MOL 11447, BOR/MOL 11448.

######### Distribution.

Known from Perak and Pahang only (Maassen 2001).

######### Remarks.

Small shell. Flat spire. Spiral sculpture fine, widely spaced. Whorls narrower than *Microcystina* ‘tempurung 3’.

###### Family Bradybaenidae Pilsbry, 1939

####### Genus *Bradybaena* Beck, 1837

######## Bradybaena similaris

Taxon classificationAnimaliaStylommatophoraBradybaenidae

(Férussac, 1821)

Figure 21A

######### Materials examined.

mykarst-025: BOR/MOL 9376. Prk 64 Bt Kepala Gajah: BOR/MOL 10090. Prk 34 G. Tasek: BOR/MOL 11003.

######### Distribution.

Widespread in Peninsular Malaysia (Maassen 2001). Native to East and Southeast Asia but now pantropical (de Winter et al. 2009).

######### Remarks.

Medium-sized shell. Globular shell. Spire height low to medium. Umbilicus present. Periphery reflected when mature. Shell white to brown, occasionally with dark brown peripheral band. This is a synanthropic species.

**Figure 21.**
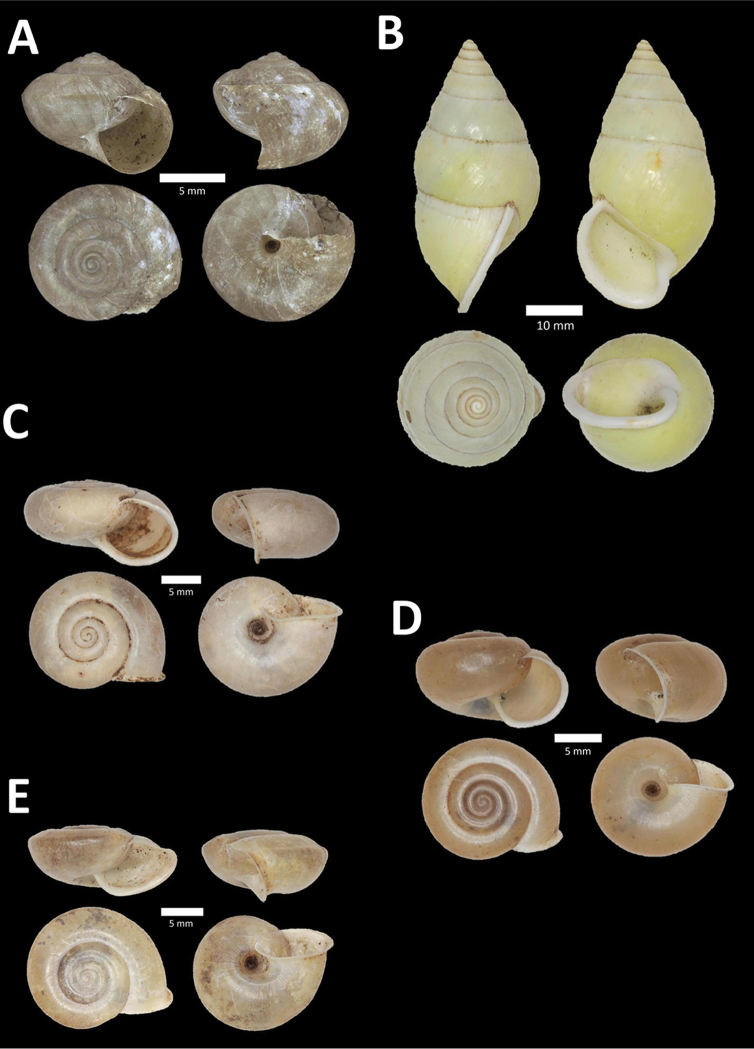
**A** *Bradybaena similaris* (Férussac, 1821) BOR/MOL 9376. Perak, Ipoh, Mykarst-025 Plot 1 **B** *Amphidromus atricallosus perakensis* (Fulton, 1901) BOR/MOL 8285. Perak, Ipoh, outcrop 500m **C** *Chloritis breviseta* (Pfeiffer, 1862) BOR/MOL 9091. Perak, Ipoh, Gunung Kanthan Plot 2 **D** *Chloritis penangensis* (Stoliczka, 1873) BOR/MOL 11562. Perak, Ipoh, Gunung Pondok, plot 6 **E** *Trachia wrayi* (de Morgan, 1885b) BOR/MOL 9381. Perak, Ipoh, Mykarst-025 Plot 1.

###### Family Camaenidae Pilsbry, 1895

####### Genus *Amphidromus* Albers, 1850

######## Amphidromus atricallosus perakensis

Taxon classificationAnimaliaStylommatophoraCamaenidae

(Fulton, 1901)

Figure 21B

######### Materials examined.

Prk 47 Kanthan: BOR/MOL 9051.mykarst-027: BOR/MOL 9103. Prk 23 G. Rapat: BOR/MOL 10028. Prk 01 G. Tempurung: BOR/MOL 11139, BOR/MOL 11210, BOR/MOL 11243.

######### Distribution.

Widespread in Peninsular Malaysia (Maassen 2001, Tan et al. 2011).

######### Remarks.

Large shell. Tall spire. Dimorphic coiling within populations, with dextral and sinistral individuals. Shell lemon yellow throughout, with white sub-sutural line. Prior to this, among Kinta Valley limestone hills, Davison (1991) reported *A. atricallosus perakensis* from Gunung Kanthan only.

####### Genus *Chloritis* Beck, 1837

######## Chloritis breviseta

Taxon classificationAnimaliaStylommatophoraCamaenidae

(Pfeiffer, 1862)

Figure 21C

######### Materials examined.

mykarst-027: BOR/MOL 9091, BOR/MOL 9016. Prk 47 Kanthan: BOR/MOL 9054, BOR/MOL 9137.

######### Distribution.

In Peninsular Malaysia, known from Kelantan and Perak (Maassen 2001). Elsewhere, in Thailand (Maassen 2001).

######### Remarks.

Medium-sized shell. Spire almost flat. Peristome reflected, more outwardly elongated than *Chloritis penangensis*. Shell flatter than *C. penangensis*.

######## Chloritis penangensis

Taxon classificationAnimaliaStylommatophoraCamaenidae

(Stoliczka, 1873)

Figure 21D

######### Materials examined.

mykarst-184 Bat Cave: BOR/MOL 9881. Prk 53 Hill KF: BOR/MOL 10715, BOR/MOL 10659. mykarst-025: BOR/MOL 9379, BOR/MOL 9405, BOR/MOL 9433, BOR/MOL 9501. mykarst-027: BOR/MOL 9093. Prk 42 G. Bercham: BOR/MOL 9469, BOR/MOL 9209, BOR/MOL 10589, BOR/MOL 10615, BOR/MOL 10637. Prk 23 G. Rapat: BOR/MOL 10229, BOR/MOL 10038, BOR/MOL 10050, BOR/MOL 10252. Prk 36 Gua Datok: BOR/MOL 10075, BOR/MOL 10413, BOR/MOL 10447, BOR/MOL 10477, BOR/MOL 10493. Prk 55 G. Pondok: BOR/MOL 11503, BOR/MOL 11543, BOR/MOL 11562. Prk 34 G. Tasek: BOR/MOL 11062, BOR/MOL 11190. Prk 01 G. Tempurung: BOR/MOL 11380.

######### Distribution.

Known from Penang and Perak only (Maassen 2001).

######### Remarks.

Medium-sized shell. Spire almost flat. Peristome reflected, more rounded than *Chloritis breviseta*. Shell more globular than *C. breviseta*.

####### Genus *Trachia* Albers, 1860

######## Trachia wrayi

Taxon classificationAnimaliaStylommatophoraCamaenidae

(de Morgan, 1885b)

Figure 21E

######### Materials examined.

mykarst-025: BOR/MOL 9381. Prk 34 G. Tasek: BOR/MOL 10789. Prk 01 G. Tempurung: BOR/MOL 11246.

######### Distribution.

Known from Kinta Valley only (Maassen 2001).

######### Remarks.

Medium-sized shell. Spire almost flat. Periphery keeled. Peristome reflected. Reflected upper peristome slightly distended towards the periphery.

###### Family Charopidae Hutton, 1884

####### Genus *Charopa* von Martens, 1860

######## Charopa

Taxon classificationAnimaliaStylommatophoraCharopidae

‘Kanthan 1’

Figure 22A

######### Materials examined.

mykarst-184 Bat Cave: BOR/MOL 9889, BOR/MOL 9774, BOR/MOL 9807, BOR/MOL 9843. mykarst-185 Batu Kebelah: BOR/MOL 9759, BOR/MOL 9552. Prk 53 Hill KF: BOR/MOL 10760, BOR/MOL 10762, BOR/MOL 10767, BOR/MOL 10773. Prk 23 G. Rapat: BOR/MOL 10221, BOR/MOL 10042, BOR/MOL 10275, BOR/MOL 10280. Prk 42 G. Bercham: BOR/MOL 10649, BOR/MOL 9225. Prk 47 Kanthan: BOR/MOL 9188, BOR/MOL 9196. mykarst-027: BOR/MOL 9198.mykarst-025: BOR/MOL 9520. Prk 36 Gua Datok: BOR/MOL 10071, BOR/MOL 10076, BOR/MOL 10083, BOR/MOL 10436. Prk 55 G. Pondok: BOR/MOL 11599, BOR/MOL 11604, BOR/MOL 11607, BOR/MOL 11590. Prk 34 G. Tasek: BOR/MOL 11120, BOR/MOL 11125, BOR/MOL 11021, BOR/MOL 11196. Prk 01 G. Tempurung: BOR/MOL 11441, BOR/MOL 11444, BOR/MOL 11455.

######### Distribution.

Known from Gunung Pondok and Kinta Valley, Perak only.

######### Remarks.

Small shell. Whorls lesser than congeners. Dense nodules in all whorls. Latter whorls sometimes have nodules fused into wavy radial ridges. Shell white.

**Figure 22.**
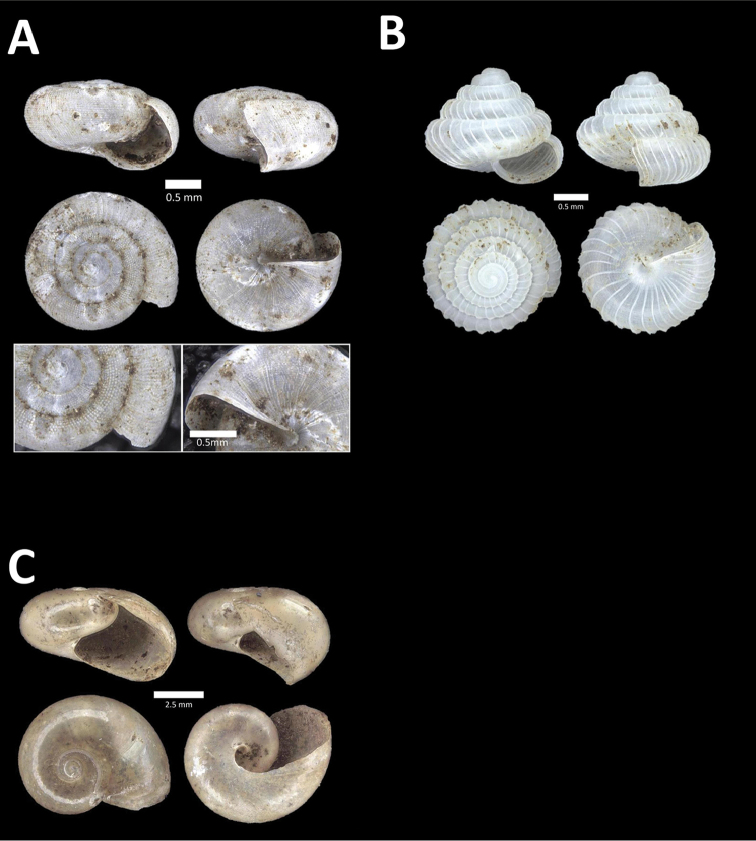
**A** *Charopa* ‘Kanthan 1’ BOR/MOL 9188. Perak, Ipoh, Gunung Kanthan Plot 4 **B** *Charopa lafargei* Vermeulen & Marzuki, 2014 BOR/MOL 9033. Perak, Ipoh, Gunung Kanthan Plot 1 **C** *Vitrinopsis nucleata* (Stoliczka, 1873) BOR/MOL 10150. Perak, Ipoh, Gua Tok Giring Plot 3.

######## Charopa lafargei

Taxon classificationAnimaliaStylommatophoraCharopidae

Vermeulen & Marzuki, 2014

Figure 22B

######### Materials examined.

mykarst-184 Bat Cave: BOR/MOL 9854, BOR/MOL 9863, BOR/MOL 9778, BOR/MOL 9811. mykarst-027: BOR/MOL 9033, BOR/MOL 9124. Prk 47 Kanthan: BOR/MOL 9147. mykarst-185 Batu Kebelah: BOR/MOL 9529, BOR/MOL 9589. Prk 64 Bt Kepala Gajah: BOR/MOL 10179.

######### Distribution.

Known from upper Kinta Valley only.

######### Remarks.

Small shell. Spire tall. Periphery rounded, almost keeled. Radial ribs pronounced, spaced out and equidistant from each other. Spiral ridges distinct but not as pronounced as radial ribs. This species was previously presumed endemic to Gunung Kanthan (Vermeulen and Marzuki 2014).

###### Family Chronidae Thiele, 1931

####### Genus *Vitrinopsis* Semper, 1873

######## Vitrinopsis nucleata

Taxon classificationAnimaliaStylommatophoraChronidae

(Stoliczka, 1873)

Figure 22C

######### Materials examined.

Prk 47 Kanthan: BOR/MOL 9057. Prk 64 Bt Kepala Gajah: BOR/MOL 10150, BOR/MOL 10174. Prk 36 Gua Datok: BOR/MOL 10567.

######### Distribution.

In Peninsular Malaysia, known from Penang and Perak (Maassen 2001). Elsewhere, in Salanga (=Phuket), Thailand (von Martens, 1883).

######### Remarks.

Flat spire. Whorls rapidly widened. Shell brown, glossy. This is a new record for Kinta Valley.

###### Family Clausiliidae Gray, 1855

####### Genus *Phaedusa* Adams & Adams, 1855

######## Phaedusa filicostata kapayanensis

Taxon classificationAnimaliaStylommatophoraClausiliidae

(de Morgan, 1885b)

Figure 23A

######### Materials examined.

Prk 23 G. Rapat: BOR/MOL 10029, BOR/MOL 10236, BOR/MOL 10256. Prk 36 Gua Datok: BOR/MOL 10469, BOR/MOL 10498. Prk 01 G. Tempurung: BOR/MOL 11149, BOR/MOL 11412. Prk 34 G. Tasek: BOR/MOL 11005, BOR/MOL 11194.

######### Distribution.

Known from Perlis, Kedah, Penang, Perak, Kelantan and Pahang (Maassen 2001).

######### Remarks.

**Shell** Spire very tall, slender. Aperture small. Shell smaller and lamella more pronounced than *Phaedusa filicostata tenuicosta* (Loosjes 1953).

**Figure 23.**
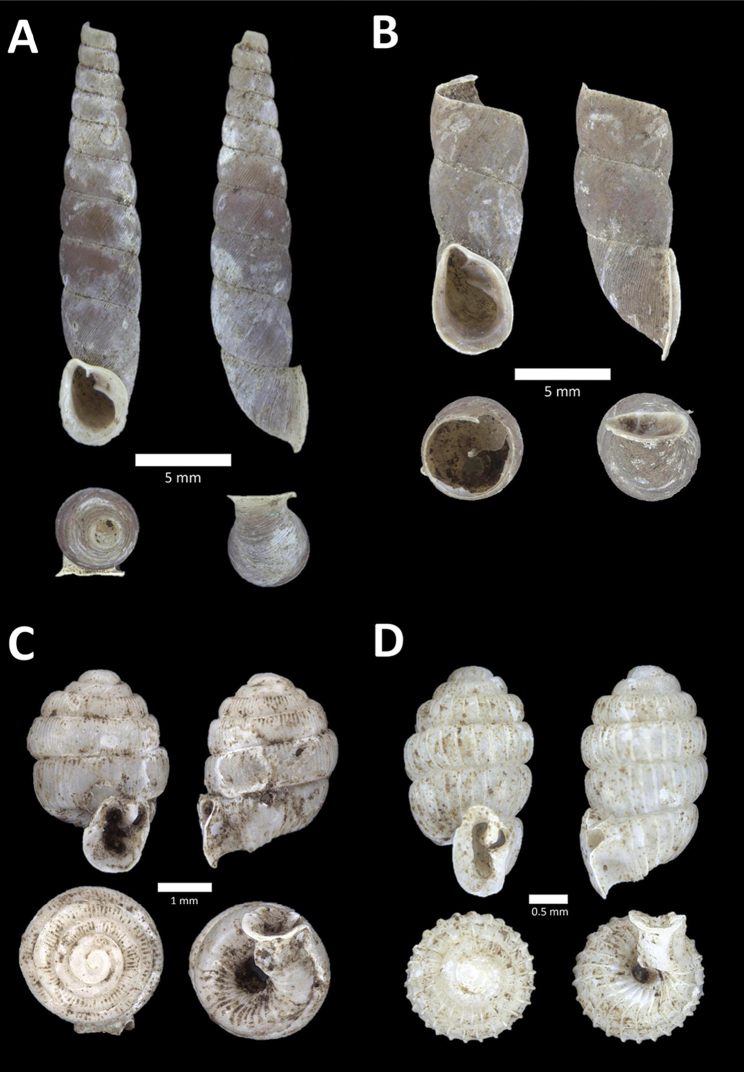
**A** *Phaedusa filicostata kapayanensis* (de Morgan, 1885b) BOR/MOL 10236. Perak, Ipoh, Gunung Rapat **B** *Phaedusa filicostata tenuicosta* (Nevill, 1878) BOR/MOL 10122. Perak, Ipoh, Gua Tok Giring Plot 2 **C** *Sinoennea* ‘guatokgiring 1’ BOR/MOL 10183. Perak, Ipoh, Gua Tok Giring Plot 4 **D** *Sinoennea hungerfordiana* (von Möllendorff, 1886) BOR/MOL 11009. Perak, Ipoh, Gunung Tasek.

######## Phaedusa filicostata tenuicosta

Taxon classificationAnimaliaStylommatophoraClausiliidae

(Nevill, 1878)

Figure 23B

######### Materials examined.

Prk 64 Bt Kepala Gajah: BOR/MOL 10122, BOR/MOL 10091, BOR/MOL 10170. mykarst-184 Bat Cave: BOR/MOL 9791, BOR/MOL 9824. Prk 53 Hill KF: BOR/MOL 10782. Prk 55 G. Pondok: BOR/MOL 11553.

######### Distribution.

In Peninsular Malaysia, known from Perak and Selangor (Maassen 2001). Elsewhere, in Pattani, Thailand (Maassen 2001).

######### Remarks.

Medium-sized shell, larger in all aspects compared to *Phaedusa filicostata kapayanensis* (Loosjes 1953). Lamella less pronounced compared to *P. filicostata kapayanensis*.

###### Family Diapheridae Panha & Naggs, in Sutcharit, Naggs, Wade, Fontanilla and Panha 2010

####### Genus *Sinoennea* Kobelt, 1904

######## Sinoennea

Taxon classificationAnimaliaStylommatophoraDiapheridae

‘guatokgiring 1’

Figure 23C

######### Materials examined.

Prk 64 Bt Kepala Gajah: BOR/MOL 10183.

######### Distribution.

Known from Bukit Kepala Gajah, Perak only, but surrounding hills have yet to be adequately surveyed.

######### Remarks.

Radial rib sculpture distinct. Distinguished from congeners by the arrangement of lamella, its gradually inflated antepenultimate and penultimate whorls and an ultimate whorl aligned to the right of the previous whorls. Davison (1991) labelled this species as “Sinoennea sp. cf. chrysallis”.

######## Sinoennea hungerfordiana

Taxon classificationAnimaliaStylommatophoraDiapheridae

(von Möllendorff, 1886)

Figure 23D

######### Materials examined.

Prk 53 Hill KF: BOR/MOL 10707, BOR/MOL 10719, BOR/MOL 10752, BOR/MOL 10665. mykarst-025: BOR/MOL 9395. Prk 34 G. Tasek: BOR/MOL 11158, BOR/MOL 11009, BOR/MOL 11045. Prk 55 G. Pondok: BOR/MOL 11497, BOR/MOL 11520, BOR/MOL 11559.

######### Distribution.

Known from Gunung Pondok, Perak and Bukit Baling, Kedah only (Maassen 2001, Tanmuangpak et al. 2015).

######### Remarks.

Distinguished from congeners by its widely-spaced radial ribs, less number of whorls and arrangement of the lamella (van Benthem Jutting 1961b).

######## Sinoennea lenggongensis

Taxon classificationAnimaliaStylommatophoraDiapheridae

Tomlin, 1939

Figure 24A

######### Materials examined.

Prk 64 Bt Kepala Gajah: BOR/MOL 10099, BOR/MOL 10141, BOR/MOL 10182.

######### Distribution.

Known from Lenggong and Kramat Pulai, Perak only (Maassen 2001).

######### Remarks.

Distinguished from congeners by its very cylindrical, tall spire shell, dense radial ribbing and slightly concaved central whorls (van Benthem Jutting 1961b).

**Figure 24.**
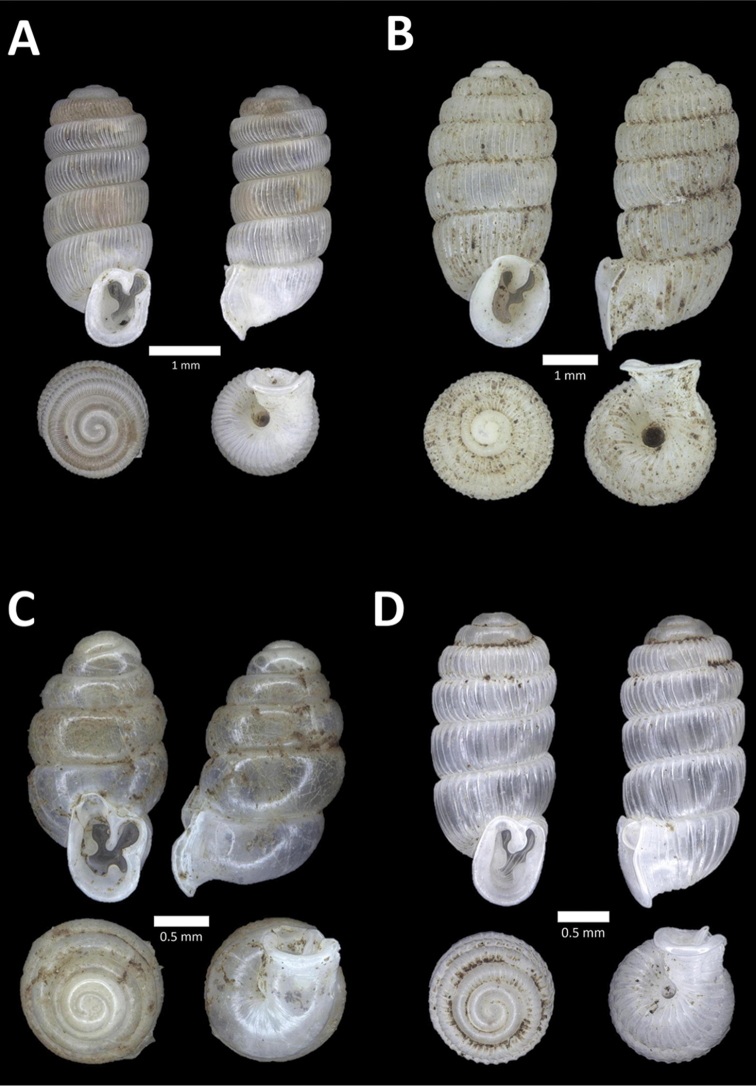
**A** *Sinoennea lenggongensis* Tomlin, 1939 BOR/MOL 10099. Perak, Ipoh, Gua Tok Giring Plot 1 **B** *Sinoennea perakensis* (Godwin-Austen & Nevill, 1879) BOR/MOL 11512. Perak, Ipoh, Gunung Pondok **C** *Sinoennea* ‘prk53 1’ BOR/MOL 10779. Perak, Ipoh, “Prk 53 Hill KF” plot 4 **D** *Sinoennea subcylindrica* (von Möllendorff, 1891) BOR/MOL 9779. Perak, Ipoh, Bat Cave Hill Plot 1

######## Sinoennea perakensis

Taxon classificationAnimaliaStylommatophoraDiapheridae

(Godwin-Austen & Nevill, 1879)

Figure 24B

######### Materials examined.

Prk 55 G. Pondok: BOR/MOL 11498, BOR/MOL 11512, BOR/MOL 11561, BOR/MOL 11580.

######### Distribution.

In Peninsular Malaysia, known from Gunung Pondok and Gapis Pass, Perak (Maassen 2001). Elsewhere, in Jalor (=Yala), Thailand (Maassen 2001).

######### Remarks.

Differ from sympatric *Sinoennea hungerfordiana* by its taller, straight, cylindrical shell, more whorls, more expanded peristome, lamella arrangement and denser radial ribs (but not as dense as *Sinoennea lenggongensis*).

######## Sinoennea

Taxon classificationAnimaliaStylommatophoraDiapheridae

‘prk53 1’

Figure 24C

######### Materials examined.

Prk 53 Hill KF: BOR/MOL 10735, BOR/MOL 10779.

######### Distribution.

Known only from Prk 53 Hill KF only, but surrounding hills have yet to be adequately surveyed.

######### Remarks.

Shell smallest among Perak congeners. Distinguished from Perak cogeners by its smooth whorls, absence of radial ribs and unique lamella arrangement.

######## Sinoennea subcylindrica

Taxon classificationAnimaliaStylommatophoraDiapheridae

(von Möllendorff, 1891)

Figure 24D

######### Materials examined.

Prk 47 Kanthan: BOR/MOL 9075, BOR/MOL 9165, BOR/MOL 9169. mykarst-027: BOR/MOL 9034, BOR/MOL 9116. mykarst-184 Bat Cave: BOR/MOL 9866, BOR/MOL 9779, BOR/MOL 9814, BOR/MOL 9846. Prk 23 G. Rapat: BOR/MOL 10213. Prk 42 G. Bercham: BOR/MOL 9236. mykarst-185 Batu Kebelah: BOR/MOL 9545, BOR/MOL 9750. Prk 36 Gua Datok: BOR/MOL 10423, BOR/MOL 10455. Prk 55 G. Pondok: BOR/MOL 11496, BOR/MOL 11521, BOR/MOL 11560, BOR/MOL 11579.

######### Distribution.

Known from Gunung Pondok, central and upper Kinta Valley, Perak only.

######### Remarks.

Most similar to *Sinoennea lenggongensis* but differ in apical whorl shape, less dense radial ribs and lamella arrangement.

######## Sinoennea tweediei

Taxon classificationAnimaliaStylommatophoraDiapheridae

Tomlin, 1941

Figure 25A

######### Materials examined.

Prk 64 Bt Kepala Gajah: BOR/MOL 10100, BOR/MOL 10140, BOR/MOL 10184.

######### Distribution.

Known from Lenggong and Temengor, Perak only (Maassen 2001).

######### Remarks.

Distinguished from Perak congeners by the box-like shell shape, fewer whorls of gradually increasing height, widely spaced radial ribs, flared peristome and peristomal structures.

**Figure 25.**
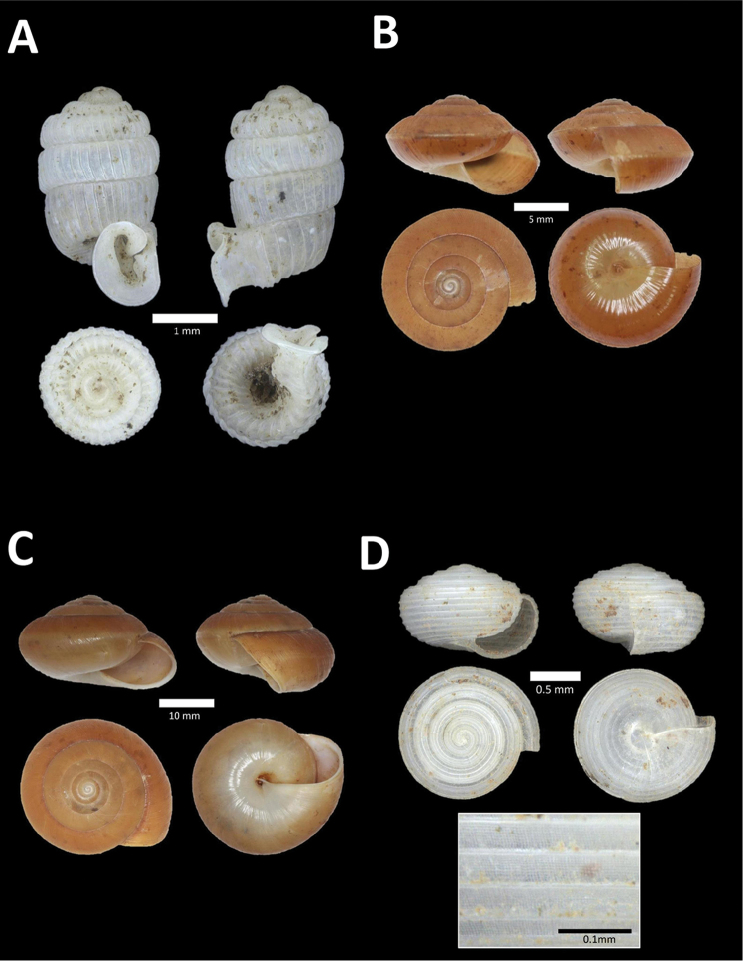
**A** *Sinoennea tweediei* Tomlin, 1941 BOR/MOL 10100. Perak, Ipoh, Gua Tok Giring Plot 1 **B** *Pseudoplecta bijuga* (Stoliczka, 1873) BOR/MOL 11213. Perak, Ipoh, Gunung Tempurung Plot 3 **C** *Quantula striata* (Gray, 1834) BOR/MOL 9857. Perak, Ipoh, Bat Cave Hill Plot 4 **D** *Glyptaulax* ‘tempurung 1’ BOR/MOL 11147. Perak, Ipoh, Gunung Tempurung Plot 1.

###### Family Dyakiidae Gude & Woodward, 1921

####### Genus *Pseudoplecta* Laidlaw, 1932

######## Pseudoplecta bijuga

Taxon classificationAnimaliaStylommatophoraDyakiidae

(Stoliczka, 1873)

Figure 25B

######### Materials examined.

mykarst-184 Bat Cave: BOR/MOL 9856, BOR/MOL 9764, BOR/MOL 9796, BOR/MOL 9826. Prk 53 Hill KF: BOR/MOL 10710, BOR/MOL 10739, BOR/MOL 10660, BOR/MOL 10687. mykarst-025: BOR/MOL 9375, BOR/MOL 9403, BOR/MOL 9435, BOR/MOL 9502. Prk 47 Kanthan: BOR/MOL 9065, BOR/MOL 9138. mykarst-027: BOR/MOL 9100. Prk 23 G. Rapat: BOR/MOL 10227, BOR/MOL 10044, BOR/MOL 10049, BOR/MOL 10251. Prk 42 G. Bercham: BOR/MOL 9213, BOR/MOL 9231, BOR/MOL 10571, BOR/MOL 10590, BOR/MOL 10616. Prk 36 Gua Datok: BOR/MOL 10431, BOR/MOL 10443, BOR/MOL 10478, BOR/MOL 10495. Prk 34 G. Tasek: BOR/MOL 10790, BOR/MOL 11168, BOR/MOL 11052. Prk 01 G. Tempurung: BOR/MOL 11133, BOR/MOL 11213, BOR/MOL 11242, BOR/MOL 11418. Prk 55 G. Pondok: BOR/MOL 11507, BOR/MOL 11480, BOR/MOL 11540.

######### Distribution.

In Peninsular Malaysia, found in Kedah (Langkawi), Penang, Perak, Selangor and Pahang (Cameron Highlands) (Laidlaw 1932, Maassen 2001). Elsewhere, in Nawngchik (=Pattani) and Rhaman (=Yala), Thailand (Maassen 2001).

######### Remarks.

Shell medium-sized, brown. Periphery strongly keeled. Dorsal whorls have dense and pronounced sradial ribbing while the umbilical side is glossy with fine radial growth lines.

####### Genus *Quantula* Baker, 1941

######## Quantula striata

Taxon classificationAnimaliaStylommatophoraDyakiidae

(Gray, 1834)

Figure 25C

######### Materials examined.

mykarst-184 Bat Cave: BOR/MOL 9857, BOR/MOL 9827. mykarst-027: BOR/MOL 9092. mykarst-185 Batu Kebelah: BOR/MOL 9122, BOR/MOL 9526, BOR/MOL 9557. Prk 47 Kanthan: BOR/MOL 9142. mykarst-025: BOR/MOL 9503. Prk 42 G. Bercham: BOR/MOL 10569.

######### Distribution.

Found throughout Peninsular Malaysia (Maassen 2001). Elsewhere, on Sundaland and the Philippines (Maassen 2001, Brodie and Barker 2012). Also, introduced into Fiji (Brodie and Barker 2012).

######### Remarks.

Shell medium-sized. Brown and non-glossy at dorsal whorls, white and glossy at umbilicus. Periphery keeled but less pronounced than *Pseudoplecta bijuga*. Whorls have dense and fine radial growth lines. This is a synanthropic species.

###### Family Endodontidae Pilsbry, 1895

####### Genus *Glyptaulax* Gude, 1914

######## Glyptaulax

Taxon classificationAnimaliaStylommatophoraEndodontidae

‘tempurung 1’

Figure 25D

######### Materials examined.

Prk 01 G. Tempurung: BOR/MOL 11147, BOR/MOL 11220, BOR/MOL 11391, BOR/MOL 11424.

######### Distribution.

Known from Gunung Tempurung only, but surrounding hills have yet to be adequately surveyed.

######### Remarks.

Shell small. Spire low, shell discoid. Distinguished from other discoid snails by the presence of strong spiral ridges of equal width. In between these spiral ridges, fine round pits are deposited in a neat matrix. Differs from *Glyptaulax artificiosa* (Benson, 1856) by its less expanded aperture and smaller umbilicus. First record of *Glyptaulax* in Peninsular Malaysia.

####### Genus *Philalanka* Godwin-Austen, 1898

######## Philalanka kusana

Taxon classificationAnimaliaStylommatophoraEndodontidae

(Aldrich, 1889)

Figure 26A

######### Materials examined.

Prk 53 Hill KF: BOR/MOL 10702, BOR/MOL 10755, BOR/MOL 10769, BOR/MOL 10729, BOR/MOL 10672. Prk 36 Gua Datok: BOR/MOL 10060. Prk 23 G. Rapat: BOR/MOL 10284, BOR/MOL 10285. Prk 42 G. Bercham: BOR/MOL 10578. Prk 34 G. Tasek: BOR/MOL 11161, BOR/MOL 11017, BOR/MOL 11041, BOR/MOL 11174. Prk 55 G. Pondok: BOR/MOL 11488, BOR/MOL 11530, BOR/MOL 11546, BOR/MOL 11572. Prk 01 G. Tempurung: BOR/MOL 11223, BOR/MOL 11399.

######### Distribution.

In Peninsular Malaysia, known from Pahang (Maassen 2001) and Perak. Elsewhere, ranges from Sumatra to Maluku, in Indonesia (Vermeulen and Whitten 1998).

######### Remarks.

Distinguished from *Philalanka pusilla* by the white shell, taller spire, two to three major spiral ridges with many minor spiral lines and less changes in the angularity of whorls. New record for Perak.

**Figure 26.**
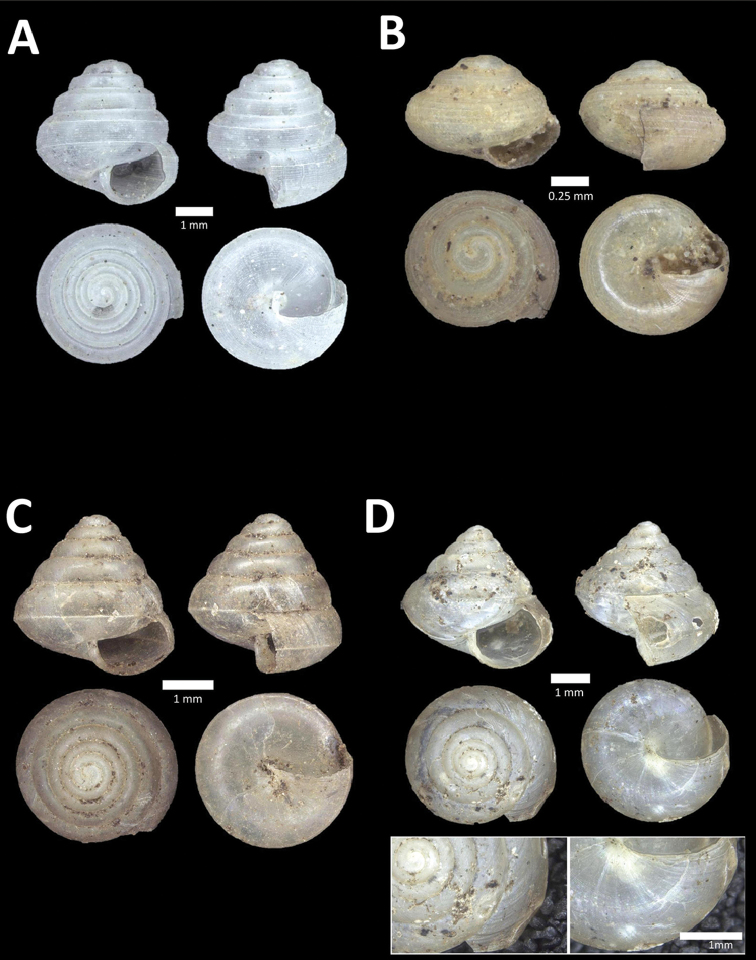
**A** *Philalanka kusana* (Aldrich, 1889) BOR/MOL 11174. Perak, Ipoh, Gunung Tasek Plot 5 **B** *Philalanka pusilla* Maassen, 2000 BOR/MOL 9865. Perak, Ipoh, Bat Cave Hill Plot 4 **C** *Kaliella barrakporensis* (Pfeiffer, 1852) BOR/MOL 9032. Perak, Ipoh, Gunung Kanthan Plot 1 **D** *Kaliella calculosa* (Gould, 1852) BOR/MOL 10053. Perak, Ipoh, Gunung Rapat Plot C3.

######## Philalanka pusilla

Taxon classificationAnimaliaStylommatophoraEndodontidae

Maassen, 2000

Figure 26B

######### Materials examined.

Prk 53 Hill KF: BOR/MOL 10705, BOR/MOL 10750, BOR/MOL 10673. mykarst-184 Bat Cave: BOR/MOL 9865.mykarst-027: BOR/MOL 9045, BOR/MOL 9128. Prk 47 Kanthan: BOR/MOL 9452, BOR/MOL 9154. Prk 36 Gua Datok: BOR/MOL 10063, BOR/MOL 10424, BOR/MOL 10460. Prk 23 G. Rapat: BOR/MOL 10266. Prk 34 G. Tasek: BOR/MOL 11124, BOR/MOL 11188. Prk 01 G. Tempurung: BOR/MOL 11219.

######### Distribution.

In Peninsular Malaysia, known from Kinta Valley, Perak. Elsewhere, in Sumatra, Indonesia (Maassen 2000).

######### Remarks.

Shell smaller and flatter than *Philalanka kusana*, spire low. Shell yellow to light brown. Five or more major spiral ridges and more changes in angularity of whorls. New record for Peninsular Malaysia.

###### Family Euconulidae Baker, 1928

####### Genus *Kaliella* Blanford, 1863

######## Kaliella barrakporensis

Taxon classificationAnimaliaStylommatophoraEuconulidae

(Pfeiffer, 1852)

Figure 26C

######### Materials examined.

mykarst-027: BOR/MOL 9032, BOR/MOL 9107. Prk 53 Hill KF: BOR/MOL 10744, BOR/MOL 10728, BOR/MOL 10679, BOR/MOL 10694. mykarst-184 Bat Cave: BOR/MOL 9869, BOR/MOL 9830, BOR/MOL 9773, BOR/MOL 9808. mykarst-025: BOR/MOL 9388, BOR/MOL 9411, BOR/MOL 9485, BOR/MOL 9510, BOR/MOL 12432. Prk 47 Kanthan: BOR/MOL 9111, BOR/MOL 9153. Prk 42 G. Bercham: BOR/MOL 9471, BOR/MOL 9475, BOR/MOL 9220, BOR/MOL 10579, BOR/MOL 10600, BOR/MOL 10619, BOR/MOL 10626. Prk 23 G. Rapat: BOR/MOL 10210, BOR/MOL 10030, BOR/MOL 10243, BOR/MOL 10268. mykarst-185 Batu Kebelah: BOR/MOL 9531, BOR/MOL 9587. Prk 36 Gua Datok: BOR/MOL 10062, BOR/MOL 10433, BOR/MOL 10456. Prk 64 Bt Kepala Gajah: BOR/MOL 10102, BOR/MOL 10143, BOR/MOL 10178. Prk 55 G. Pondok: BOR/MOL 11489, BOR/MOL 11532, BOR/MOL 11547, BOR/MOL 11573. Prk 34 G. Tasek: BOR/MOL 11016, BOR/MOL 11034, BOR/MOL 11056, BOR/MOL 11172. Prk 01 G. Tempurung: BOR/MOL 11203, BOR/MOL 11218, BOR/MOL 11388, BOR/MOL 11432.

######### Distribution.

In Peninsular Malaysia, known from Perak only although it is likely widespread. Elsewhere, distributed from Africa in the west to Flores, Indonesia in the east (Vermeulen et al. 2015).

######### Remarks.

Shell small, conical. Differ from congeners in its fine but distinct radial sculpture, less dense coiling of whorls and slightly convex whorls.

######## Kaliella calculosa

Taxon classificationAnimaliaStylommatophoraEuconulidae

(Gould, 1852)

Figure 26D

######### Materials examined.

Prk 47 Kanthan: BOR/MOL 9185, BOR/MOL 9163. mykarst-027: BOR/MOL 9036, BOR/MOL 9108. mykarst-184 Bat Cave: BOR/MOL 9772, BOR/MOL 9809. Prk 23 G. Rapat: BOR/MOL 10053. Prk 64 Bt Kepala Gajah: BOR/MOL 10096. Prk 55 G. Pondok: BOR/MOL 11574.

######### Distribution.

In Peninsular Malaysia, currently known from Penang, Perak and Selangor although it is likely widespread. Elsewhere, distributed from India in the west to Australia and Tahiti in the east (Vermeulen et al. 2015).

######### Remarks.

Shell slightly larger than *Kaliella barrakporensis*. Differs from sympatric congeners in its fewer but more convex whorls, especially obvious in the ultimate whorl. Radial sculpture fine but distinct. Spiral sculpture usually present.

######## Kaliella microconus

Taxon classificationAnimaliaStylommatophoraEuconulidae

(Mousson, 1865)

Figure 27A

######### Materials examined.

Prk 53 Hill KF: BOR/MOL 10746, BOR/MOL 10692. mykarst-025: BOR/MOL 9516. mykarst-185 Batu Kebelah: BOR/MOL 9551. Prk 36 Gua Datok: BOR/MOL 10061, BOR/MOL 10467, BOR/MOL 10486. Prk 42 G. Bercham: BOR/MOL 10577, BOR/MOL 10601, BOR/MOL 10633. Prk 34 G. Tasek: BOR/MOL 11155, BOR/MOL 11012, BOR/MOL 11036, BOR/MOL 11055, BOR/MOL 11173. Prk 55 G. Pondok: BOR/MOL 11531, BOR/MOL 11583. Prk 01 G. Tempurung: BOR/MOL 11387, BOR/MOL 11433.

######### Distribution.

In Peninsular Malaysia, known from Perak, Johor (Pulau Aur) and Kelantan although it is likely widespread (Maassen 2001). Elsewhere, from Sundaland to Australia, Fiji and Samoa (Vermeulen et al. 2015).

######### Remarks.

Distinguished from *Kaliella barrakporensis* by its straighter spire, less convex whorls and predominant spiral sculpture.

**Figure 27.**
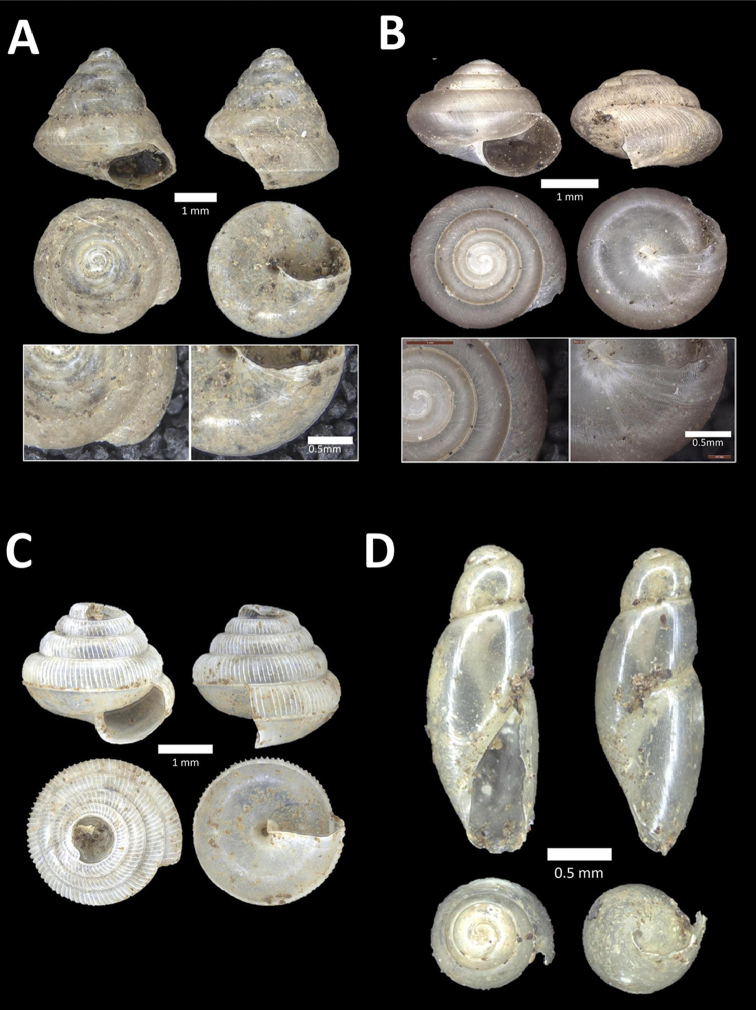
**A** *Kaliella microconus* (Mousson, 1865) BOR/MOL 9551. Perak, Ipoh, Batu Kebelah Plot 1 **B** *Kaliella scandens* (Cox, 1871) BOR/MOL 11043. Perak, Ipoh, Gunung Tasek Plot 1 **C** *Rahula* ‘tempurung 1’ BOR/MOL 11392. Perak, Ipoh, Gunung Tempurung Plot 2 **D** *Cecilioides caledonica* (Crosse, 1867) BOR/MOL 9748. Perak, Ipoh, Batu Kebelah Plot 4.

######## Kaliella scandens

Taxon classificationAnimaliaStylommatophoraEuconulidae

(Cox, 1871)

Figure 27B

######### Materials examined.

Prk 47 Kanthan: BOR/MOL 9076, BOR/MOL 9155, BOR/MOL 9167. mykarst-027: BOR/MOL 9040, BOR/MOL 9131. Prk 53 Hill KF: BOR/MOL 10751, BOR/MOL 10763, BOR/MOL 10731, BOR/MOL 10668, BOR/MOL 10693. mykarst-184 Bat Cave: BOR/MOL 9870, BOR/MOL 9786, BOR/MOL 9819, BOR/MOL 9836. mykarst-025: BOR/MOL 9392, BOR/MOL 9424, BOR/MOL 9517. Prk 42 G. Bercham: BOR/MOL 9480, BOR/MOL 9221, BOR/MOL 10582, BOR/MOL 10603, BOR/MOL 10618, BOR/MOL 10638. Prk 23 G. Rapat: BOR/MOL 10211, BOR/MOL 10045, BOR/MOL 10276, BOR/MOL 10282. mykarst-185 Batu Kebelah: BOR/MOL 9536, BOR/MOL 9590. Prk 36 Gua Datok: BOR/MOL 10058, BOR/MOL 10425. Prk 64 Bt Kepala Gajah: BOR/MOL 10103, BOR/MOL 10151, BOR/MOL 10177. Prk 34 G. Tasek: BOR/MOL 11019, BOR/MOL 11164, BOR/MOL 11004, BOR/MOL 11035, BOR/MOL 11043, BOR/MOL 11044, BOR/MOL 11057, BOR/MOL 11175, BOR/MOL 11184, BOR/MOL 11185. Prk 55 G. Pondok: BOR/MOL 11491, BOR/MOL 11529, BOR/MOL 11545, BOR/MOL 11571. Prk 01 G. Tempurung: BOR/MOL 11204, BOR/MOL 11225, BOR/MOL 11390, BOR/MOL 11425.

######### Distribution.

Widespread in Peninsular Malaysia (Vermeulen et al. 2015). Elsewhere, from Sundaland to Australia and the Pacific islands (Vermeulen et al. 2015).

######### Remarks.

Shell shape similar to *Kaliella doliolum* (Pfeiffer, 1846). Radial rib density highly variable, from very fine growth lines to coarse ribs. Distinguished from *K. doliolum* by the presence of fine spiral lines and absent or indistinct radial ribs at the umbilical section of the whorls. This is a synanthropic species.

####### Genus *Rahula* Godwin-Austen, 1907

######## Rahula

Taxon classificationAnimaliaStylommatophoraEuconulidae

‘tempurung 1’

Figure 27C

######### Materials examined.

Prk 01 G. Tempurung: BOR/MOL 11207, BOR/MOL 11222, BOR/MOL 11392, BOR/MOL 11422.

######### Distribution.

Known from Gunung Tempurung only, but surrounding hills have yet to be adequately surveyed.

######### Remarks.

Distinguished from other Southeast Asian congeners by its convex whorls at both dorsal and umbilical sides, sharp peripheral keel, dense and pronounced radial ribs as well as glossy, spiral lined umbilical whorls. First record of *Rahula* in Peninsular Malaysia.

###### Family Ferussaciidae Bourguignat, 1883

####### Genus *Cecilioides* Férussac, 1814

######## Cecilioides caledonica

Taxon classificationAnimaliaStylommatophoraFerussaciidae

(Crosse, 1867)

Figure 27D

######### Materials examined.

mykarst-185 Batu Kebelah: BOR/MOL 9748. Prk 01 G. Tempurung: BOR/MOL 11421.

######### Distribution.

In Peninsular Malaysia, known from Perlis and Perak (Maassen 2001). Elsewhere, in Indonesia (Java), New Guinea and New Caledonia (Maassen 2001).

######### Remarks.

A distinctive species with a thin, bullet-shaped shell. Spire tall, suture indistinct, whorls smooth and glossy. Aperture elongated and narrow.

###### Family Helicarionidae Bourguignat, 1877

####### Genus *Helicarion* Férussac, 1821

######## Helicarion permolle

Taxon classificationAnimaliaStylommatophoraHelicarionidae

Stoliczka, 1873

Figure 28A

######### Materials examined.

mykarst-184 Bat Cave: BOR/MOL 9766, BOR/MOL 9805. Prk 53 Hill KF: BOR/MOL 10734, BOR/MOL 10664. mykarst-025: BOR/MOL 9377, BOR/MOL 9414, BOR/MOL 9492. Prk 47 Kanthan: BOR/MOL 9055, BOR/MOL 9152. mykarst-027: BOR/MOL 9097. Prk 42 G. Bercham: BOR/MOL 9240, BOR/MOL 10597. Prk 36 Gua Datok: BOR/MOL 10434, BOR/MOL 10484. Prk 01 G. Tempurung: BOR/MOL 11148, BOR/MOL 11230, BOR/MOL 11248. Prk 34 G. Tasek: BOR/MOL 11179. Prk 55 G. Pondok: BOR/MOL 11542, BOR/MOL 11567.

######### Distribution.

Known from Penang and Perak only (Maassen 2001).

######### Remarks.

Shell small. Periphery rounded, whorls few, spire low. Surface glossy with very fine spiral lines. Sutural line distinct and white.

**Figure 28.**
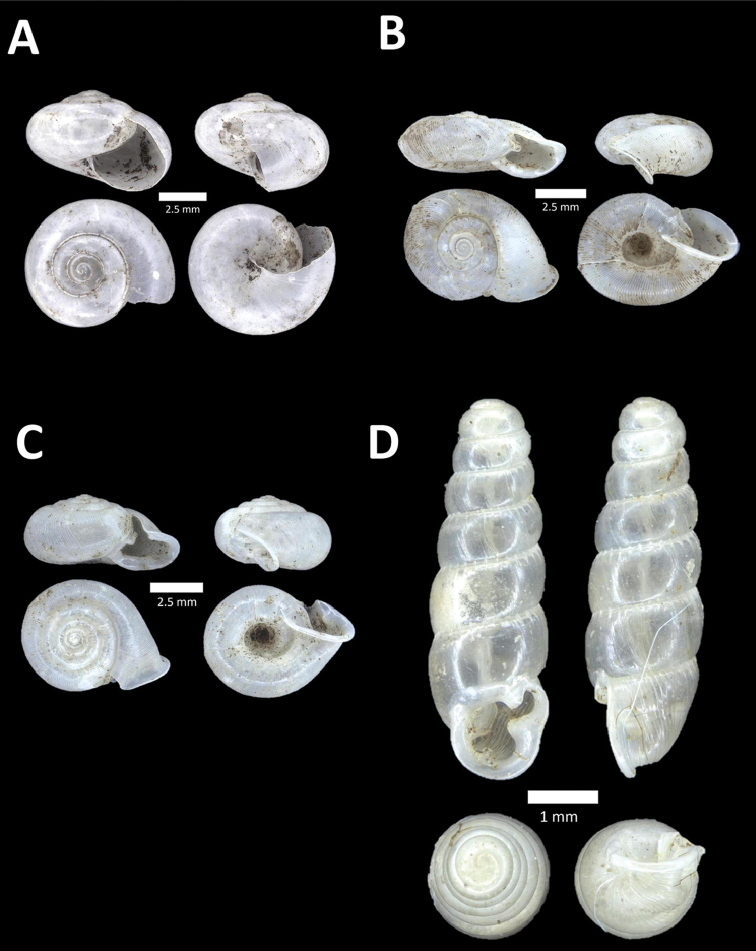
**A** *Helicarion permolle* Stoliczka, 1873 BOR/MOL 9152. Perak, Ipoh, Gunung Kanthan Plot 4 **B** *Discartemon leptoglyphus* van Benthem Jutting, 1954 BOR/MOL 10479. Perak, Ipoh, Gunung Datok **C** *Discartemon plussensis* (de Morgan, 1885a) BOR/MOL 11031. Perak, Ipoh, Gunung Tasek

###### Family Streptaxidae Gray, 1860

####### Genus *Discartemon* Pfeiffer, 1856

######## Discartemon leptoglyphus

Taxon classificationAnimaliaStylommatophoraStreptaxidae

van Benthem Jutting, 1954

Figure 28B

######### Materials examined.

mykarst-027: BOR/MOL 9110, BOR/MOL 9013.mykarst-025: BOR/MOL 9412. Prk 42 G. Bercham: BOR/MOL 9466, BOR/MOL 10596. Prk 47 Kanthan: BOR/MOL 9143. Prk 23 G. Rapat: BOR/MOL 10048, BOR/MOL 10205, BOR/MOL 10232, BOR/MOL 10254. Prk 36 Gua Datok: BOR/MOL 10418, BOR/MOL 10446, BOR/MOL 10479, BOR/MOL 10492. Prk 34 G. Tasek: BOR/MOL 10794.

######### Distribution.

Restricted to central Kinta Valley, Perak (Maassen 2001, Siriboon et al. 2014).

######### Remarks.

Shell size varies. Differs from *Discatemon plussensis* in the prominence of lamella, presence of a peripheral keel and flatter shell.

######## Discartemon plussensis

Taxon classificationAnimaliaStylommatophoraStreptaxidae

(de Morgan, 1885a)

Figure 28C

######### Materials examined.

Prk 47 Kanthan: BOR/MOL 9056. Prk 42 G. Bercham: BOR/MOL 9458, BOR/MOL 9473, BOR/MOL 9476, BOR/MOL 9217, BOR/MOL 10620, BOR/MOL 10630. Prk 53 Hill KF: BOR/MOL 10695. Prk 23 G. Rapat: BOR/MOL 10260. Prk 34 G. Tasek: BOR/MOL 10795, BOR/MOL 11167, BOR/MOL 11031, BOR/MOL 11176. mykarst-027: BOR/MOL 9015. Prk 01 G. Tempurung: BOR/MOL 11145, BOR/MOL 11381, BOR/MOL 11413.

######### Distribution.

Known from Perak only (Maassen 2001, Siriboon et al. 2014).

######### Remarks.

Shell size varies. Differs from *Discatemon leptoglyphus* in the prominence of lamella, the absence of a peripheral keel and having more bulbous whorls. Prior to this, Davison (1991) reported *D. plussensis* from Gunung Tchehel and a few hills near Sungai Siput North only.

####### Genus *Gulella* Pfeiffer, 1856

######## Gulella bicolor

Taxon classificationAnimaliaStylommatophoraStreptaxidae

(Hutton, 1834)

Figure 28D

######### Materials examined.

mykarst-184 Bat Cave: BOR/MOL 9879. Prk 47 Kanthan: BOR/MOL 9159, BOR/MOL 9195. mykarst-185 Batu Kebelah: BOR/MOL 9540, BOR/MOL 9586.

######### Distribution.

Found throughout Peninsular Malaysia (Maassen 2001). Elsewhere, pantropical (Simone 2013).

######### Remarks.

Radial ribs faint to distinct. Distinguished from confamilals by its tall spire, cylindrical shell, compact whorls without detachment and its lamella arrangement. This is a synanthropic species.

####### Family Subulinidae Fischer & Crosse, 1877

######## Genus *Allopeas* Baker, 1935

######### Allopeas clavulinum

Taxon classificationAnimaliaStylommatophoraSubulinidae

(Potiez & Michaud, 1838)

Figure 29A

########## Materials examined.

Prk 53 Hill KF: BOR/MOL 10700, BOR/MOL 10753, BOR/MOL 10722, BOR/MOL 10674. mykarst-184 Bat Cave: BOR/MOL 9876, BOR/MOL 9878, BOR/MOL 9801, BOR/MOL 9832, BOR/MOL 9769. Prk 47 Kanthan: BOR/MOL 9083, BOR/MOL 9145. mykarst-027: BOR/MOL 9037, BOR/MOL 9119. mykarst-025: BOR/MOL 9393, BOR/MOL 9409, BOR/MOL 9487, BOR/MOL 9512. Prk 42 G. Bercham: BOR/MOL 9465, BOR/MOL 9479, BOR/MOL 9215, BOR/MOL 10634, BOR/MOL 10635, BOR/MOL 10636. mykarst-185 Batu Kebelah: BOR/MOL 9539, BOR/MOL 9544, BOR/MOL 9583. Prk 64 Bt Kepala Gajah: BOR/MOL 10169. Prk 36 Gua Datok: BOR/MOL 10419, BOR/MOL 10487, BOR/MOL 10505. Prk 55 G. Pondok: BOR/MOL 11614. Prk 34 G. Tasek: BOR/MOL 11020, BOR/MOL 11160, BOR/MOL 11033, BOR/MOL 11038, BOR/MOL 11183. Prk 01 G. Tempurung: BOR/MOL 11150, BOR/MOL 11237, BOR/MOL 11386, BOR/MOL 11395, BOR/MOL 11430, BOR/MOL 11431.

########## Distribution.

In Peninsular Malaysia, found in Perak and Kelantan but likely more widespread (Maassen 2001). Elsewhere, possibly native to tropical East Africa but now pantropical (Ho 1995, Vermeulen and Whitten 1998).

########## Remarks.

Shell small, spire tall. Whorls more obtuse than *Allopeas gracile*. Spiral growth lines fine. This is a synanthropic species.

**Figure 29.**
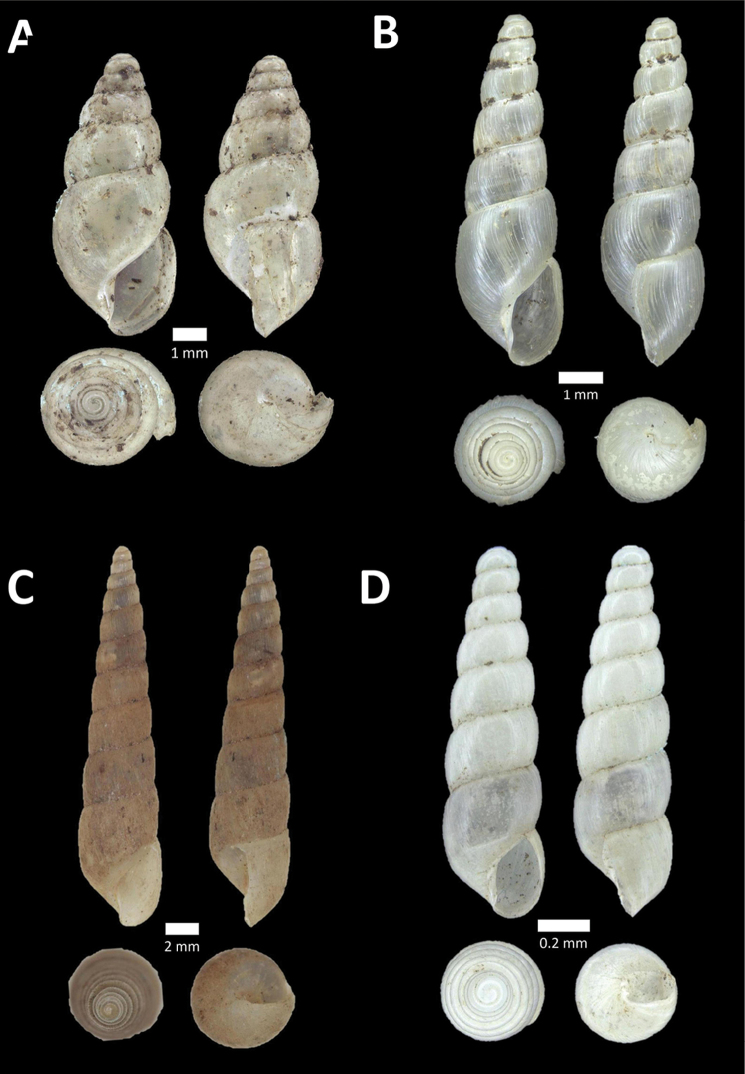
**A** *Allopeas clavulinum* (Potiez & Michaud, 1838) BOR/MOL 9409. Perak, Ipoh, Mykarst-025 Plot 2 **B** *Allopeas gracile* (Hutton, 1834) BOR/MOL 9112. Perak, Ipoh, Gunung Kanthan Plot 3 **C** *Prosopeas tchehelense* (de Morgan, 1885b) BOR/MOL 9098. Perak, Ipoh, Gunung Kanthan Plot 2 **D** *Subulina octona* (Bruguière, 1792) BOR/MOL 11227. Perak, Ipoh, Gunung Tempurung Plot 3.

######### Allopeas gracile

Taxon classificationAnimaliaStylommatophoraSubulinidae

(Hutton, 1834)

Figure 29B

########## Materials examined.

Prk 53 Hill KF: BOR/MOL 10699, BOR/MOL 10678. mykarst-027: BOR/MOL 9085, BOR/MOL 9121. mykarst-184 Bat Cave: BOR/MOL 9867, BOR/MOL 9802, BOR/MOL 9837, BOR/MOL 9768. mykarst-025: BOR/MOL 9394, BOR/MOL 9408, BOR/MOL 9440, BOR/MOL 9513. Prk 47 Kanthan: BOR/MOL 9112, BOR/MOL 9146. Prk 42 G. Bercham: BOR/MOL 9214, BOR/MOL 10607. mykarst-185 Batu Kebelah: BOR/MOL 9541, BOR/MOL 9749. Prk 64 Bt Kepala Gajah: BOR/MOL 10089, BOR/MOL 10144, BOR/MOL 10145, BOR/MOL 10181. Prk 36 Gua Datok: BOR/MOL 10427, BOR/MOL 10461, BOR/MOL 10501. Prk 55 G. Pondok: BOR/MOL 11613. Prk 01 G. Tempurung: BOR/MOL 11143, BOR/MOL 11226, BOR/MOL 11398, BOR/MOL 11415. Prk 34 G. Tasek: BOR/MOL 11157, BOR/MOL 11006, BOR/MOL 11037, BOR/MOL 11180.

########## Distribution.

Found throughout Peninsular Malaysia (Maassen 2001). Elsewhere, pantropical (Raheem et al. 2014).

########## Remarks.

Shell small, spire tall. Whorls slender than *Allopeas clavulinum*. Spiral growth lines fine. This is a synanthropic species.

######## Genus Paropeas Pilsbry, 1906

######### Paropeas tchehelense

Taxon classificationAnimaliaStylommatophoraSubulinidae

(de Morgan, 1885b)

Figure 29C

########## Materials examined.

Prk 64 Bt Kepala Gajah: BOR/MOL 10121, BOR/MOL 10087, BOR/MOL 10131, BOR/MOL 10165. mykarst-184 Bat Cave: BOR/MOL 9767, BOR/MOL 9797, BOR/MOL 9831. mykarst-025: BOR/MOL 9384, BOR/MOL 9406, BOR/MOL 9439, BOR/MOL 9508, BOR/MOL 12430. Prk 47 Kanthan: BOR/MOL 9050, BOR/MOL 9144. mykarst-027: BOR/MOL 9098, BOR/MOL 9014. Prk 23 G. Rapat: BOR/MOL 10209, BOR/MOL 10032, BOR/MOL 10047, BOR/MOL 10233, BOR/MOL 10257. mykarst-185 Batu Kebelah: BOR/MOL 9162, BOR/MOL 9543. Prk 42 G. Bercham: BOR/MOL 9216, BOR/MOL 10581, BOR/MOL 10591. Prk 36 Gua Datok: BOR/MOL 10059, BOR/MOL 10428, BOR/MOL 10451. Prk 53 Hill KF: BOR/MOL 10675. Prk 34 G. Tasek: BOR/MOL 10792, BOR/MOL 11029, BOR/MOL 11186. Prk 01 G. Tempurung: BOR/MOL 11146, BOR/MOL 11383, BOR/MOL 11416. Prk 55 G. Pondok: BOR/MOL 11511, BOR/MOL 11483, BOR/MOL 11550, BOR/MOL 11565.

########## Distribution.

In Peninsular Malaysia, known from Perak, Selangor, Pahang and Kelantan (Maassen 2001). Elsewhere, in Jalor (=Yala), Thailand (Maassen 2001).

########## Remarks.

Distinguished from all confamilials by its large shell, rounded apical whorls, fine but pronounced radial ribs and angular lower periphery.

######## Genus *Subulina* Beck, 1837

######### Subulina octona

Taxon classificationAnimaliaStylommatophoraSubulinidae

(Bruguière, 1792)

Figure 29D

########## Materials examined.

mykarst-184 Bat Cave: BOR/MOL 9875. mykarst-025: BOR/MOL 9410. mykarst-185 Batu Kebelah: BOR/MOL 9542, BOR/MOL 9564, BOR/MOL 9574, BOR/MOL 9584. Prk 23 G. Rapat: BOR/MOL 10255. Prk 34 G. Tasek: BOR/MOL 10791, BOR/MOL 11027. Prk 01 G. Tempurung: BOR/MOL 11227, BOR/MOL 11389.

########## Distribution.

Found throughout Peninsular Malaysia (Maassen 2001). Elsewhere, pantropical (Raheem et al. 2014).

########## Remarks.

Distinguished from all confamilials by its glossy, large shell, very convex whorls, fine radial growth lines and truncated columella.

####### Family Trochomorphidae von Möllendorff, 1890

######## Genus *Videna* Adams & Adams, 1855

######### Videna castra

Taxon classificationAnimaliaStylommatophoraTrochomorphidae

(Benson, 1852b)

Figure 30A

########## Materials examined.

Prk 47 Kanthan: BOR/MOL 9077, BOR/MOL 9172. mykarst-027: BOR/MOL 9090. Prk 53 Hill KF: BOR/MOL 10716, BOR/MOL 10663, BOR/MOL 10688. Prk 64 Bt Kepala Gajah: BOR/MOL 10093, BOR/MOL 10162. Prk 23 G. Rapat: BOR/MOL 10287. Prk 36 Gua Datok: BOR/MOL 10422, BOR/MOL 10457, BOR/MOL 10497. Prk 42 G. Bercham: BOR/MOL 10608. Prk 01 G. Tempurung: BOR/MOL 11135, BOR/MOL 11245, BOR/MOL 11384, BOR/MOL 11417. Prk 34 G. Tasek: BOR/MOL 11169. Prk 55 G. Pondok: BOR/MOL 11509, BOR/MOL 11481, BOR/MOL 11549, BOR/MOL 11570. mykarst-025: BOR/MOL 12429.

########## Distribution.

Found throughout Peninsular Malaysia (Maassen 2001). Elsewhere, in Darjiling (=Darjeeling), India and Salang (=Phuket), Thailand (Maassen 2001).

########## Remarks.

Distinguished from Peninsular Malaysian congeners by its distinct spiral lines at apical whorls, becoming less at post-apical whorls. Radial sculpture more prominent at post-apical whorls.

**Figure 30.**
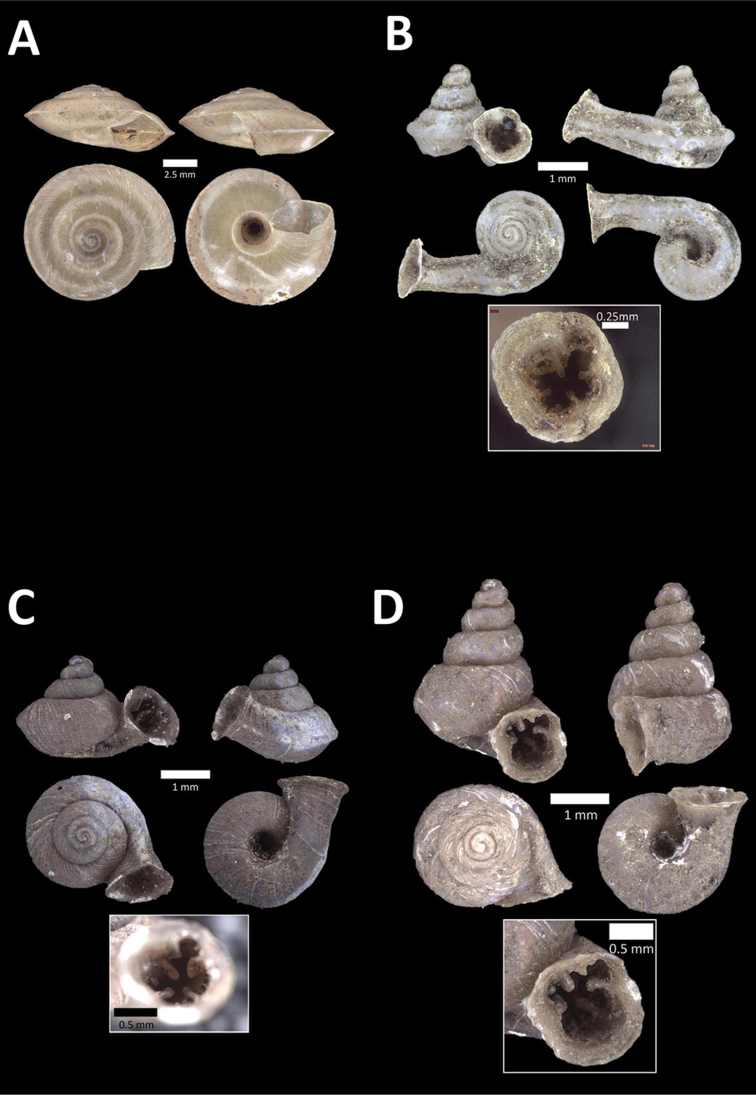
**A** *Videna castra* (Benson, 1852b) BOR/MOL 11135. Perak, Ipoh, Gunung Tempurung Plot 1 **B** *Gyliotrachela hungerfordiana* (von Möllendorff, 1886) BOR/MOL 9133. Perak, Ipoh, Gunung Kanthan **C** *Gyliotrachela luctans* van Benthem Jutting, 1950 BOR/MOL 10064. Perak, Ipoh, Gunung Datok **D** *Paraboysidia oreia* van Benthem Jutting, 1961 BOR/MOL 10504. Perak, Ipoh, Gunung

####### Family Hypselostomatidae Zilch, 1959

######## Genus *Gyliotrachela* Tomlin, 1930

######### Gyliotrachela hungerfordiana

Taxon classificationAnimaliaStylommatophoraHypselostomatidae

(von Möllendorff, 1886)

Figure 30B

########## Materials examined.

Prk 53 Hill KF: BOR/MOL 10701, BOR/MOL 10743, BOR/MOL 10726, BOR/MOL 10667. Prk 47 Kanthan: BOR/MOL 9073, BOR/MOL 9148. mykarst-184 Bat Cave: BOR/MOL 9871, BOR/MOL 9789, BOR/MOL 9804, BOR/MOL 9845. mykarst-025: BOR/MOL 9390, BOR/MOL 9419. Prk 42 G. Bercham: BOR/MOL 9461, BOR/MOL 9464, BOR/MOL 9483, BOR/MOL 10575, BOR/MOL 10602, BOR/MOL 10623. mykarst-027: BOR/MOL 9133, BOR/MOL 9241. mykarst-185 Batu Kebelah: BOR/MOL 9538, BOR/MOL 9747. Prk 23 G. Rapat: BOR/MOL 10040, BOR/MOL 10234, BOR/MOL 10262. Prk 64 Bt Kepala Gajah: BOR/MOL 10094, BOR/MOL 10142, BOR/MOL 10180. Prk 36 Gua Datok: BOR/MOL 10426, BOR/MOL 10458, BOR/MOL 10507. Prk 34 G. Tasek: BOR/MOL 11156, BOR/MOL 11002, BOR/MOL 11046, BOR/MOL 11181. Prk 55 G. Pondok: BOR/MOL 11513, BOR/MOL 11486, BOR/MOL 11548, BOR/MOL 11568.

########## Distribution.

Found on limestone karsts across Peninsular Malaysia (Maassen 2001). Elsewhere, in southern Thailand (Maassen 2003).

########## Remarks.

Distinct among congeners in its tall spire, keeled periphery, very long trumpet-shaped ultimate whorl and apertural teeth arrangement. Shell colour varies from dark to light brown.

######### Gyliotrachela luctans

Taxon classificationAnimaliaStylommatophoraHypselostomatidae

van Benthem Jutting, 1950

Figure 30C

########## Materials examined.

Prk 36 Gua Datok: BOR/MOL 10064.

########## Distribution.

Known from Gunung Pondok (van Benthem Jutting 1950) and Gua Datok, Perak only.

########## Remarks.

Spire tall but fewer than *Gyliotrachela hungerfordiana*. Penultimate whorl very expanded and periphery keeled, giving a depressed shell shape. Ultimate whorl trumpet-shaped, with aperture face at 45 degrees of the plane perpendicular to the coiling axis. Apertural teeth arrangement unique.

######## Genus *Paraboysidia* Pilsbry, 1917

######### Paraboysidia oreia

Taxon classificationAnimaliaStylommatophoraHypselostomatidae

van Benthem Jutting, 1961a

Figure 30D

########## Materials examined.

Prk 53 Hill KF: BOR/MOL 10727. Prk 47 Kanthan: BOR/MOL 9064, BOR/MOL 9175. mykarst-027: BOR/MOL 9125, BOR/MOL 9022. Prk 36 Gua Datok: BOR/MOL 10504. Prk 34 G. Tasek: BOR/MOL 11013, BOR/MOL 11039, BOR/MOL 11060.

########## Distribution.

Known from Gunung Batu Kurau (van Benthem Jutting 1961) and Kinta Valley, Perak only.

########## Remarks.

Shell small, dark brown. Spire tall. Whorls rather convex. Distinguished from Perak congeners by the slightly keeled periphery and apertural teeth arrangement.

######### Paraboysidia

Taxon classificationAnimaliaStylommatophoraHypselostomatidae

‘tempurung 1’

Figure 31A

########## Materials examined.

Prk 01 G. Tempurung: BOR/MOL 11217.

########## Distribution.

Known from Gunung Tempurung, Perak only, but surrounding hills have yet to be adequately surveyed.

########## Remarks.

Differ from *Paraboysidia kelantanensis tenuidentata* van Benthem Jutting, 1950, of Kramat Pulai by its gradually flatter and more expanded whorls, periphery rounded, shallow suture, short trumpet-like extension of ultimate whorl and apertural teeth arrangement.

**Figure 31.**
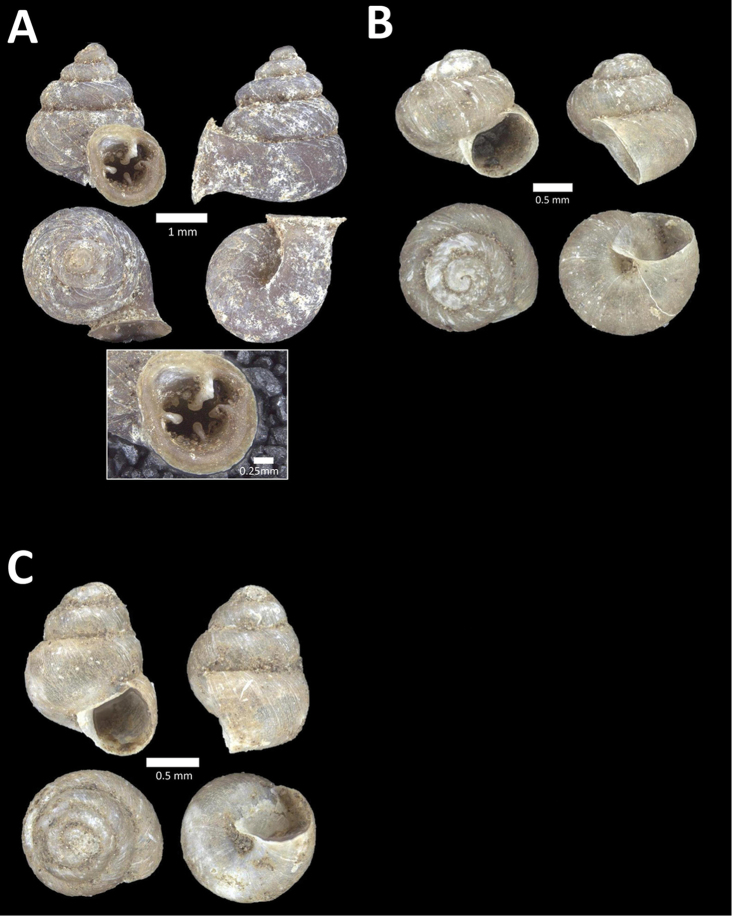
**A** *Paraboysidia* ‘tempurung 1’ BOR/MOL 11217. Perak, Ipoh, Gunung Tempurung Plot 3 **B** *Ptychopatula orcula* (Benson, 1850) BOR/MOL 9043. Perak, Ipoh, Gunung Kanthan Plot 1 **C** *Ptychopatula solemi* Maassen, 2000 BOR/MOL 9042. Perak, Ipoh, Gunung Kanthan Plot 1.

####### Family Vallonidae Morse, 1864

######## Genus *Ptychopatula* Pilsbry, 1889

######### Ptychopatula orcula

Taxon classificationAnimaliaStylommatophoraVallonidae

(Benson, 1850)

Figure 31B

########## Materials examined.

Prk 53 Hill KF: BOR/MOL 10708, BOR/MOL 10723. mykarst-184 Bat Cave: BOR/MOL 9862, BOR/MOL 9782, BOR/MOL 9815, BOR/MOL 9842. Prk 47 Kanthan: BOR/MOL 9078, BOR/MOL 9171. mykarst-027: BOR/MOL 9043, BOR/MOL 9118. mykarst-025: BOR/MOL 9391, BOR/MOL 9426, BOR/MOL 9488, BOR/MOL 9522. Prk 42 G. Bercham: BOR/MOL 9457, BOR/MOL 9463, BOR/MOL 9478, BOR/MOL 9219, BOR/MOL 10584, BOR/MOL 10599, BOR/MOL 10621, BOR/MOL 10631, BOR/MOL 10632. Prk 23 G. Rapat: BOR/MOL 10218, BOR/MOL 10036, BOR/MOL 10235, BOR/MOL 10269. mykarst-185 Batu Kebelah: BOR/MOL 9537, BOR/MOL 9743. Prk 64 Bt Kepala Gajah: BOR/MOL 10104, BOR/MOL 10148, BOR/MOL 10185. Prk 36 Gua Datok: BOR/MOL 10429, BOR/MOL 10471, BOR/MOL 10503. Prk 01 G. Tempurung: BOR/MOL 11144, BOR/MOL 11224, BOR/MOL 11426. Prk 34 G. Tasek: BOR/MOL 11163, BOR/MOL 11008, BOR/MOL 11042, BOR/MOL 11182. Prk 55 G. Pondok: BOR/MOL 11487, BOR/MOL 11526, BOR/MOL 11551.

########## Distribution.

In Peninsular Malaysia, known from Perlis and Perak (Maassen 2001). Elsewhere, in the tropical regions of the Indian Ocean and the Pacific Islands (Seddon 2000).

########## Remarks.

Distinguished from sympatric congener *Ptychopatula solemi* by its larger shell, wider whorls, wide umbilicus and lower spire.

######### Ptychopatula solemi

Taxon classificationAnimaliaStylommatophoraVallonidae

Maassen, 2000

Figure 31C

########## Materials examined.

Prk 47 Kanthan: BOR/MOL 9176, BOR/MOL 9068. mykarst-027: BOR/MOL 9042. mykarst-184 Bat Cave: BOR/MOL 9864, BOR/MOL 9818, BOR/MOL 9841. mykarst-185 Batu Kebelah: BOR/MOL 9548, BOR/MOL 9753. Prk 23 G. Rapat: BOR/MOL 10239. Prk 34 G. Tasek: BOR/MOL 11000.

########## Distribution.

In Peninsular Malaysia, known from Perak only (Maassen 2001). Elsewhere, in Sumatra and Sulawesi, Indonesia (Maassen 2000).

########## Remarks.

Distinguished from sympatric congener *Ptychopatula orcula* by its smaller shell, tighter whorls, narrower umbilicus and tall spire.

## Discussion

Apart from providing a comprehensive assessment of land snail diversity for the limestone hills in and around the Kinta Valley of Perak, our study has also elucidated interesting biogeographical patterns to assist in conservation planning. Our study has also paved the way for more in-depth land snail taxonomy studies to be conducted. For instance, many of the land snail species recorded by Davison (1991) and Clements et al. (2008b) could not be assigned to scientific names that have been published, until now. Speciose genera such as *Microcystina*, *Diplommatina* and *Opisthostoma*, require critical taxonomic revisions that need considerable effort. We hope unnamed species in these genera, now with traceable reference specimens collected from our study, can be described in the near future.

### Land snail species diversity and biogeographical patterns

Correlation tests initially suggested no statistically significant relationship between the number of unique species and the degree of limestone hill isolation. However, when examined closely within a geographical context, the number of unique species for each hill could also be associated with the degree of isolation of major limestone clusters of each hill (Table 1). For example, Prk 55 G. Pondok, Prk 64 Bt Kepala Gajah, and Prk 01 G. Tempurung all located in the limestone clusters at the periphery of Kinta Valley and at least 50 km away from other limestone clusters outside of Kinta Valley (see Figure 6 in Liew et al. 2016).

At this point in time, our assignment of unique species should be considered “unique” to a particular hill in the context of hills that have been surveyed systematically in our study. The degree of endemism of a species depends on the geographical context, e.g. endemic to Malaysia, Perak, Kinta Valley, hill clusters or a single hill. For example, *Charopa lafargei* which previously presumed as endemic to Gunung Kanthan (Vermeulen and Marzuki 2014), is shown in our study to also occur on the limestone hills at the north of Kinta Valley. Also, *Hydrocena semisculpta* which was thought be endemic to Gunung Pondok (Davison 1991), is a relatively widespread species – can be found in 11 out of the 12 hills surveyed in our study.

Our comprehensive inventory has corroborated Davison’s (1991) hypothesis that land snail assemblages on limestone hills in Perak can be divided into two groups, one comprising of hills within Kinta Valley and the other comprising of hills scattered in the area north of Kinta Valley. This biogeographical pattern cannot be explained by geographical distance between different limestone hills alone. For example, although Bat Cave Hill and Batu Kebelah Hill (northern cluster) are closest to Prk 53 Hill KF, the land snail assemblage of Prk 53 Hill KF is more similar to the land snail assemblages in the southern cluster hills.

It seems that biogeographical patterns of land snail assemblages at limestone hills in and around the Kinta Valley of Perak were influenced by the geology and topography of the area. For instance, all the hills in the southern cluster are located in the low-lying floodplains of the Kinta valley, while the hills of the northern cluster are located on hilly granitic areas north of the Kinta valley floodplain. As such, this biogeographical pattern could be a result of geological isolation (de Morgan 1885a; van Benthem Jutting 1960; Davison 1991).

The rocks in Perak (including limestone) first emerged in the Mesozoic – the Late Jurassic period (van Benthem Jutting 1960; Metcalfe 2013). After that, limestone hills in Perak became isolated due to two different processes that produced the two biogeographical regions (i.e. limestone hill clusters) (Paton 1961). For the northern hill cluster, the isolation of hills was likely to be caused by structural deformity with intrusion of granite and these hills were probably never contiguous from the start (Paton 1961). For the southern hill cluster, the isolation of hills was probably caused by limestone erosion, recent marine incursion and alluvial deposition (Paton 1961). However, our hypothesis of geological isolation being the main determinant of biogeographical patterns observed in our study requires further tests using phylogeographic approaches, where the divergence time of a particular land snail species can be estimated (by sampling populations from different limestone hills) and matched with historical geological events, including the more recent local geological events or climate changes in the Pleistocene.

### Conservation implications

Despite containing the second highest number of limestone hills in Peninsular Malaysia after the State of Kelantan (see Liew et al. 2016), the State of Perak appears to be experiencing the greatest threat from limestone quarrying with 45 out of 78 limestone quarries in the peninsula being located in this State as of 2014 (Minerals and Geoscience Department Malaysia 2014). As such, there is an urgent need to identify limestone hills with high levels of species endemism for conservation prioritisation within this State. Four hills, namely Prk 01 G. Tempurung, Prk 55 G. Pondok, Prk 47 Kanthan, and Prk 64 Bt Kepala Gajah, were found to collectively contain 91% of the total land snail species occurring at 12 hills sampled in our study. In order to prevent the extinction of unique land snail species, these hills require immediate attention from conservation practitioners to mitigate ongoing threats from quarrying and surrounding forest loss.

## Conclusion

Our study has updated the state of knowledge of land snail diversity and biogeography on limestone hills in Perak, Peninsular Malaysia. Larger limestone hills seem to have more unique species, of which several could be site endemic. In addition, we have shown that land snail assemblages can be divided into two groups that occupy limestone hills at two different areas in Perak – this was likely caused by different geological processes that separated the limestone hills. Most importantly, our findings can be used to assist in limestone conservation planning, especially within the Kinta Valley of Perak.

## Supplementary materials

Supplementary material 1 Sample completeness curve for all the seven limestone hills sampled in our study. Data type: Text and figures in Microsoft Word file. Explanation note: Data and figure outputs from species richness and sampling completeness analysis. This dataset is made available under the Open Database License (http://opendatacommons.org/licenses/odbl/1.0/). The Open Database License (ODbL) is a license agreement intended to allow users to freely share, modify, and use this Dataset while maintaining this same freedom for others, provided that the original source and author(s) are credited.Junn Kitt Foon, Gopalasamy Reuben Clements, Thor-Seng Liew

Supplementary material 2 Data matrix for absence/presence of 122 land snail species at 12 limestone hills. Data type: Microsoft Excel datasheet. Explanation note: Data matrix for absence/presence of 122 land snail species at 12 limestone hills. This dataset is made available under the Open Database License (http://opendatacommons.org/licenses/odbl/1.0/). The Open Database License (ODbL) is a license agreement intended to allow users to freely share, modify, and use this Dataset while maintaining this same freedom for others, provided that the original source and author(s) are credited.Junn Kitt Foon, Gopalasamy Reuben Clements, Thor-Seng Liew

Supplementary material 3 Location of sampling plots for each of the seven hills sampled in our study. Data type: Text in Microsoft Word file. Explanation note: A list of the location names and coordinates of sampling plots for each of the seven hills sampled in our study. This dataset is made available under the Open Database License (http://opendatacommons.org/licenses/odbl/1.0/). The Open Database License (ODbL) is a license agreement intended to allow users to freely share, modify, and use this Dataset while maintaining this same freedom for others, provided that the original source and author(s) are credited.Junn Kitt Foon, Gopalasamy Reuben Clements, Thor-Seng Liew
