# Supplementary material for: Diversity and biogeography of land snails (Mollusca, Gastropoda) in the limestone hills of Perak, Peninsular Malaysia
Source: Zookeys. 2017 Jul 4;(682):1–94. doi: 10.3897/zookeys.682.12999 (PMC5523159; doi:10.3897/zookeys.682.12999)

**Appendix 3.** Sample completeness curve for all the seven limestone hills sampled in our study.

| **site** | **Species richness** | **Sampling Completeness** |
| --- | --- | --- |
| Bat Cave Hill | 50 | 0.9354 |
| Batu Kebelah | 39 | 0.9726 |
| Gua Tok Giring | 45 | 0.8671 |
| Gunung Bercham | 28 | 0.8435 |
| Gunung Kanthan | 63 | 0.9805 |
| Gunung Tempurung | 54 | 0.9619 |
| Mykarst-025 | 39 | 0.9056 |


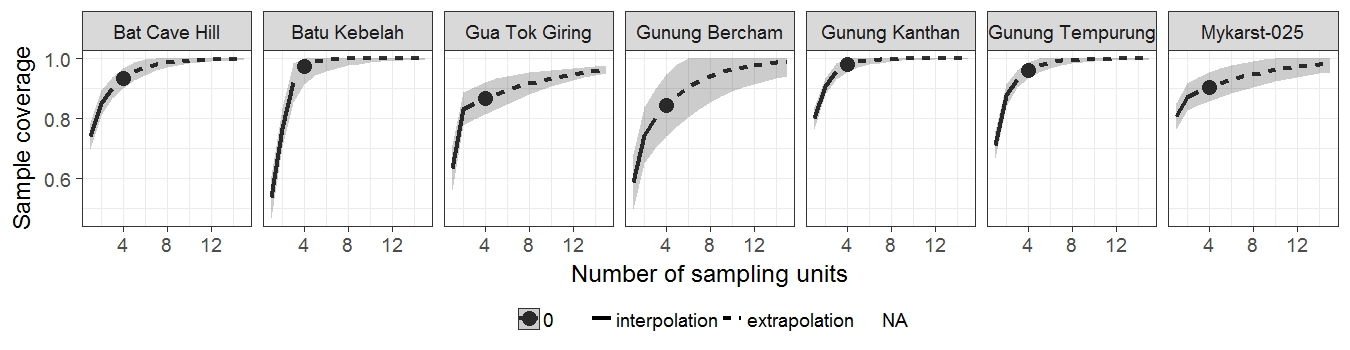

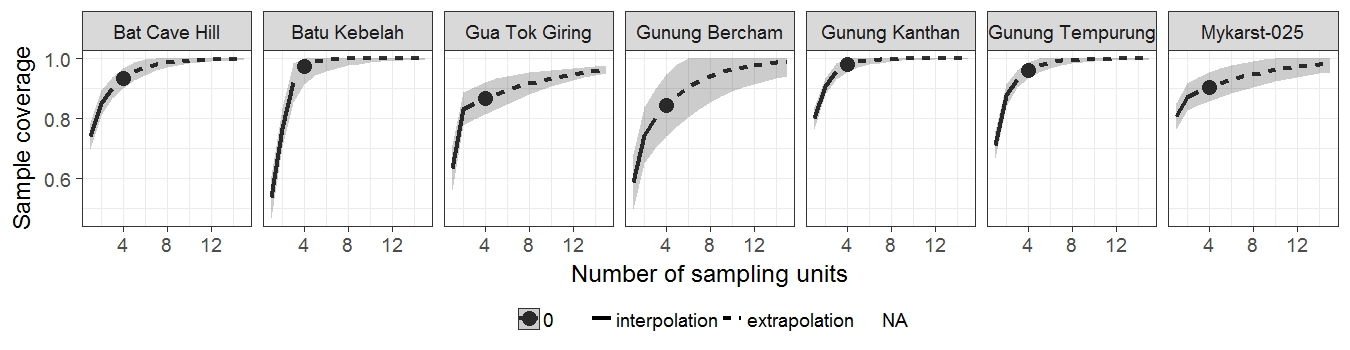

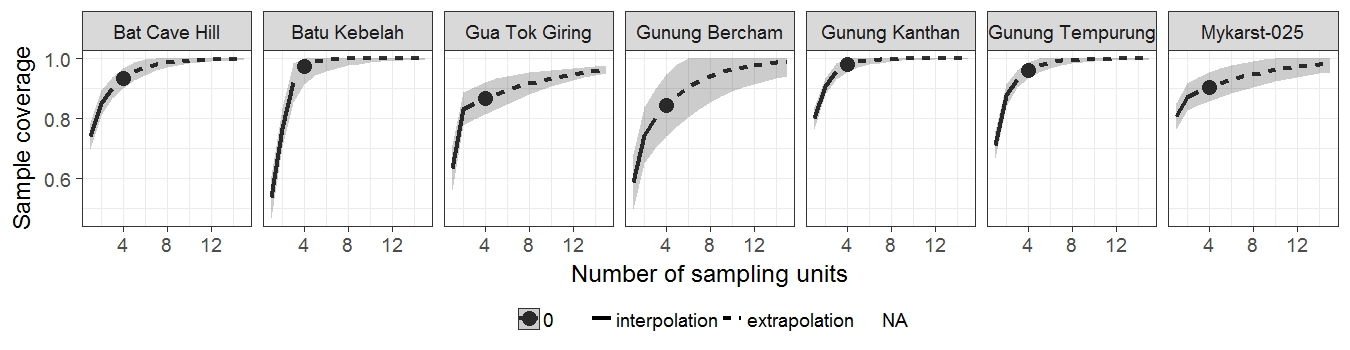

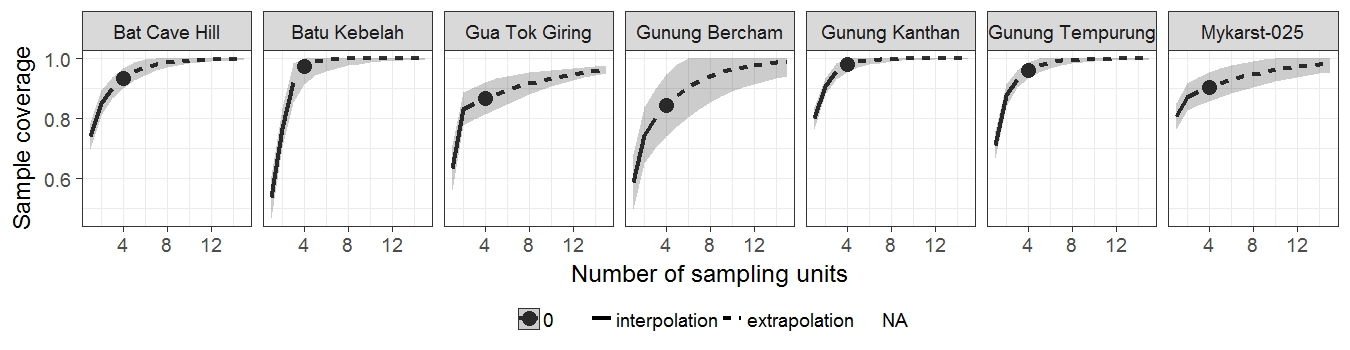

Supplement: Supplementary material 1 — Sample completeness curve for all the seven limestone hills sampled in our study. [file zookeys-682-001-s001.docx]
