# Supplementary material for: Diversity and biogeography of land snails (Mollusca, Gastropoda) in the limestone hills of Perak, Peninsular Malaysia
Source: Zookeys. 2017 Jul 4;(682):1–94. doi: 10.3897/zookeys.682.12999 (PMC5523159; doi:10.3897/zookeys.682.12999)
Supplement: Supplementary material 3 — Location of sampling plots for each of the seven hills sampled in our study. [file zookeys-682-001-s003.docx]

**Appendix 1.** Location of sampling plots for each of the seven hills sampled in our study.

| Plot name | Longitude | Latitude |
| --- | --- | --- |
| Bat Cave Hill Plot 1 | 101.1466 | 4.905832 |
| Bat Cave Hill Plot 2 | 101.1471 | 4.905751 |
| Bat Cave Hill Plot 3 | 101.147 | 4.907049 |
| Bat Cave Hill Plot 4 | 101.148 | 4.907207 |
| Batu Kebelah Plot 1 | 101.1859 | 4.853674 |
| Batu Kebelah Plot 2 | 101.1856 | 4.853329 |
| Batu Kebelah Plot 3 | 101.1858 | 4.854164 |
| Batu Kebelah Plot 4 | 101.1867 | 4.854476 |
| Gua Tok Giring Plot 1 | 100.9729 | 5.118568 |
| Gua Tok Giring Plot 2 | 100.9729 | 5.118928 |
| Gua Tok Giring Plot 3 | 100.9723 | 5.11935 |
| Gua Tok Giring Plot 4 | 100.9717 | 5.119606 |
| Gunung Bercham Plot 1 | 101.1339 | 4.645417 |
| Gunung Bercham Plot 2 | 101.1341 | 4.645127 |
| Gunung Bercham Plot 3 | 101.1336 | 4.644835 |
| Gunung Bercham Plot 4 | 101.1332 | 4.645064 |
| Gunung Kanthan Plot 1 | 101.1211 | 4.78158 |
| Gunung Kanthan Plot 2 | 101.1216 | 4.782369 |
| Gunung Kanthan Plot 3 | 101.1211 | 4.761053 |
| Gunung Kanthan Plot 4 | 101.1211 | 4.7616 |
| Gunung Tempurung Plot 1 | 101.188 | 4.415721 |
| Gunung Tempurung Plot 2 | 101.1877 | 4.417446 |
| Gunung Tempurung Plot 3 | 101.1872 | 4.418478 |
| Gunung Tempurung Plot 4 | 101.1895 | 4.413303 |
| Mykarst-025 Plot 1 | 101.1544 | 4.655358 |
| Mykarst-025 Plot 2 | 101.1541 | 4.654455 |
| Mykarst-025 Plot 3 | 101.1539 | 4.653296 |
| Mykarst-025 Plot 4 | 101.154 | 4.654981 |
